# Measurements of production cross sections of the Higgs boson in the four-lepton final state in proton–proton collisions at $\sqrt{s} = 13\,\text {TeV}$

**DOI:** 10.1140/epjc/s10052-021-09200-x

**Published:** 2021-06-02

**Authors:** CMS Collaboration, A. M. Sirunyan, A. Tumasyan, W. Adam, J. W. Andrejkovic, T. Bergauer, S. Chatterjee, M. Dragicevic, A. Escalante Del Valle, R. Frühwirth, M. Jeitler, N. Krammer, L. Lechner, D. Liko, I. Mikulec, F. M. Pitters, J. Schieck, R. Schöfbeck, M. Spanring, S. Templ, W. Waltenberger, C.-E. Wulz, V. Chekhovsky, A. Litomin, V. Makarenko, M. R. Darwish, E. A. De Wolf, X. Janssen, T. Kello, A. Lelek, H. Rejeb Sfar, P. Van Mechelen, S. Van Putte, N. Van Remortel, F. Blekman, E. S. Bols, J. D’Hondt, J. De Clercq, M. Delcourt, S. Lowette, S. Moortgat, A. Morton, D. Müller, A. R. Sahasransu, S. Tavernier, W. Van Doninck, P. Van Mulders, D. Beghin, B. Bilin, B. Clerbaux, G. De Lentdecker, L. Favart, A. Grebenyuk, A. K. Kalsi, K. Lee, I. Makarenko, L. Moureaux, L. Pétré, A. Popov, N. Postiau, E. Starling, L. Thomas, C. Vander Velde, P. Vanlaer, D. Vannerom, L. Wezenbeek, T. Cornelis, D. Dobur, M. Gruchala, G. Mestdach, M. Niedziela, C. Roskas, K. Skovpen, M. Tytgat, W. Verbeke, B. Vermassen, M. Vit, A. Bethani, G. Bruno, F. Bury, C. Caputo, P. David, C. Delaere, I. S. Donertas, A. Giammanco, V. Lemaitre, K. Mondal, J. Prisciandaro, A. Taliercio, M. Teklishyn, P. Vischia, S. Wertz, S. Wuyckens, G. A. Alves, C. Hensel, A. Moraes, W. L. Aldá Júnior, M. Barroso Ferreira Filho, H. Brandao Malbouisson, W. Carvalho, J. Chinellato, E. M. Da Costa, G. G. Da Silveira, D. De Jesus Damiao, S. Fonseca De Souza, J. Martins, D. Matos Figueiredo, C. Mora Herrera, K. Mota Amarilo, L. Mundim, H. Nogima, P. Rebello Teles, L. J. Sanchez Rosas, A. Santoro, S. M. Silva Do Amaral, A. Sznajder, M. Thiel, F. Torres Da Silva De Araujo, A. Vilela Pereira, C. A. Bernardes, L. Calligaris, T. R. Fernandez Perez Tomei, E. M. Gregores, D. S. Lemos, P. G. Mercadante, S. F. Novaes, Sandra S. Padula, A. Aleksandrov, G. Antchev, I. Atanasov, R. Hadjiiska, P. Iaydjiev, M. Misheva, M. Rodozov, M. Shopova, G. Sultanov, A. Dimitrov, T. Ivanov, L. Litov, B. Pavlov, P. Petkov, A. Petrov, T. Cheng, W. Fang, Q. Guo, T. Javaid, M. Mittal, H. Wang, L. Yuan, M. Ahmad, G. Bauer, C. Dozen, Z. Hu, Y. Wang, K. Yi, E. Chapon, G. M. Chen, H. S. Chen, M. Chen, A. Kapoor, D. Leggat, H. Liao, Z.-A. LIU, R. Sharma, A. Spiezia, J. Tao, J. Thomas-wilsker, J. Wang, H. Zhang, S. Zhang, J. Zhao, A. Agapitos, Y. Ban, C. Chen, Q. Huang, A. Levin, Q. Li, M. Lu, X. Lyu, Y. Mao, S. J. Qian, D. Wang, Q. Wang, J. Xiao, Z. You, X. Gao, H. Okawa, M. Xiao, C. Avila, A. Cabrera, C. Florez, J. Fraga, A. Sarkar, M. A. Segura Delgado, J. Jaramillo, J. Mejia Guisao, F. Ramirez, J. D. Ruiz Alvarez, C. A. Salazar González, N. Vanegas Arbelaez, D. Giljanovic, N. Godinovic, D. Lelas, I. Puljak, Z. Antunovic, M. Kovac, T. Sculac, V. Brigljevic, D. Ferencek, D. Majumder, M. Roguljic, A. Starodumov, T. Susa, A. Attikis, E. Erodotou, A. Ioannou, G. Kole, M. Kolosova, S. Konstantinou, J. Mousa, C. Nicolaou, F. Ptochos, P. A. Razis, H. Rykaczewski, H. Saka, M. Finger, M. Finger, A. Kveton, J. Tomsa, E. Ayala, E. Carrera Jarrin, S. Abu Zeid, S. Khalil, E. Salama, M. A. Mahmoud, Y. Mohammed, S. Bhowmik, A. Carvalho Antunes De Oliveira, R. K. Dewanjee, K. Ehataht, M. Kadastik, J. Pata, M. Raidal, C. Veelken, P. Eerola, L. Forthomme, H. Kirschenmann, K. Osterberg, M. Voutilainen, E. Brücken, F. Garcia, J. Havukainen, V. Karimäki, M. S. Kim, R. Kinnunen, T. Lampén, K. Lassila-Perini, S. Lehti, T. Lindén, H. Siikonen, E. Tuominen, J. Tuominiemi, P. Luukka, H. Petrow, T. Tuuva, C. Amendola, M. Besancon, F. Couderc, M. Dejardin, D. Denegri, J. L. Faure, F. Ferri, S. Ganjour, A. Givernaud, P. Gras, G. Hamel de Monchenault, P. Jarry, B. Lenzi, E. Locci, J. Malcles, J. Rander, A. Rosowsky, M. Ö. Sahin, A. Savoy-Navarro, M. Titov, G. B. Yu, S. Ahuja, F. Beaudette, M. Bonanomi, A. Buchot Perraguin, P. Busson, C. Charlot, O. Davignon, B. Diab, G. Falmagne, R. Granier de Cassagnac, A. Hakimi, I. Kucher, A. Lobanov, C. Martin Perez, M. Nguyen, C. Ochando, P. Paganini, J. Rembser, R. Salerno, J. B. Sauvan, Y. Sirois, A. Zabi, A. Zghiche, J.-L. Agram, J. Andrea, D. Apparu, D. Bloch, G. Bourgatte, J.-M. Brom, E. C. Chabert, C. Collard, D. Darej, J.-C. Fontaine, U. Goerlach, C. Grimault, A.-C. Le Bihan, P. Van Hove, E. Asilar, S. Beauceron, C. Bernet, G. Boudoul, C. Camen, A. Carle, N. Chanon, D. Contardo, P. Depasse, H. El Mamouni, J. Fay, S. Gascon, M. Gouzevitch, B. Ille, Sa. Jain, I. B. Laktineh, H. Lattaud, A. Lesauvage, M. Lethuillier, L. Mirabito, K. Shchablo, L. Torterotot, G. Touquet, M. Vander Donckt, S. Viret, A. Khvedelidze, Z. Tsamalaidze, L. Feld, K. Klein, M. Lipinski, D. Meuser, A. Pauls, M. P. Rauch, J. Schulz, M. Teroerde, D. Eliseev, M. Erdmann, P. Fackeldey, B. Fischer, S. Ghosh, T. Hebbeker, K. Hoepfner, H. Keller, L. Mastrolorenzo, M. Merschmeyer, A. Meyer, G. Mocellin, S. Mondal, S. Mukherjee, D. Noll, A. Novak, T. Pook, A. Pozdnyakov, Y. Rath, H. Reithler, J. Roemer, A. Schmidt, S. C. Schuler, A. Sharma, S. Wiedenbeck, S. Zaleski, C. Dziwok, G. Flügge, W. Haj Ahmad, O. Hlushchenko, T. Kress, A. Nowack, C. Pistone, O. Pooth, D. Roy, H. Sert, A. Stahl, T. Ziemons, H. Aarup Petersen, M. Aldaya Martin, P. Asmuss, I. Babounikau, S. Baxter, O. Behnke, A. Bermúdez Martínez, A. A. Bin Anuar, K. Borras, V. Botta, D. Brunner, A. Campbell, A. Cardini, P. Connor, S. Consuegra Rodríguez, V. Danilov, M. M. Defranchis, L. Didukh, D. Domínguez Damiani, G. Eckerlin, D. Eckstein, L. I. Estevez Banos, E. Gallo, A. Geiser, A. Giraldi, A. Grohsjean, M. Guthoff, A. Harb, A. Jafari, N. Z. Jomhari, H. Jung, A. Kasem, M. Kasemann, H. Kaveh, C. Kleinwort, J. Knolle, D. Krücker, W. Lange, T. Lenz, J. Lidrych, K. Lipka, W. Lohmann, T. Madlener, R. Mankel, I.-A. Melzer-Pellmann, J. Metwally, A. B. Meyer, M. Meyer, J. Mnich, A. Mussgiller, V. Myronenko, Y. Otarid, D. Pérez Adán, S. K. Pflitsch, D. Pitzl, A. Raspereza, A. Saggio, A. Saibel, M. Savitskyi, V. Scheurer, C. Schwanenberger, A. Singh, R. E. Sosa Ricardo, N. Tonon, O. Turkot, A. Vagnerini, M. Van De Klundert, R. Walsh, D. Walter, Y. Wen, K. Wichmann, C. Wissing, S. Wuchterl, O. Zenaiev, R. Zlebcik, R. Aggleton, S. Bein, L. Benato, A. Benecke, K. De Leo, T. Dreyer, M. Eich, F. Feindt, A. Fröhlich, C. Garbers, E. Garutti, P. Gunnellini, J. Haller, A. Hinzmann, A. Karavdina, G. Kasieczka, R. Klanner, R. Kogler, V. Kutzner, J. Lange, T. Lange, A. Malara, A. Nigamova, K. J. Pena Rodriguez, O. Rieger, P. Schleper, M. Schröder, J. Schwandt, D. Schwarz, J. Sonneveld, H. Stadie, G. Steinbrück, A. Tews, B. Vormwald, I. Zoi, J. Bechtel, T. Berger, E. Butz, R. Caspart, T. Chwalek, W. De Boer, A. Dierlamm, A. Droll, K. El Morabit, N. Faltermann, K. Flöh, M. Giffels, J. o. Gosewisch, A. Gottmann, F. Hartmann, C. Heidecker, U. Husemann, I. Katkov, P. Keicher, R. Koppenhöfer, S. Maier, M. Metzler, S. Mitra, Th. Müller, M. Musich, M. Neukum, G. Quast, K. Rabbertz, J. Rauser, D. Savoiu, D. Schäfer, M. Schnepf, D. Seith, I. Shvetsov, H. J. Simonis, R. Ulrich, J. Van Der Linden, R. F. Von Cube, M. Wassmer, M. Weber, S. Wieland, R. Wolf, S. Wozniewski, S. Wunsch, G. Anagnostou, P. Asenov, G. Daskalakis, T. Geralis, A. Kyriakis, D. Loukas, G. Paspalaki, A. Stakia, M. Diamantopoulou, D. Karasavvas, G. Karathanasis, P. Kontaxakis, C. K. Koraka, A. Manousakis-katsikakis, A. Panagiotou, I. Papavergou, N. Saoulidou, K. Theofilatos, E. Tziaferi, K. Vellidis, E. Vourliotis, G. Bakas, K. Kousouris, I. Papakrivopoulos, G. Tsipolitis, A. Zacharopoulou, I. Evangelou, C. Foudas, P. Gianneios, P. Katsoulis, P. Kokkas, N. Manthos, I. Papadopoulos, J. Strologas, M. Csanad, M. M. A. Gadallah, S. Lökös, P. Major, K. Mandal, A. Mehta, G. Pasztor, A. J. Rádl, O. Surányi, G.I. Veres, M. Bartók, G. Bencze, C. Hajdu, D. Horvath, F. Sikler, V. Veszpremi, G. Vesztergombi, S. Czellar, J. Karancsi, J. Molnar, Z. Szillasi, D. Teyssier, P. Raics, Z. L. Trocsanyi, B. Ujvari, T. Csorgo, F. Nemes, T. Novak, S. Choudhury, J. R. Komaragiri, D. Kumar, L. Panwar, P. C. Tiwari, S. Bahinipati, D. Dash, C. Kar, P. Mal, T. Mishra, V. K. Muraleedharan Nair Bindhu, A. Nayak, P. Saha, N. Sur, S. K. Swain, S. Bansal, S. B. Beri, V. Bhatnagar, G. Chaudhary, S. Chauhan, N. Dhingra, R. Gupta, A. Kaur, S. Kaur, P. Kumari, M. Meena, K. Sandeep, J. B. Singh, A. K. Virdi, A. Ahmed, A. Bhardwaj, B. C. Choudhary, R. B. Garg, M. Gola, S. Keshri, A. Kumar, M. Naimuddin, P. Priyanka, K. Ranjan, A. Shah, M. Bharti, R. Bhattacharya, S. Bhattacharya, D. Bhowmik, S. Dutta, S. Ghosh, B. Gomber, M. Maity, S. Nandan, P. Palit, P. K. Rout, G. Saha, B. Sahu, S. Sarkar, M. Sharan, B. Singh, S. Thakur, P. K. Behera, S. C. Behera, P. Kalbhor, A. Muhammad, R. Pradhan, P. R. Pujahari, A. Sharma, A. K. Sikdar, D. Dutta, V. Jha, V. Kumar, D. K. Mishra, K. Naskar, P. K. Netrakanti, L. M. Pant, P. Shukla, T. Aziz, S. Dugad, G. B. Mohanty, U. Sarkar, S. Banerjee, S. Bhattacharya, R. Chudasama, M. Guchait, S. Karmakar, S. Kumar, G. Majumder, K. Mazumdar, S. Mukherjee, D. Roy, S. Dube, B. Kansal, S. Pandey, A. Rane, A. Rastogi, S. Sharma, H. Bakhshiansohi, M. Zeinali, S. Chenarani, S. M. Etesami, M. Khakzad, M. Mohammadi Najafabadi, M. Felcini, M. Grunewald, M. Abbrescia, R. Aly, C. Aruta, A. Colaleo, D. Creanza, N. De Filippis, M. De Palma, A. Di Florio, A. Di Pilato, W. Elmetenawee, L. Fiore, A. Gelmi, M. Gul, G. Iaselli, M. Ince, S. Lezki, G. Maggi, M. Maggi, I. Margjeka, V. Mastrapasqua, J. A. Merlin, S. My, S. Nuzzo, A. Pompili, G. Pugliese, A. Ranieri, G. Selvaggi, L. Silvestris, F. M. Simone, R. Venditti, P. Verwilligen, G. Abbiendi, C. Battilana, D. Bonacorsi, L. Borgonovi, S. Braibant-Giacomelli, L. Brigliadori, R. Campanini, P. Capiluppi, A. Castro, F. R. Cavallo, C. Ciocca, M. Cuffiani, G. M. Dallavalle, T. Diotalevi, F. Fabbri, A. Fanfani, E. Fontanesi, P. Giacomelli, L. Giommi, C. Grandi, L. Guiducci, F. Iemmi, S. Lo Meo, S. Marcellini, G. Masetti, F. L. Navarria, A. Perrotta, F. Primavera, A. M. Rossi, T. Rovelli, G. P. Siroli, N. Tosi, S. Albergo, S. Costa, A. Di Mattia, R. Potenza, A. Tricomi, C. Tuve, G. Barbagli, A. Cassese, R. Ceccarelli, V. Ciulli, C. Civinini, R. D’Alessandro, F. Fiori, E. Focardi, G. Latino, P. Lenzi, M. Lizzo, M. Meschini, S. Paoletti, R. Seidita, G. Sguazzoni, L. Viliani, L. Benussi, S. Bianco, D. Piccolo, M. Bozzo, F. Ferro, R. Mulargia, E. Robutti, S. Tosi, A. Benaglia, F. Brivio, F. Cetorelli, V. Ciriolo, F. De Guio, M. E. Dinardo, P. Dini, S. Gennai, A. Ghezzi, P. Govoni, L. Guzzi, M. Malberti, S. Malvezzi, A. Massironi, D. Menasce, F. Monti, L. Moroni, M. Paganoni, D. Pedrini, S. Ragazzi, N. Redaelli, T. Tabarelli de Fatis, D. Valsecchi, D. Zuolo, S. Buontempo, N. Cavallo, A. De Iorio, F. Fabozzi, A. O. M. Iorio, L. Lista, S. Meola, P. Paolucci, B. Rossi, C. Sciacca, P. Azzi, N. Bacchetta, D. Bisello, P. Bortignon, A. Bragagnolo, R. Carlin, P. Checchia, P. De Castro Manzano, T. Dorigo, F. Gasparini, U. Gasparini, S. Y. Hoh, L. Layer, M. Margoni, A. T. Meneguzzo, M. Presilla, P. Ronchese, R. Rossin, F. Simonetto, G. Strong, M. Tosi, H. Yarar, M. Zanetti, P. Zotto, A. Zucchetta, G. Zumerle, C. Aime‘, A. Braghieri, S. Calzaferri, D. Fiorina, P. Montagna, S. P. Ratti, V. Re, M. Ressegotti, C. Riccardi, P. Salvini, I. Vai, P. Vitulo, G. M. Bilei, D. Ciangottini, L. Fanò, P. Lariccia, G. Mantovani, V. Mariani, M. Menichelli, F. Moscatelli, A. Piccinelli, A. Rossi, A. Santocchia, D. Spiga, T. Tedeschi, P. Azzurri, G. Bagliesi, V. Bertacchi, L. Bianchini, T. Boccali, E. Bossini, R. Castaldi, M. A. Ciocci, R. Dell’Orso, M. R. Di Domenico, S. Donato, A. Giassi, M. T. Grippo, F. Ligabue, E. Manca, G. Mandorli, A. Messineo, F. Palla, G. Ramirez-Sanchez, A. Rizzi, G. Rolandi, S. Roy Chowdhury, A. Scribano, N. Shafiei, P. Spagnolo, R. Tenchini, G. Tonelli, N. Turini, A. Venturi, P. G. Verdini, F. Cavallari, M. Cipriani, D. Del Re, E. Di Marco, M. Diemoz, E. Longo, P. Meridiani, G. Organtini, F. Pandolfi, R. Paramatti, C. Quaranta, S. Rahatlou, C. Rovelli, F. Santanastasio, L. Soffi, R. Tramontano, N. Amapane, R. Arcidiacono, S. Argiro, M. Arneodo, N. Bartosik, R. Bellan, A. Bellora, J. Berenguer Antequera, C. Biino, A. Cappati, N. Cartiglia, S. Cometti, M. Costa, R. Covarelli, N. Demaria, B. Kiani, F. Legger, C. Mariotti, S. Maselli, E. Migliore, V. Monaco, E. Monteil, M. Monteno, M. M. Obertino, G. Ortona, L. Pacher, N. Pastrone, M. Pelliccioni, G. L. Pinna Angioni, M. Ruspa, R. Salvatico, K. Shchelina, F. Siviero, V. Sola, A. Solano, D. Soldi, A. Staiano, M. Tornago, D. Trocino, S. Belforte, V. Candelise, M. Casarsa, F. Cossutti, A. Da Rold, G. Della Ricca, F. Vazzoler, S. Dogra, C. Huh, B. Kim, D. H. Kim, G. N. Kim, J. Lee, S. W. Lee, C. S. Moon, Y. D. Oh, S. I. Pak, B. C. Radburn-Smith, S. Sekmen, Y. C. Yang, H. Kim, D. H. Moon, B. Francois, T. J. Kim, J. Park, S. Cho, S. Choi, Y. Go, B. Hong, K. Lee, K. S. Lee, J. Lim, J. Park, S. K. Park, J. Yoo, J. Goh, A. Gurtu, H. S. Kim, Y. Kim, J. Almond, J. H. Bhyun, J. Choi, S. Jeon, J. Kim, J. S. Kim, S. Ko, H. Kwon, H. Lee, S. Lee, B. H. Oh, M. Oh, S. B. Oh, H. Seo, U. K. Yang, I. Yoon, D. Jeon, J. H. Kim, B. Ko, J. S. H. Lee, I. C. Park, Y. Roh, D. Song, I.J. Watson, S. Ha, H. D. Yoo, Y. Choi, Y. Jeong, H. Lee, Y. Lee, I. Yu, T. Beyrouthy, Y. Maghrbi, V. Veckalns, M. Ambrozas, A. Juodagalvis, A. Rinkevicius, G. Tamulaitis, A. Vaitkevicius, W. A. T. Wan Abdullah, M. N. Yusli, Z. Zolkapli, J. F. Benitez, A. Castaneda Hernandez, J. A. Murillo Quijada, L. Valencia Palomo, G. Ayala, H. Castilla-Valdez, E. De La Cruz-Burelo, I. Heredia-De La Cruz, R. Lopez-Fernandez, C. A. Mondragon Herrera, D. A. Perez Navarro, A. Sanchez-Hernandez, S. Carrillo Moreno, C. Oropeza Barrera, M. Ramirez-Garcia, F. Vazquez Valencia, I. Pedraza, H. A. Salazar Ibarguen, C. Uribe Estrada, J. Mijuskovic, N. Raicevic, D. Krofcheck, S. Bheesette, P. H. Butler, A. Ahmad, M. I. Asghar, A. Awais, M. I. M. Awan, H. R. Hoorani, W. A. Khan, S. Qazi, M. A. Shah, M. Shoaib, V. Avati, L. Grzanka, M. Malawski, H. Bialkowska, M. Bluj, B. Boimska, T. Frueboes, M. Górski, M. Kazana, M. Szleper, P. Traczyk, P. Zalewski, K. Bunkowski, K. Doroba, A. Kalinowski, M. Konecki, J. Krolikowski, M. Walczak, M. Araujo, P. Bargassa, D. Bastos, A. Boletti, P. Faccioli, M. Gallinaro, J. Hollar, N. Leonardo, T. Niknejad, J. Seixas, O. Toldaiev, J. Varela, S. Afanasiev, D. Budkouski, P. Bunin, M. Gavrilenko, I. Golutvin, I. Gorbunov, A. Kamenev, V. Karjavine, A. Lanev, A. Malakhov, V. Matveev, V. Palichik, V. Perelygin, M. Savina, D. Seitova, V. Shalaev, S. Shmatov, S. Shulha, V. Smirnov, O. Teryaev, N. Voytishin, A. Zarubin, I. Zhizhin, G. Gavrilov, V. Golovtcov, Y. Ivanov, V. Kim, E. Kuznetsova, V. Murzin, V. Oreshkin, I. Smirnov, D. Sosnov, V. Sulimov, L. Uvarov, S. Volkov, A. Vorobyev, Yu. Andreev, A. Dermenev, S. Gninenko, N. Golubev, A. Karneyeu, M. Kirsanov, N. Krasnikov, A. Pashenkov, G. Pivovarov, D. Tlisov, A. Toropin, V. Epshteyn, V. Gavrilov, N. Lychkovskaya, A. Nikitenko, V. Popov, G. Safronov, A. Spiridonov, A. Stepennov, M. Toms, E. Vlasov, A. Zhokin, T. Aushev, R. Chistov, M. Danilov, A. Oskin, P. Parygin, S. Polikarpov, V. Andreev, M. Azarkin, I. Dremin, M. Kirakosyan, A. Terkulov, A. Belyaev, E. Boos, V. Bunichev, M. Dubinin, L. Dudko, A. Gribushin, V. Klyukhin, O. Kodolova, I. Lokhtin, S. Obraztsov, M. Perfilov, S. Petrushanko, V. Savrin, V. Blinov, T. Dimova, L. Kardapoltsev, I. Ovtin, Y. Skovpen, I. Azhgirey, I. Bayshev, V. Kachanov, A. Kalinin, D. Konstantinov, V. Petrov, R. Ryutin, A. Sobol, S. Troshin, N. Tyurin, A. Uzunian, A. Volkov, A. Babaev, V. Okhotnikov, L. Sukhikh, V. Borchsh, V. Ivanchenko, E. Tcherniaev, P. Adzic, M. Dordevic, P. Milenovic, J. Milosevic, V. Milosevic, M. Aguilar-Benitez, J. Alcaraz Maestre, A. Álvarez Fernández, I. Bachiller, M. Barrio Luna, Cristina F. Bedoya, C. A. Carrillo Montoya, M. Cepeda, M. Cerrada, N. Colino, B. De La Cruz, A. Delgado Peris, J.P. Fernández Ramos, J. Flix, M. C. Fouz, O. Gonzalez Lopez, S. Goy Lopez, J.M. Hernandez, M. I. Josa, J. León Holgado, D. Moran, Á. Navarro Tobar, A. Pérez-Calero Yzquierdo, J. Puerta Pelayo, I. Redondo, L. Romero, S. Sánchez Navas, M. S. Soares, L. Urda Gómez, C. Willmott, J. F. de Trocóniz, R. Reyes-Almanza, B. Alvarez Gonzalez, J. Cuevas, C. Erice, J. Fernandez Menendez, S. Folgueras, I. Gonzalez Caballero, E. Palencia Cortezon, C. Ramón Álvarez, J. Ripoll Sau, V. Rodríguez Bouza, A. Trapote, J. A. Brochero Cifuentes, I. J. Cabrillo, A. Calderon, B. Chazin Quero, J. Duarte Campderros, M. Fernandez, C. Fernandez Madrazo, P.J. Fernández Manteca, A. García Alonso, G. Gomez, C. Martinez Rivero, P. Martinez Ruiz del Arbol, F. Matorras, J. Piedra Gomez, C. Prieels, F. Ricci-Tam, T. Rodrigo, A. Ruiz-Jimeno, L. Scodellaro, N. Trevisani, I. Vila, J. M. Vizan Garcia, MK Jayananda, B. Kailasapathy, D. U. J. Sonnadara, DDC Wickramarathna, W. G. D. Dharmaratna, K. Liyanage, N. Perera, N. Wickramage, T. K. Aarrestad, D. Abbaneo, J. Alimena, E. Auffray, G. Auzinger, J. Baechler, P. Baillon, A. H. Ball, D. Barney, J. Bendavid, N. Beni, M. Bianco, A. Bocci, E. Brondolin, T. Camporesi, M. Capeans Garrido, G. Cerminara, S. S. Chhibra, L. Cristella, D. d’Enterria, A. Dabrowski, N. Daci, A. David, A. De Roeck, M. Deile, R. Di Maria, M. Dobson, M. Dünser, N. Dupont, A. Elliott-Peisert, N. Emriskova, F. Fallavollita, D. Fasanella, S. Fiorendi, A. Florent, G. Franzoni, J. Fulcher, W. Funk, S. Giani, D. Gigi, K. Gill, F. Glege, L. Gouskos, M. Haranko, J. Hegeman, Y. Iiyama, V. Innocente, T. James, P. Janot, J. Kaspar, J. Kieseler, M. Komm, N. Kratochwil, C. Lange, S. Laurila, P. Lecoq, K. Long, C. Lourenço, L. Malgeri, S. Mallios, M. Mannelli, F. Meijers, S. Mersi, E. Meschi, F. Moortgat, M. Mulders, S. Orfanelli, L. Orsini, F. Pantaleo, L. Pape, E. Perez, M. Peruzzi, A. Petrilli, G. Petrucciani, A. Pfeiffer, M. Pierini, M. Pitt, H. Qu, T. Quast, D. Rabady, A. Racz, M. Rieger, M. Rovere, H. Sakulin, J. Salfeld-Nebgen, S. Scarfi, C. Schäfer, C. Schwick, M. Selvaggi, A. Sharma, P. Silva, W. Snoeys, P. Sphicas, S. Summers, V. R. Tavolaro, D. Treille, A. Tsirou, G.P. Van Onsem, M. Verzetti, K. A. Wozniak, W. D. Zeuner, L. Caminada, A. Ebrahimi, W. Erdmann, R. Horisberger, Q. Ingram, H. C. Kaestli, D. Kotlinski, U. Langenegger, M. Missiroli, T. Rohe, K. Androsov, M. Backhaus, P. Berger, A. Calandri, N. Chernyavskaya, A. De Cosa, G. Dissertori, M. Dittmar, M. Donegà, C. Dorfer, T. Gadek, T. A. Gómez Espinosa, C. Grab, D. Hits, W. Lustermann, A.-M. Lyon, R. A. Manzoni, M. T. Meinhard, F. Micheli, F. Nessi-Tedaldi, J. Niedziela, F. Pauss, V. Perovic, G. Perrin, S. Pigazzini, M. G. Ratti, M. Reichmann, C. Reissel, T. Reitenspiess, B. Ristic, D. Ruini, D. A. Sanz Becerra, M. Schönenberger, V. Stampf, J. Steggemann, R. Wallny, D. H. Zhu, C. Amsler, C. Botta, D. Brzhechko, M. F. Canelli, A. De Wit, R. Del Burgo, J.K. Heikkilä, M. Huwiler, A. Jofrehei, B. Kilminster, S. Leontsinis, A. Macchiolo, P. Meiring, V. M. Mikuni, U. Molinatti, I. Neutelings, G. Rauco, A. Reimers, P. Robmann, S. Sanchez Cruz, K. Schweiger, Y. Takahashi, C. Adloff, C. M. Kuo, W. Lin, A. Roy, T. Sarkar, S. S. Yu, L. Ceard, P. Chang, Y. Chao, K. F. Chen, P. H. Chen, W.-S. Hou, Y. y. Li, R.-S. Lu, E. Paganis, A. Psallidas, A. Steen, E. Yazgan, P. r. Yu, B. Asavapibhop, C. Asawatangtrakuldee, N. Srimanobhas, F. Boran, S. Damarseckin, Z. S. Demiroglu, F. Dolek, I. Dumanoglu, E. Eskut, G. Gokbulut, Y. Guler, E. Gurpinar Guler, I. Hos, C. Isik, E. E. Kangal, O. Kara, A. Kayis Topaksu, U. Kiminsu, G. Onengut, K. Ozdemir, A. Polatoz, A. E. Simsek, B. Tali, U. G. Tok, S. Turkcapar, I. S. Zorbakir, C. Zorbilmez, B. Isildak, G. Karapinar, K. Ocalan, M. Yalvac, B. Akgun, I. O. Atakisi, E. Gülmez, M. Kaya, O. Kaya, Ö. Özçelik, S. Tekten, E. A. Yetkin, A. Cakir, K. Cankocak, Y. Komurcu, S. Sen, F. Aydogmus Sen, S. Cerci, B. Kaynak, S. Ozkorucuklu, D. Sunar Cerci, B. Grynyov, L. Levchuk, E. Bhal, S. Bologna, J. J. Brooke, A. Bundock, E. Clement, D. Cussans, H. Flacher, J. Goldstein, G. P. Heath, H. F. Heath, L. Kreczko, B. Krikler, S. Paramesvaran, T. Sakuma, S. Seif El Nasr-Storey, V. J. Smith, N. Stylianou, J. Taylor, A. Titterton, K. W. Bell, A. Belyaev, C. Brew, R. M. Brown, D. J. A. Cockerill, K. V. Ellis, K. Harder, S. Harper, J. Linacre, K. Manolopoulos, D. M. Newbold, E. Olaiya, D. Petyt, T. Reis, T. Schuh, C. H. Shepherd-Themistocleous, A. Thea, I. R. Tomalin, T. Williams, R. Bainbridge, P. Bloch, S. Bonomally, J. Borg, S. Breeze, O. Buchmuller, V. Cepaitis, G. S. Chahal, D. Colling, P. Dauncey, G. Davies, M. Della Negra, S. Fayer, G. Fedi, G. Hall, M. H. Hassanshahi, G. Iles, J. Langford, L. Lyons, A.-M. Magnan, S. Malik, A. Martelli, J. Nash, V. Palladino, M. Pesaresi, D. M. Raymond, A. Richards, A. Rose, E. Scott, C. Seez, A. Shtipliyski, A. Tapper, K. Uchida, T. Virdee, N. Wardle, S. N. Webb, D. Winterbottom, A. G. Zecchinelli, J. E. Cole, A. Khan, P. Kyberd, C. K. Mackay, I. D. Reid, L. Teodorescu, S. Zahid, S. Abdullin, A. Brinkerhoff, B. Caraway, J. Dittmann, K. Hatakeyama, A. R. Kanuganti, B. McMaster, N. Pastika, S. Sawant, C. Smith, C. Sutantawibul, J. Wilson, R. Bartek, A. Dominguez, R. Uniyal, A. M. Vargas Hernandez, A. Buccilli, O. Charaf, S. I. Cooper, D. Di Croce, S. V. Gleyzer, C. Henderson, C. U. Perez, P. Rumerio, C. West, A. Akpinar, A. Albert, D. Arcaro, C. Cosby, Z. Demiragli, D. Gastler, J. Rohlf, K. Salyer, D. Sperka, D. Spitzbart, I. Suarez, S. Yuan, D. Zou, G. Benelli, B. Burkle, X. Coubez, D. Cutts, Y. t. Duh, M. Hadley, U. Heintz, J. M. Hogan, K. H. M. Kwok, E. Laird, G. Landsberg, K. T. Lau, J. Lee, J. Luo, M. Narain, S. Sagir, E. Usai, W. Y. Wong, X. Yan, D. Yu, W. Zhang, C. Brainerd, R. Breedon, M. Calderon De La Barca Sanchez, M. Chertok, J. Conway, P. T. Cox, R. Erbacher, F. Jensen, O. Kukral, R. Lander, M. Mulhearn, D. Pellett, D. Taylor, M. Tripathi, Y. Yao, F. Zhang, M. Bachtis, R. Cousins, A. Dasgupta, A. Datta, D. Hamilton, J. Hauser, M. Ignatenko, M. A. Iqbal, T. Lam, N. Mccoll, W. A. Nash, S. Regnard, D. Saltzberg, C. Schnaible, B. Stone, V. Valuev, K. Burt, Y. Chen, R. Clare, J. W. Gary, G. Hanson, G. Karapostoli, O. R. Long, N. Manganelli, M. Olmedo Negrete, W. Si, S. Wimpenny, Y. Zhang, J. G. Branson, P. Chang, S. Cittolin, S. Cooperstein, N. Deelen, J. Duarte, R. Gerosa, L. Giannini, D. Gilbert, J. Guiang, V. Krutelyov, R. Lee, J. Letts, M. Masciovecchio, S. May, S. Padhi, M. Pieri, B. V. Sathia Narayanan, V. Sharma, M. Tadel, A. Vartak, F. Würthwein, Y. Xiang, A. Yagil, N. Amin, C. Campagnari, M. Citron, A. Dorsett, V. Dutta, J. Incandela, M. Kilpatrick, B. Marsh, H. Mei, A. Ovcharova, M. Quinnan, J. Richman, U. Sarica, D. Stuart, S. Wang, A. Bornheim, O. Cerri, I. Dutta, J. M. Lawhorn, N. Lu, J. Mao, H. B. Newman, J. Ngadiuba, T. Q. Nguyen, M. Spiropulu, J. R. Vlimant, C. Wang, S. Xie, Z. Zhang, R. Y. Zhu, J. Alison, M. B. Andrews, T. Ferguson, T. Mudholkar, M. Paulini, I. Vorobiev, J. P. Cumalat, W. T. Ford, E. MacDonald, R. Patel, A. Perloff, K. Stenson, K. A. Ulmer, S. R. Wagner, J. Alexander, Y. Cheng, J. Chu, D. J. Cranshaw, K. Mcdermott, J. Monroy, J. R. Patterson, D. Quach, A. Ryd, W. Sun, S. M. Tan, Z. Tao, J. Thom, P. Wittich, M. Zientek, M. Albrow, M. Alyari, G. Apollinari, A. Apresyan, A. Apyan, S. Banerjee, L. A. T. Bauerdick, A. Beretvas, D. Berry, J. Berryhill, P. C. Bhat, K. Burkett, J. N. Butler, A. Canepa, G. B. Cerati, H. W. K. Cheung, F. Chlebana, M. Cremonesi, K. F. Di Petrillo, V. D. Elvira, J. Freeman, Z. Gecse, L. Gray, D. Green, S. Grünendahl, O. Gutsche, R. M. Harris, R. Heller, T. C. Herwig, J. Hirschauer, B. Jayatilaka, S. Jindariani, M. Johnson, U. Joshi, P. Klabbers, T. Klijnsma, B. Klima, M. J. Kortelainen, S. Lammel, D. Lincoln, R. Lipton, T. Liu, J. Lykken, C. Madrid, K. Maeshima, C. Mantilla, D. Mason, P. McBride, P. Merkel, S. Mrenna, S. Nahn, V. O’Dell, V. Papadimitriou, K. Pedro, C. Pena, O. Prokofyev, F. Ravera, A. Reinsvold Hall, L. Ristori, B. Schneider, E. Sexton-Kennedy, N. Smith, A. Soha, L. Spiegel, S. Stoynev, J. Strait, L. Taylor, S. Tkaczyk, N. V. Tran, L. Uplegger, E. W. Vaandering, H. A. Weber, A. Woodard, D. Acosta, P. Avery, D. Bourilkov, L. Cadamuro, V. Cherepanov, F. Errico, R. D. Field, D. Guerrero, B. M. Joshi, M. Kim, J. Konigsberg, A. Korytov, K. H. Lo, K. Matchev, N. Menendez, G. Mitselmakher, D. Rosenzweig, K. Shi, J. Sturdy, J. Wang, E. Yigitbasi, X. Zuo, T. Adams, A. Askew, D. Diaz, R. Habibullah, S. Hagopian, V. Hagopian, K. F. Johnson, R. Khurana, T. Kolberg, G. Martinez, H. Prosper, C. Schiber, R. Yohay, J. Zhang, M. M. Baarmand, S. Butalla, T. Elkafrawy, M. Hohlmann, R. Kumar Verma, D. Noonan, M. Rahmani, M. Saunders, F. Yumiceva, M. R. Adams, L. Apanasevich, H. Becerril Gonzalez, R. Cavanaugh, X. Chen, S. Dittmer, O. Evdokimov, C.E. Gerber, D. A. Hangal, D. J. Hofman, C. Mills, G. Oh, T. Roy, M. B. Tonjes, N. Varelas, J. Viinikainen, X. Wang, Z. Wu, Z. Ye, M. Alhusseini, K. Dilsiz, S. Durgut, R. P. Gandrajula, M. Haytmyradov, V. Khristenko, O. K. Köseyan, J.-P. Merlo, A. Mestvirishvili, A. Moeller, J. Nachtman, H. Ogul, Y. Onel, F. Ozok, A. Penzo, C. Snyder, E. Tiras, J. Wetzel, O. Amram, B. Blumenfeld, L. Corcodilos, J. Davis, M. Eminizer, A. V. Gritsan, S. Kyriacou, P. Maksimovic, J. Roskes, M. Swartz, T. Á. Vámi, C. Baldenegro Barrera, P. Baringer, A. Bean, A. Bylinkin, T. Isidori, S. Khalil, J. King, G. Krintiras, A. Kropivnitskaya, C. Lindsey, N. Minafra, M. Murray, C. Rogan, C. Royon, S. Sanders, E. Schmitz, J. D. Tapia Takaki, Q. Wang, J. Williams, G. Wilson, S. Duric, A. Ivanov, K. Kaadze, D. Kim, Y. Maravin, T. Mitchell, A. Modak, K. Nam, F. Rebassoo, D. Wright, E. Adams, A. Baden, O. Baron, A. Belloni, S. C. Eno, Y. Feng, N. J. Hadley, S. Jabeen, R. G. Kellogg, T. Koeth, A. C. Mignerey, S. Nabili, M. Seidel, A. Skuja, S. C. Tonwar, L. Wang, K. Wong, D. Abercrombie, R. Bi, S. Brandt, W. Busza, I. A. Cali, Y. Chen, M. D’Alfonso, G. Gomez Ceballos, M. Goncharov, P. Harris, M. Hu, M. Klute, D. Kovalskyi, J. Krupa, Y.-J. Lee, B. Maier, A. C. Marini, C. Mironov, C. Paus, D. Rankin, C. Roland, G. Roland, Z. Shi, G. S. F. Stephans, K. Tatar, J. Wang, Z. Wang, B. Wyslouch, R. M. Chatterjee, A. Evans, P. Hansen, J. Hiltbrand, Sh. Jain, M. Krohn, Y. Kubota, Z. Lesko, J. Mans, M. Revering, R. Rusack, R. Saradhy, N. Schroeder, N. Strobbe, M. A. Wadud, J. G. Acosta, S. Oliveros, K. Bloom, M. Bryson, S. Chauhan, D. R. Claes, C. Fangmeier, L. Finco, F. Golf, J. R. González Fernández, C. Joo, I. Kravchenko, J. E. Siado, G. R. Snow, W. Tabb, F. Yan, G. Agarwal, H. Bandyopadhyay, L. Hay, I. Iashvili, A. Kharchilava, C. McLean, D. Nguyen, J. Pekkanen, S. Rappoccio, A. Williams, G. Alverson, E. Barberis, C. Freer, Y. Haddad, A. Hortiangtham, J. Li, G. Madigan, B. Marzocchi, D. M. Morse, V. Nguyen, T. Orimoto, A. Parker, L. Skinnari, A. Tishelman-Charny, T. Wamorkar, B. Wang, A. Wisecarver, D. Wood, S. Bhattacharya, J. Bueghly, Z. Chen, A. Gilbert, T. Gunter, K. A. Hahn, N. Odell, M. H. Schmitt, K. Sung, M. Velasco, R. Band, R. Bucci, N. Dev, R. Goldouzian, M. Hildreth, K. Hurtado Anampa, C. Jessop, K. Lannon, N. Loukas, N. Marinelli, I. Mcalister, F. Meng, K. Mohrman, Y. Musienko, R. Ruchti, P. Siddireddy, M. Wayne, A. Wightman, M. Wolf, M. Zarucki, L. Zygala, B. Bylsma, B. Cardwell, L. S. Durkin, B. Francis, C. Hill, A. Lefeld, B. L. Winer, B. R. Yates, F. M. Addesa, B. Bonham, P. Das, G. Dezoort, P. Elmer, A. Frankenthal, B. Greenberg, N. Haubrich, S. Higginbotham, A. Kalogeropoulos, G. Kopp, S. Kwan, D. Lange, M. T. Lucchini, D. Marlow, K. Mei, I. Ojalvo, J. Olsen, C. Palmer, D. Stickland, C. Tully, S. Malik, S. Norberg, A. S. Bakshi, V. E. Barnes, R. Chawla, S. Das, L. Gutay, M. Jones, A. W. Jung, S. Karmarkar, M. Liu, G. Negro, N. Neumeister, C. C. Peng, S. Piperov, A. Purohit, J. F. Schulte, M. Stojanovic, J. Thieman, F. Wang, R. Xiao, W. Xie, J. Dolen, N. Parashar, A. Baty, S. Dildick, K. M. Ecklund, S. Freed, F. J. M. Geurts, A. Kumar, W. Li, B. P. Padley, R. Redjimi, J. Roberts, W. Shi, A. G. Stahl Leiton, A. Bodek, P. de Barbaro, R. Demina, J. L. Dulemba, C. Fallon, T. Ferbel, M. Galanti, A. Garcia-Bellido, O. Hindrichs, A. Khukhunaishvili, E. Ranken, R. Taus, B. Chiarito, J. P. Chou, A. Gandrakota, Y. Gershtein, E. Halkiadakis, A. Hart, M. Heindl, E. Hughes, S. Kaplan, O. Karacheban, I. Laflotte, A. Lath, R. Montalvo, K. Nash, M. Osherson, S. Salur, S. Schnetzer, S. Somalwar, R. Stone, S. A. Thayil, S. Thomas, H. Wang, H. Acharya, A. G. Delannoy, S. Spanier, O. Bouhali, M. Dalchenko, A. Delgado, R. Eusebi, J. Gilmore, T. Huang, T. Kamon, H. Kim, S. Luo, S. Malhotra, R. Mueller, D. Overton, D. Rathjens, A. Safonov, N. Akchurin, J. Damgov, V. Hegde, S. Kunori, K. Lamichhane, S. W. Lee, T. Mengke, S. Muthumuni, T. Peltola, S. Undleeb, I. Volobouev, Z. Wang, A. Whitbeck, E. Appelt, S. Greene, A. Gurrola, W. Johns, C. Maguire, A. Melo, H. Ni, K. Padeken, F. Romeo, P. Sheldon, S. Tuo, J. Velkovska, M. W. Arenton, B. Cox, G. Cummings, J. Hakala, R. Hirosky, M. Joyce, A. Ledovskoy, A. Li, C. Neu, B. Tannenwald, E. Wolfe, P. E. Karchin, N. Poudyal, P. Thapa, K. Black, T. Bose, J. Buchanan, C. Caillol, S. Dasu, I. De Bruyn, P. Everaerts, F. Fienga, C. Galloni, H. He, M. Herndon, A. Hervé, U. Hussain, A. Lanaro, A. Loeliger, R. Loveless, J. Madhusudanan Sreekala, A. Mallampalli, A. Mohammadi, D. Pinna, A. Savin, V. Shang, V. Sharma, W. H. Smith, D. Teague, S. Trembath-reichert, W. Vetens

**Affiliations:** 1 Yerevan Physics Institute, Yerevan, Armenia; 2 Institut für Hochenergiephysik, Wien, Austria; 3 Institute for Nuclear Problems, Minsk, Belarus; 4 Universiteit Antwerpen, Antwerpen, Belgium; 5 Vrije Universiteit Brussel, Brussel, Belgium; 6 Université Libre de Bruxelles, Bruxelles, Belgium; 7 Ghent University, Ghent, Belgium; 8 Université Catholique de Louvain, Louvain-la-Neuve, Belgium; 9 Centro Brasileiro de Pesquisas Fisicas, Rio de Janeiro, Brazil; 10 Universidade do Estado do Rio de Janeiro, Rio de Janeiro, Brazil; 11 Universidade Estadual Paulista, Universidade Federal do ABC, São Paulo, Brazil; 12 Institute for Nuclear Research and Nuclear Energy, Bulgarian Academy of Sciences, Sofia, Bulgaria; 13 University of Sofia, Sofia, Bulgaria; 14 Beihang University, Beijing, China; 15 Department of Physics, Tsinghua University, Beijing, China; 16 Institute of High Energy Physics, Beijing, China; 17 State Key Laboratory of Nuclear Physics and Technology, Peking University, Beijing, China; 18 Sun Yat-Sen University, Guangzhou, China; 19 Institute of Modern Physics and Key Laboratory of Nuclear Physics and Ion-beam Application (MOE), Fudan University, Shanghai, China; 20 Zhejiang University, Hangzhou, China; 21 Universidad de Los Andes, Bogota, Colombia; 22 Universidad de Antioquia, Medellin, Colombia; 23 Faculty of Electrical Engineering, Mechanical Engineering and Naval Architecture, University of Split, Split, Croatia; 24 Faculty of Science, University of Split, Split, Croatia; 25 Institute Rudjer Boskovic, Zagreb, Croatia; 26 University of Cyprus, Nicosia, Cyprus; 27 Charles University, Prague, Czech Republic; 28 Escuela Politecnica Nacional, Quito, Ecuador; 29 Universidad San Francisco de Quito, Quito, Ecuador; 30 Academy of Scientific Research and Technology of the Arab Republic of Egypt, Egyptian Network of High Energy Physics, Cairo, Egypt; 31 Center for High Energy Physics (CHEP-FU), Fayoum University, El-Fayoum, Egypt; 32 National Institute of Chemical Physics and Biophysics, Tallinn, Estonia; 33 Department of Physics, University of Helsinki, Helsinki, Finland; 34 Helsinki Institute of Physics, Helsinki, Finland; 35 Lappeenranta University of Technology, Lappeenranta, Finland; 36 IRFU, CEA, Université Paris-Saclay, Gif-sur-Yvette, France; 37 Laboratoire Leprince-Ringuet, CNRS/IN2P3, Ecole Polytechnique, Institut Polytechnique de Paris, Palaiseau, France; 38 Université de Strasbourg, CNRS, IPHC UMR 7178, Strasbourg, France; 39 Institut de Physique des 2 Infinis de Lyon (IP2I ), Villeurbanne, France; 40 Georgian Technical University, Tbilisi, Georgia; 41 I. Physikalisches Institut, RWTH Aachen University, Aachen, Germany; 42 III. Physikalisches Institut A, RWTH Aachen University, Aachen, Germany; 43 III. Physikalisches Institut B, RWTH Aachen University, Aachen, Germany; 44 Deutsches Elektronen-Synchrotron, Hamburg, Germany; 45 University of Hamburg, Hamburg, Germany; 46 Karlsruher Institut fuer Technologie, Karlsruhe, Germany; 47 Institute of Nuclear and Particle Physics (INPP), NCSR Demokritos, Aghia Paraskevi, Greece; 48 National and Kapodistrian University of Athens, Athens, Greece; 49 National Technical University of Athens, Athens, Greece; 50 University of Ioánnina, Ioannina, Greece; 51 MTA-ELTE Lendület CMS Particle and Nuclear Physics Group, Eötvös Loránd University, Budapest, Hungary; 52 Wigner Research Centre for Physics, Budapest, Hungary; 53 Institute of Nuclear Research ATOMKI, Debrecen, Hungary; 54 Institute of Physics, University of Debrecen, Debrecen, Hungary; 55 Eszterhazy Karoly University, Karoly Robert Campus, Gyongyos, Hungary; 56 Indian Institute of Science (IISc), Bangalore, India; 57 National Institute of Science Education and Research, HBNI, Bhubaneswar, India; 58 Panjab University, Chandigarh, India; 59 University of Delhi, Delhi, India; 60 Saha Institute of Nuclear Physics, HBNI, Kolkata, India; 61 Indian Institute of Technology Madras, Madras, India; 62 Bhabha Atomic Research Centre, Mumbai, India; 63 Tata Institute of Fundamental Research-A, Mumbai, India; 64 Tata Institute of Fundamental Research-B, Mumbai, India; 65 Indian Institute of Science Education and Research (IISER), Pune, India; 66 Department of Physics, Isfahan University of Technology, Isfahan, Iran; 67 Institute for Research in Fundamental Sciences (IPM), Tehran, Iran; 68 University College Dublin, Dublin, Ireland; 69 INFN Sezione di Bari , Università di Bari, Politecnico di Bari, Bari, Italy; 70 INFN Sezione di Bologna, Università di Bologna, Bologna, Italy; 71 INFN Sezione di Catania, Università di Catania, Catania, Italy; 72 INFN Sezione di Firenze, Università di Firenze, Firenze, Italy; 73 INFN Laboratori Nazionali di Frascati, Frascati, Italy; 74 INFN Sezione di Genova, Università di Genova, Genoa, Italy; 75 INFN Sezione di Milano-Bicocca, Università di Milano-Bicocca, Milan, Italy; 76 INFN Sezione di Napoli , Università di Napoli ’Federico II’ , Napoli, Italy, Università della Basilicata , Potenza, Italy, Università G. Marconi, Rome, Italy; 77 INFN Sezione di Padova , Università di Padova , Padova, Italy, Università di Trento, Trento, Italy; 78 INFN Sezione di Pavia, Università di Pavia, Pavia, Italy; 79 INFN Sezione di Perugia, Università di Perugia, Perugia, Italy; 80 INFN Sezione di Pisa , Università di Pisa , Scuola Normale Superiore di Pisa , Pisa Italy, Università di Siena, Siena, Italy; 81 INFN Sezione di Roma, Sapienza Università di Roma, Rome, Italy; 82 INFN Sezione di Torino , Università di Torino , Torino, Italy, Università del Piemonte Orientale, Novara, Italy; 83 INFN Sezione di Trieste, Università di Trieste, Trieste, Italy; 84 Kyungpook National University, Daegu, Korea; 85 Chonnam National University, Institute for Universe and Elementary Particles, Kwangju, Korea; 86 Hanyang University, Seoul, Korea; 87 Korea University, Seoul, Korea; 88 Kyung Hee University, Department of Physics, Seoul, Republic of Korea; 89 Sejong University, Seoul, Korea; 90 Seoul National University, Seoul, Korea; 91 University of Seoul, Seoul, Korea; 92 Department of Physics, Yonsei University, Seoul, Korea; 93 Sungkyunkwan University, Suwon, Korea; 94 College of Engineering and Technology, American University of the Middle East (AUM), Egaila, Kuwait; 95 Riga Technical University, Riga, Latvia; 96 Vilnius University, Vilnius, Lithuania; 97 National Centre for Particle Physics, Universiti Malaya, Kuala Lumpur, Malaysia; 98 Universidad de Sonora (UNISON), Hermosillo, Mexico; 99 Centro de Investigacion y de Estudios Avanzados del IPN, Mexico City, Mexico; 100 Universidad Iberoamericana, Mexico City, Mexico; 101 Benemerita Universidad Autonoma de Puebla, Puebla, Mexico; 102 University of Montenegro, Podgorica, Montenegro; 103 University of Auckland, Auckland, New Zealand; 104 University of Canterbury, Christchurch, New Zealand; 105 National Centre for Physics, Quaid-I-Azam University, Islamabad, Pakistan; 106 Electronics and Telecommunications, AGH University of Science and Technology Faculty of Computer Science, Kraków, Poland; 107 National Centre for Nuclear Research, Swierk, Poland; 108 Institute of Experimental Physics, Faculty of Physics, University of Warsaw, Warsaw, Poland; 109 Laboratório de Instrumentação e Física Experimental de Partículas, Lisbon, Portugal; 110 Joint Institute for Nuclear Research, Dubna, Russia; 111 Petersburg Nuclear Physics Institute, Gatchina (St. Petersburg), Russia; 112 Institute for Nuclear Research, Moscow, Russia; 113 Institute for Theoretical and Experimental Physics named by A.I. Alikhanov of NRC ‘Kurchatov Institute’, Moscow, Russia; 114 Moscow Institute of Physics and Technology, Moscow, Russia; 115 National Research Nuclear University ’Moscow Engineering Physics Institute’ (MEPhI), Moscow, Russia; 116 P.N. Lebedev Physical Institute, Moscow, Russia; 117 Skobeltsyn Institute of Nuclear Physics, Lomonosov Moscow State University, Moscow, Russia; 118 Novosibirsk State University (NSU), Novosibirsk, Russia; 119 Institute for High Energy Physics of National Research Centre ‘Kurchatov Institute’, Protvino, Russia; 120 National Research Tomsk Polytechnic University, Tomsk, Russia; 121 Tomsk State University, Tomsk, Russia; 122 Faculty of Physics and VINCA Institute of Nuclear Sciences, University of Belgrade, Belgrade, Serbia; 123 Centro de Investigaciones Energéticas Medioambientales y Tecnológicas (CIEMAT), Madrid, Spain; 124 Universidad Autónoma de Madrid, Madrid, Spain; 125 Instituto Universitario de Ciencias y Tecnologías Espaciales de Asturias (ICTEA), Universidad de Oviedo, Oviedo, Spain; 126 Instituto de Física de Cantabria (IFCA), CSIC-Universidad de Cantabria, Santander, Spain; 127 University of Colombo, Colombo, Sri Lanka; 128 Department of Physics, University of Ruhuna, Matara, Sri Lanka; 129 CERN, European Organization for Nuclear Research, Geneva, Switzerland; 130 Paul Scherrer Institut, Villigen, Switzerland; 131 ETH Zurich-Institute for Particle Physics and Astrophysics (IPA), Zurich, Switzerland; 132 Universität Zürich, Zurich, Switzerland; 133 National Central University, Chung-Li, Taiwan; 134 National Taiwan University (NTU), Taipei, Taiwan; 135 Department of Physics, Faculty of Science, Chulalongkorn University, Bangkok, Thailand; 136 Physics Department, Science and Art Faculty, Çukurova University, Adana, Turkey; 137 Physics Department, Middle East Technical University, Ankara, Turkey; 138 Bogazici University, Istanbul, Turkey; 139 Istanbul Technical University, Istanbul, Turkey; 140 Istanbul University, Istanbul, Turkey; 141 Institute for Scintillation Materials of National Academy of Science of Ukraine, Kharkiv, Ukraine; 142 National Scientific Center, Kharkov Institute of Physics and Technology, Kharkiv, Ukraine; 143 University of Bristol, Bristol, UK; 144 Rutherford Appleton Laboratory, Didcot, UK; 145 Imperial College, London, UK; 146 Brunel University, Uxbridge, UK; 147 Baylor University, Waco, USA; 148 Catholic University of America, Washington, DC USA; 149 The University of Alabama, Tuscaloosa, USA; 150 Boston University, Boston, USA; 151 Brown University, Providence, USA; 152 University of California, Davis, Davis, USA; 153 University of California, Los Angeles, USA; 154 University of California, Riverside, Riverside, USA; 155 University of California, San Diego, La Jolla, USA; 156 Department of Physics, University of California, Santa Barbara, Santa Barbara, USA; 157 California Institute of Technology, Pasadena, USA; 158 Carnegie Mellon University, Pittsburgh, USA; 159 University of Colorado Boulder, Boulder, USA; 160 Cornell University, Ithaca, USA; 161 Fermi National Accelerator Laboratory, Batavia, USA; 162 University of Florida, Gainesville, USA; 163 Florida State University, Tallahassee, USA; 164 Florida Institute of Technology, Melbourne, USA; 165 University of Illinois at Chicago (UIC), Chicago, USA; 166 The University of Iowa, Iowa City, USA; 167 Johns Hopkins University, Baltimore, USA; 168 The University of Kansas, Lawrence, USA; 169 Kansas State University, Manhattan, USA; 170 Lawrence Livermore National Laboratory, Livermore, USA; 171 University of Maryland, College Park, USA; 172 Massachusetts Institute of Technology, Cambridge, USA; 173 University of Minnesota, Minneapolis, USA; 174 University of Mississippi, Oxford, USA; 175 University of Nebraska-Lincoln, Lincoln, USA; 176 State University of New York at Buffalo, Buffalo, USA; 177 Northeastern University, Boston, USA; 178 Northwestern University, Evanston, USA; 179 University of Notre Dame, Notre Dame, USA; 180 The Ohio State University, Columbus, USA; 181 Princeton University, Princeton, USA; 182 University of Puerto Rico, Mayaguez, USA; 183 Purdue University, West Lafayette, USA; 184 Purdue University Northwest, Hammond, USA; 185 Rice University, Houston, USA; 186 University of Rochester, Rochester, USA; 187 Rutgers, The State University of New Jersey, Piscataway, USA; 188 University of Tennessee, Knoxville, USA; 189 Texas A&M University, College Station, USA; 190 Texas Tech University, Lubbock, USA; 191 Vanderbilt University, Nashville, USA; 192 University of Virginia, Charlottesville, USA; 193 Wayne State University, Detroit, USA; 194 University of Wisconsin - Madison, Madison, WI USA; 195 Vienna University of Technology, Vienna, Austria; 196 Institute of Basic and Applied Sciences, Faculty of Engineering, Arab Academy for Science, Technology and Maritime Transport, Alexandria Egypt, Alexandria, Egypt; 197 Université Libre de Bruxelles, Bruxelles, Belgium; 198 Universidade Estadual de Campinas, Campinas, Brazil; 199 Federal University of Rio Grande do Sul, Porto Alegre, Brazil; 200 UFMS, Nova Andradina, Brazil; 201 University of Chinese Academy of Sciences, Beijing, China; 202 Department of Physics, Tsinghua University, Beijing, China, Beijing, China; 203 Nanjing Normal University Department of Physics, Nanjing, China; 204 The University of Iowa, Iowa City, USA; 205 Institute for Theoretical and Experimental Physics named by A.I. Alikhanov of NRC ‘Kurchatov Institute’, Moscow, Russia; 206 Joint Institute for Nuclear Research, Dubna, Russia; 207 Ain Shams University, Cairo, Egypt; 208 Zewail City of Science and Technology, Zewail, Egypt; 209 British University in Egypt, Cairo, Egypt; 210 Purdue University, West Lafayette, USA; 211 Université de Haute Alsace, Mulhouse, France; 212 Erzincan Binali Yildirim University, Erzincan, Turkey; 213 CERN European Organization for Nuclear Research, Geneva, Switzerland; 214 III. Physikalisches Institut A, RWTH Aachen University, Aachen, Germany; 215 University of Hamburg, Hamburg, Germany; 216 Department of Physics, Isfahan University of Technology, Isfahan, Iran; 217 Brandenburg University of Technology, Cottbus, Germany; 218 Skobeltsyn Institute of Nuclear Physics, Lomonosov Moscow State University, Moscow, Russia; 219 Physics Department, Faculty of Science, Assiut University, Assiut, Egypt; 220 Eszterhazy Karoly University, Karoly Robert Campus, Gyongyos, Hungary; 221 Institute of Physics, University of Debrecen, Debrecen, Hungary; 222 Institute of Nuclear Research ATOMKI, Debrecen, Hungary; 223 MTA-ELTE Lendület CMS Particle and Nuclear Physics Group, Eötvös Loránd University, Budapest, Hungary; 224 Wigner Research Centre for Physics, Budapest, Hungary; 225 IIT Bhubaneswar, Bhubaneswar, India; 226 Institute of Physics, Bhubaneswar, India; 227 G.H.G. Khalsa College, Punjab, India; 228 Shoolini University, Solan, India; 229 University of Hyderabad, Hyderabad, India; 230 University of Visva-Bharati, Santiniketan, India; 231 Indian Institute of Technology (IIT), Mumbai, India; 232 Deutsches Elektronen-Synchrotron, Hamburg, Germany; 233 Sharif University of Technology, Tehran, Iran; 234 Department of Physics, University of Science and Technology of Mazandaran, Behshahr, Iran; 235 INFN Sezione di Bari , Università di Bari, Politecnico di Bari, Bari, Italy; 236 Italian National Agency for New Technologies, Energy and Sustainable Economic Development, Bologna, Italy; 237 Centro Siciliano di Fisica Nucleare e di Struttura Della Materia, Catania, Italy; 238 Università di Napoli ’Federico II’, Naples, Italy; 239 Riga Technical University, Riga, Latvia; 240 Consejo Nacional de Ciencia y Tecnología, Mexico City, Mexico; 241 IRFU, CEA, Université Paris-Saclay, Gif-sur-Yvette, France; 242 Institute for Nuclear Research, Moscow, Russia; 243 National Research Nuclear University ‘Moscow Engineering Physics Institute’ (MEPhI), Moscow, Russia; 244 St. Petersburg State Polytechnical University, St. Petersburg, Russia; 245 University of Florida, Gainesville, USA; 246 Imperial College, London, UK; 247 Moscow Institute of Physics and Technology, Moscow, Russia; 248 P.N. Lebedev Physical Institute, Moscow, Russia; 249 California Institute of Technology, Pasadena, USA; 250 Budker Institute of Nuclear Physics, Novosibirsk, Russia; 251 Faculty of Physics, University of Belgrade, Belgrade, Serbia; 252 Trincomalee Campus, Eastern University, Sri Lanka, Nilaveli, Sri Lanka; 253 INFN Sezione di Pavia, Università di Pavia , Pavia, Italy, Pavia, Italy; 254 National and Kapodistrian University of Athens, Athens, Greece; 255 Universität Zürich, Zurich, Switzerland; 256 Ecole Polytechnique Fédérale Lausanne, Lausanne, Switzerland; 257 Stefan Meyer Institute for Subatomic Physics, Vienna, Austria; 258 Laboratoire d’Annecy-le-Vieux de Physique des Particules, IN2P3-CNRS, Annecy-le-Vieux, France; 259 Şırnak University, Sirnak, Turkey; 260 Near East University, Research Center of Experimental Health Science, Nicosia, Turkey; 261 Konya Technical University, Konya, Turkey; 262 Faculty of Engineering, Istanbul University-Cerraphasa, Istanbul, Turkey; 263 Mersin University, Mersin, Turkey; 264 Piri Reis University, Istanbul, Turkey; 265 Adiyaman University, Adiyaman, Turkey; 266 Ozyegin University, Istanbul, Turkey; 267 Izmir Institute of Technology, Izmir, Turkey; 268 Necmettin Erbakan University, Konya, Turkey; 269 Bozok Universitetesi Rektörlügü, Yozgat, Turkey, Yozgat, Turkey; 270 Marmara University, Istanbul, Turkey; 271 Milli Savunma University, Istanbul, Turkey; 272 Kafkas University, Kars, Turkey; 273 Istanbul Bilgi University, Istanbul, Turkey; 274 Hacettepe University, Ankara, Turkey; 275 Vrije Universiteit Brussel, Brussel, Belgium; 276 School of Physics and Astronomy, University of Southampton, Southampton, UK; 277 IPPP Durham University, Durham, UK; 278 Monash University, Faculty of Science, Clayton, Australia; 279 Bethel University, St. Paul, Minneapolis, USA; 280 Karamanoğlu Mehmetbey University, Karaman, Turkey; 281 Bingol University, Bingol, Turkey; 282 Georgian Technical University, Tbilisi, Georgia; 283 Sinop University, Sinop, Turkey; 284 Mimar Sinan University, Istanbul, Turkey; 285 Erciyes University, Kayseri, Turkey; 286 Texas A&M University at Qatar, Doha, Qatar; 287 Kyungpook National University, Daegu, Korea; 288 CERN, Geneva, Switzerland

## Abstract

Production cross sections of the Higgs boson are measured in the ${\mathrm{H}} \rightarrow {\mathrm{Z}} {\mathrm{Z}} \rightarrow 4\ell$ ($\ell ={\mathrm{e}},{{{\upmu }}_{\mathrm{}}^{\mathrm{}}}$) decay channel. A data sample of proton–proton collisions at a center-of-mass energy of 13$\,\text {TeV}$, collected by the CMS detector at the LHC and corresponding to an integrated luminosity of 137$\,\text {fb}^{-1}$ is used. The signal strength modifier $\mu$, defined as the ratio of the Higgs boson production rate in the $4\ell$ channel to the standard model (SM) expectation, is measured to be $\mu =0.94 \pm 0.07 \,\text {(stat)} ^{+0.09}_{-0.08} \,\text {(syst)}$ at a fixed value of $m_{{\mathrm{H}}} = 125.38\,\text {GeV}$. The signal strength modifiers for the individual Higgs boson production modes are also reported. The inclusive fiducial cross section for the ${\mathrm{H}} \rightarrow 4\ell$ process is measured to be $2.84^{+0.23}_{-0.22} \,\text {(stat)} ^{+0.26}_{-0.21} \,\text {(syst)} \,\text {fb}$, which is compatible with the SM prediction of $2.84 \pm 0.15 \,\text {fb}$ for the same fiducial region. Differential cross sections as a function of the transverse momentum and rapidity of the Higgs boson, the number of associated jets, and the transverse momentum of the leading associated jet are measured. A new set of cross section measurements in mutually exclusive categories targeted to identify production mechanisms and kinematical features of the events is presented. The results are in agreement with the SM predictions.

## Introduction

The discovery of the Higgs boson (H) in 2012 by the ATLAS and CMS collaborations [1–3] has been a major step towards the understanding of the electroweak symmetry breaking mechanism [4–9]. Further studies by the two experiments [10–13] have shown that the properties of the new particle are consistent with the standard model (SM) expectations for the H boson.

The ${\mathrm{H}} \rightarrow {\mathrm{Z}} {\mathrm{Z}} \rightarrow 4\ell$ decay channel ($\ell ={\mathrm{e}},{{{\upmu }}_{\mathrm{}}^{\mathrm{}}}$) has a large signal-to-background ratio thanks to a low background rate and the complete reconstruction of the final state decay products, capitalizing on the excellent lepton momentum resolution of the CMS detector. The measurements performed using this decay channel with the LHC Run 1 data set at center-of-mass energies of 7 and 8$\,\text {TeV}$, and the Run 2 data set at 13$\,\text {TeV}$ include the determination of the mass, the spin and the parity of the H boson [14–19], its width [20–23], the inclusive and differential fiducial cross sections [18, 24–28], and the tensor structure of the H boson interaction with a pair of neutral gauge bosons in both on-shell and off-shell regions [17, 19, 21, 29, 30].

This paper presents the measurement of production cross sections in granular kinematic regions of the H boson in the ${\mathrm{H}} \rightarrow {\mathrm{Z}} {\mathrm{Z}} \rightarrow 4\ell$ decay channel. A data sample of proton–proton (${\mathrm{p}} {\mathrm{p}}$) collisions at a center-of-mass energy of $\sqrt{s}=13\,\text {TeV}$, collected by the CMS detector at the LHC and corresponding to an integrated luminosity of $137{\,\text {fb}^{-1}}$ is used. The inclusive signal strength modifier, defined as the ratio of the H boson production rate in the $4\ell$ channel to the SM expectation, and signal strength modifiers for the individual H boson production modes are measured. The measurements of the inclusive and differential fiducial cross sections are also presented, and their compatibility with the SM predictions is tested. The present analysis is similar to that previously performed by the CMS Collaboration [18], but is based on a larger data sample.

In addition, measurements of the H boson cross sections within the simplified template cross section (STXS) framework [31–33] are also presented. The main goals of the STXS framework are to increase the reinterpretability of the precision H boson measurements and to minimize the theory dependence. This is achieved by defining exclusive kinematic regions in the H boson production phase space. The results presented within the STXS framework nonetheless depend on the SM simulation used to model the experimental acceptance of the signal processes, which could be modified in beyond the SM (BSM) scenarios. These kinematic regions, referred to as bins, are defined in different stages corresponding to increasing degrees of granularity. This paper presents results in the STXS stage 0 where the bins correspond closely to the different H boson production mechanisms. Previous measurements of cross sections in stage 0 production bins in the ${\mathrm{H}} \rightarrow 4\ell$ decay channel were already presented by the CMS Collaboration [18]. In the STXS framework, additional stages are defined by further splitting of the bins enhancing the sensitivity to possible signature of BSM physics at high transverse momentum of the H boson. Measurements of stage 0, stage 1, and stage 1.1 cross sections in the ${\mathrm{H}} \rightarrow 4\ell$ decay channel were recently published by the ATLAS Collaboration [27]. The most recent refinement of STXS binning is referred to as STXS stage 1.2. This paper presents a first set of the cross section measurements in the STXS stage 1.2 bins in the ${\mathrm{H}} \rightarrow 4\ell$ decay channel.

The paper is organized as follows. A brief introduction of the CMS detector is given in Sect. 2. The data, as well as the simulated signal and background samples, are described in Sect. 3. The event reconstruction and selection, the kinematic discriminants, and the categorization of the H boson candidate events are described in Sects. 4, 5, and 6 , respectively. The background estimation is detailed in Sect. 7 while the signal modeling is described in Sect. 8. The experimental and theoretical systematic uncertainties are described in Sect. 9 and the results are presented in Sect. 10. Concluding remarks are given in Sect. 11.

## The CMS detector

The central feature of the CMS apparatus is a superconducting solenoid of 6$\,\text {m}$ internal diameter, providing a magnetic field of 3.8$\,\text {T}$. Within the solenoid volume are a silicon pixel and strip tracker, a lead tungstate crystal electromagnetic calorimeter (ECAL), and a brass and scintillator hadron calorimeter (HCAL), each composed of a barrel and two endcap sections. Forward calorimeters extend the pseudorapidity $\eta$ coverage provided by the barrel and endcap detectors. Muons are detected in gas-ionization chambers embedded in the steel flux-return yoke outside the solenoid.

Events of interest are selected using a two-tiered trigger system. The first level, composed of custom hardware processors, uses information from the calorimeters and muon detectors to select events at a rate of around 100$\,\text {kHz}$ within a fixed latency of about $4 ~{\upmu }\hbox {s}$ [34]. The second level, known as the high-level trigger, consists of a farm of processors running a version of the full event reconstruction software optimized for fast processing, and reduces the event rate to around 1$\,\text {kHz}$ before data storage [35].

The candidate vertex with the largest value of summed physics-object squared transverse momentum $p_{{\mathrm {T}}} ^2$ is taken to be the primary ${\mathrm{p}} {\mathrm{p}}$ interaction vertex (PV). The physics objects are the jets, clustered using the jet finding algorithm [36, 37] with the tracks assigned to candidate vertices as inputs, and the associated missing transverse momentum, taken as the negative vector sum of the $p_{{\mathrm {T}}}$ of those jets.

The electron momentum is estimated by combining the energy measurement in the ECAL with the momentum measurement in the tracker. The momentum resolution for electrons with $p_{{\mathrm {T}}} \approx 45\,\text {GeV}$ from ${\mathrm{Z}} \rightarrow {\mathrm{e}} {\mathrm{e}}$ decays ranges from 1.7% to 4.5%. It is generally better in the barrel region than in the endcaps, and also depends on the bremsstrahlung energy emitted by the electron as it traverses the material in front of the ECAL [38]. The ECAL consists of 75 848 lead tungstate crystals, which provide coverage of $|\eta | < 1.48$ in the barrel region and $1.48< |\eta | < 3.00$ in the two endcap regions (EE). Preshower detectors consisting of two planes of silicon sensors interleaved with a total of $3 X_0$ of lead are located in front of each EE detector.

Muons are measured in the pseudorapidity range $|\eta | < 2.4$, with detection planes made using three technologies: drift tubes, cathode strip chambers, and resistive plate chambers. The single muon trigger efficiency exceeds 90% over the full $\eta$ range, and the efficiency to reconstruct and identify muons is greater than 96%. Matching muons to tracks measured in the silicon tracker results in a relative transverse momentum resolution, for muons with $p_{{\mathrm {T}}}$ up to 100$\,\text {GeV}$, of 1% in the barrel and 3% in the endcaps. The $p_{{\mathrm {T}}}$ resolution in the barrel is better than 7% for muons with $p_{{\mathrm {T}}}$ up to 1$\,\text {TeV}$ [39].

A more detailed description of the CMS detector, together with a definition of the coordinate system used and the relevant kinematic variables, can be found in Ref. [40].

## Data and simulated samples

This analysis is based on the ${\mathrm{p}} {\mathrm{p}}$ collision data collected by the CMS detector at the LHC in 2016, 2017, and 2018 with integrated luminosities of 35.9, 41.5, and 59.7$\,\text {fb}^{-1}$, respectively [41–43]. The collision events are selected by high-level trigger algorithms that require the presence of leptons passing loose identification and isolation requirements. The main triggers select either a pair of electrons or muons, or an electron and a muon. The minimal transverse momentum of the leading and subleading leptons changed throughout the years to account for the different data-taking conditions and is summarized in Table 1.

**Table 1:** The minimal $p_{{\mathrm {T}}}$ of the leading/subleading leptons for the main di-electron (e/e), di-muon (${{\upmu }}_{\mathrm{}}^{\mathrm{}}$/${{\upmu }}_{\mathrm{}}^{\mathrm{}}$), and electron-muon (e/${{\upmu }}_{\mathrm{}}^{\mathrm{}}$, ${{\upmu }}_{\mathrm{}}^{\mathrm{}}$/e) high-level trigger algorithms used in the ${\mathrm{H}} \rightarrow 4\ell$ analysis in 2016, 2017, and 2018

	e/e ($\text {GeV}$)	${{\upmu }}_{\mathrm{}}^{\mathrm{}}$/${{\upmu }}_{\mathrm{}}^{\mathrm{}}$ ($\text {GeV}$)	e/${{\upmu }}_{\mathrm{}}^{\mathrm{}}$, ${{\upmu }}_{\mathrm{}}^{\mathrm{}}$/e ($\text {GeV}$)
2016	17/12	17/8	17/8, 8/23
2017	23/12	17/8	23/8, 12/23
2018	23/12	17/8	23/8, 12/23

To maximize the coverage of the ${\mathrm{H}} \rightarrow 4\ell$ phase space, triggers requiring three leptons with relaxed transverse momenta thresholds and no isolation requirement are also used, as are isolated single-electron and single-muon triggers. The overall trigger efficiency for simulated signal events that pass the full event selection (described in Sect. 4) is larger than 99%. The trigger efficiency is derived from data using a sample of $4\ell$ events collected by the single-lepton triggers and a method based on the “tag and probe” technique. One of the four leptons is matched to a candidate reconstructed by the single-lepton trigger and the remaining three leptons in the event are used as probes. The probe leptons are combined in an attempt to reconstruct any of the triggers used in the analysis. The efficiency in data is found to be in agreement with the expectation from the simulation.

Monte Carlo (MC) simulation samples for the signals and the relevant background processes are used to evaluate the signal shape, estimate backgrounds, optimize the event selection, and evaluate the acceptance and systematic uncertainties. The SM H boson signals are simulated at next-to-leading order (NLO) in perturbative QCD (pQCD) with the powheg  2.0 [44–46] generator for the five main production processes: gluon fusion (${\mathrm{g}} {\mathrm{g}} {\mathrm{H}}$) [47], vector boson fusion (${\mathrm {VBF}}$) [48], associated production with a vector boson (${{\mathrm{V}}_{\mathrm{}}^{\mathrm{}}} {\mathrm{H}}$, where ${\mathrm{V}}_{\mathrm{}}^{\mathrm{}}$ is a W or a Z boson) [49], and associated production with a pair of top quarks (${{{\mathrm{t}}_{\mathrm{}}^{\mathrm{}}} {}{{{\overline{{{\mathrm{t}}_{\mathrm{}}^{\mathrm{}}}}}}_{\mathrm{}}^{\mathrm{}}}} {\mathrm{H}}$) [50]. The ${\mathrm{Z}} {\mathrm{H}}$ production occurs in two ways, ${{\mathrm{q}}_{\mathrm{}}^{\mathrm{}}} {{{\overline{{{\mathrm{q}}_{\mathrm{}}^{\mathrm{}}}}}}_{\mathrm{}}^{\mathrm{}}} \rightarrow {\mathrm{Z}} {\mathrm{H}}$ and a much smaller contribution from ${\mathrm{g}} {\mathrm{g}} \rightarrow {\mathrm{Z}} {\mathrm{H}}$, which is simulated at leading order (LO) using jhugen 7.3.0 [51–55]. In addition to the five main production processes, the contributions due to H boson production in association with a single top quark (${{\mathrm{t}}_{\mathrm{}}^{\mathrm{}}} {\mathrm{H}}$) and either a quark (${{\mathrm{t}}_{\mathrm{}}^{\mathrm{}}} {\mathrm{H}} {{\mathrm{q}}_{\mathrm{}}^{\mathrm{}}}$) or a ${\mathrm{W}}$ boson (${{\mathrm{t}}_{\mathrm{}}^{\mathrm{}}} {\mathrm{H}} {\mathrm{W}}$) are simulated at LO using jhugen 7.0.2 and MadGraph 5_amc@nlo  2.2.2 [56], respectively. The associated production with a pair of bottom quarks (${{\mathrm{b}}_{\mathrm{}}^{\mathrm{}}} {}{{{\overline{{{\mathrm{b}}_{\mathrm{}}^{\mathrm{}}}}}}_{\mathrm{}}^{\mathrm{}}} {\mathrm{H}}$) is simulated at LO with jhugen 7.0.2. In all cases, the decay of the H boson to four leptons is modeled with jhugen 7.0.2. The theoretical predictions used for the various production and decay modes can be found in Refs. [57–79] and are summarized in Ref. [32].

The ${\mathrm{Z}} {\mathrm{Z}}$ background contribution from quark-antiquark annihilation is simulated at NLO pQCD with powheg  2.0 [80], while the ${\mathrm{g}} {\mathrm{g}} \rightarrow {\mathrm{Z}} {\mathrm{Z}}$ process is generated at LO with mcfm  7.0.1 [81]. The ${\mathrm{W}} {\mathrm{Z}}$ background and the triboson backgrounds ${\mathrm{Z}} {\mathrm{Z}} {\mathrm{Z}}$, ${\mathrm{W}} {\mathrm{Z}} {\mathrm{Z}}$, and ${\mathrm{W}} {\mathrm{W}} {\mathrm{Z}}$ are modeled at NLO using MadGraph 5_amc@nlo  2.4.2. The smaller ${{{\mathrm{t}}_{\mathrm{}}^{\mathrm{}}} {}{{{\overline{{{\mathrm{t}}_{\mathrm{}}^{\mathrm{}}}}}}_{\mathrm{}}^{\mathrm{}}}} {\mathrm{Z}}$, ${{{\mathrm{t}}_{\mathrm{}}^{\mathrm{}}} {}{{{\overline{{{\mathrm{t}}_{\mathrm{}}^{\mathrm{}}}}}}_{\mathrm{}}^{\mathrm{}}}} {\mathrm{W}} {\mathrm{W}}$, and ${{{\mathrm{t}}_{\mathrm{}}^{\mathrm{}}} {}{{{\overline{{{\mathrm{t}}_{\mathrm{}}^{\mathrm{}}}}}}_{\mathrm{}}^{\mathrm{}}}} {\mathrm{Z}} {\mathrm{Z}}$ background processes are simulated at LO with MadGraph 5_amc@nlo  2.4.2. The events containing ${\mathrm{Z}}$ bosons with associated jets (${\mathrm{Z}} +$jets) are simulated at NLO with MadGraph 5_amc@nlo  2.4.2 and the ${{{\mathrm{t}}_{\mathrm{}}^{\mathrm{}}} {}{{{\overline{{{\mathrm{t}}_{\mathrm{}}^{\mathrm{}}}}}}_{\mathrm{}}^{\mathrm{}}}}$ background is simulated at NNLO with powheg  2.0. The reducible background determination does not rely on the MC but is based on data, as described in Sect. 7.2.

All signal and background event generators are interfaced with pythia 8.230 [82] using the CUETP8M1 tune [83] for the 2016 data-taking period and the CP5 tune [84] for the 2017 and 2018 data-taking periods, to simulate the multi-parton interaction and hadronization effects. The NNPDF3.0 set of parton distribution functions (PDFs) is used [85]. The generated events are processed through a detailed simulation of the CMS detector based on Geant4 [86, 87] and are reconstructed with the same algorithms that are used for data. The simulated events include overlapping ${\mathrm{p}} {\mathrm{p}}$ interactions (pileup) and have been reweighted so that the distribution of the number of interactions per LHC bunch crossing in simulation matches that observed in data.

## Event reconstruction and selection

The particle-flow (PF) algorithm [88] aims to reconstruct and identify each individual particle (PF candidate) in an event, with an optimized combination of information from the various elements of the CMS detector. The energy of photons is obtained from the ECAL measurement. The energy of electrons is determined from a combination of the electron momentum at the PV as determined by the tracker, the energy of the corresponding ECAL cluster, and the energy sum of all bremsstrahlung photons spatially compatible with originating from the electron track. The energy of muons is obtained from the curvature of the corresponding track. The energy of charged hadrons is determined from a combination of their momentum measured in the tracker and the matching ECAL and HCAL energy deposits, corrected for the response function of the calorimeters to hadronic showers. Finally, the energy of neutral hadrons is obtained from the corresponding ECAL and HCAL energies.

The missing transverse momentum vector ${\vec {p}}_{{\mathrm {T}}}^{\text {miss}}$ is computed as the negative vector sum of the transverse momenta of all the PF candidates in an event, and its magnitude is denoted as $p_{{\mathrm {T}}} ^\text {miss}$ [89]. The ${\vec {p}}_{{\mathrm {T}}}^{\text {miss}}$ is modified to account for corrections to the energy scale of the reconstructed jets in the event.

Muons with $p_{{\mathrm {T}}} ^{{{{\upmu }}_{\mathrm{}}^{\mathrm{}}}} > 5\,\text {GeV}$ are reconstructed within the geometrical acceptance, corresponding to the region $|\eta ^{{{{\upmu }}_{\mathrm{}}^{\mathrm{}}}} | < 2.4$, by combining information from the silicon tracker and the muon system [39]. The matching between the inner and outer tracks proceeds either outside-in, starting from a track in the muon system, or inside-out, starting from a track in the silicon tracker. Inner tracks that match segments in only one or two stations of the muon system are also considered because they may belong to very low-$p_{{\mathrm {T}}}$ muons that do not have sufficient energy to penetrate the entire muon system. The muons are selected among the reconstructed muon track candidates by applying minimal requirements on the track in both the muon system and the inner tracker system, and taking into account the compatibility with small energy deposits in the calorimeters.

To discriminate between prompt muons from Z boson decay and those arising from electroweak (EW) decays of hadrons within jets, an isolation requirement of ${{\mathcal {I}}}^{{{{\upmu }}_{\mathrm{}}^{\mathrm{}}}}<0.35$ is imposed, where the relative isolation is defined as

1 $$\begin{aligned} {{\mathcal {I}}}^{{{{\upmu }}_{\mathrm{}}^{\mathrm{}}}}\equiv & {} \Big ( \sum p_{{\mathrm {T}}} ^\text {charged} + \max \big [ 0, \sum p_{{\mathrm {T}}} ^\text {neutral} \nonumber \\&+\sum p_{{\mathrm {T}}} ^{{{\upgamma }}} - p_{{\mathrm {T}}} ^{{{{\upmu }}_{\mathrm{}}^{\mathrm{}}},{\mathrm {PU}}} \big ] \Big ) / p_{{\mathrm {T}}} ^{{{{\upmu }}_{\mathrm{}}^{\mathrm{}}}} . \end{aligned}$$

In Eq. (1), $\sum p_{{\mathrm {T}}} ^\text {charged}$ is the scalar sum of the transverse momenta of charged hadrons originating from the chosen PV of the event. The quantities $\sum p_{{\mathrm {T}}} ^\text {neutral}$ and $\sum p_{{\mathrm {T}}} ^{{{\upgamma }}}$ are the scalar sums of the transverse momenta for neutral hadrons and photons, respectively. The isolation sums involved are all restricted to a volume bound by a cone of angular radius $\varDelta R=0.3$ around the muon direction at the PV, where the angular distance between two particles *i* and *j* is $\varDelta R(i,j) = \sqrt{\smash [b]{(\eta ^i-\eta ^j)^{2} + (\phi ^i-\phi ^j)^{2}}}$. Since the isolation variable is particularly sensitive to energy deposits from pileup interactions, a $p_{{\mathrm {T}}} ^{{{{\upmu }}_{\mathrm{}}^{\mathrm{}}},{\mathrm {PU}}}$ contribution is subtracted, defined as $p_{{\mathrm {T}}} ^{{{{\upmu }}_{\mathrm{}}^{\mathrm{}}},{\mathrm {PU}}}\equiv 0.5\sum _{i}p_{{\mathrm {T}}} ^{\textit{i},\text {PU}}$, where *i* runs over the charged hadron PF candidates not originating from the PV, and the factor of 0.5 corrects for the different fraction of charged and neutral particles in the cone [90].

Electrons with $p_{{\mathrm {T}}} ^{{\mathrm{e}}} > 7\,\text {GeV}$ are reconstructed within the geometrical acceptance, corresponding to the pseudorapidity region $|\eta ^{{\mathrm{e}}} | < 2.5$ [38]. Electrons are identified using a multivariate discriminant which includes observables sensitive to the presence of bremsstrahlung along the electron trajectory, the geometrical and momentum–energy matching between the electron trajectory and the associated cluster in the ECAL, the shape of the electromagnetic shower in the ECAL, and variables that discriminate against electrons originating from photon conversions. Instead of an additional isolation requirement, similar to the one for muons, the electron multivariate discriminant also includes the isolation sums described above ($\sum p_{{\mathrm {T}}} ^{\text {charged}}$, $\sum p_{{\mathrm {T}}} ^{\text {neutral}}$, and $\sum p_{{\mathrm {T}}} ^{{{\upgamma }}}$) but computed around the electron direction. The inclusion of isolation sums helps suppressing electrons originating from electroweak decays of hadrons within jets [91] and has a better performance than a simple requirement on the relative isolation observable. The package xgboost [92] is used for the training and optimization of the multivariate discriminant employed for electron identification and isolation. The training is performed with simulated events that are not used at any other stage of the analysis. Events are divided into six regions defined by two transverse momentum ranges (7–10 $\,\text {GeV}$ and $>10\,\text {GeV}$) and three pseudorapidity regions: central barrel ($|\eta ^{{\mathrm{e}}} |<0.8$), outer barrel ($0.8<|\eta ^{{\mathrm{e}}} |<1.479$), and endcaps ($1.479<|\eta ^{{\mathrm{e}}} |<2.5$). Separate training is performed for the three different data-taking periods and selection requirements are determined such that the signal efficiency remains the same for all three periods.

The effect of the final-state radiation (FSR) from leptons is recovered as follows. Bremsstrahlung photons already associated to electrons in the reconstruction step are not considered in this procedure. Photons reconstructed by the PF algorithm within $|\eta ^{{\upgamma }} |<2.4$ are considered as FSR candidates if they satisfy the conditions $p_{{\mathrm {T}}} ^{{{\upgamma }}} > 2 \,\text {GeV}$ and ${{\mathcal {I}}}^{{{\upgamma }}} < 1.8$, where the photon relative isolation ${{\mathcal {I}}}^{{{\upgamma }}}$ is defined as for the muon in Eq. (1). Every such photon is associated to the closest selected lepton in the event. Photons that do not satisfy the requirements $\varDelta R({{\upgamma }},\ell )/(p_{{\mathrm {T}}} ^{{{\upgamma }}})^2<0.012 \,\text {GeV} ^{-2}$ and $\varDelta R({{\upgamma }},\ell ) < 0.5$ are discarded. The lowest-$\varDelta R({{\upgamma }},\ell )/(p_{{\mathrm {T}}} ^{{{\upgamma }}})^2$ photon candidate of every lepton, if any, is retained. The photons thus identified are excluded from the isolation computation of the muons selected in the event.

In order to suppress muons from in-flight decays of hadrons and electrons from photon conversions, leptons are rejected if the ratio of their impact parameter in three dimensions, computed with respect to the PV position, to their uncertainty is greater or equal to four.

The momentum scale and resolution of electrons and muons are calibrated in bins of $p_{{\mathrm {T}}} ^{\ell }$ and $\eta ^{\ell }$ using the decay products of known dilepton resonances as described in Refs. [38, 39].

A “tag and probe” technique [93] based on samples of ${\mathrm{Z}}$ boson events in data and simulation is used to measure the efficiency of the reconstruction and selection for prompt electrons and muons in several bins of $p_{{\mathrm {T}}} ^{\ell }$ and $\eta ^{\ell }$. The difference in the efficiencies measured in simulation and data is used to rescale the yields of selected events in the simulated samples.

For each event, hadronic jets are clustered from the reconstructed particles using the infrared- and collinear-safe anti-$k_{{\mathrm {T}}}$ algorithm [36, 37] with a distance parameter of 0.4. The jet momentum is determined as the vectorial sum of all particle momenta in the jet, and is found from simulation to be within 5–10% of the true momentum over the whole $p_{{\mathrm {T}}}$ spectrum and detector acceptance. Additional ${\mathrm{p}} {\mathrm{p}}$ interactions within the same or nearby bunch crossings can contribute extra tracks and calorimetric energy depositions to the jet. To mitigate this effect, tracks identified as originating from pileup vertices are discarded and an offset correction is applied to correct for the remaining contributions. Jet energy corrections are derived from simulation to match that of particle level jets on average. In situ measurements of the momentum balance in dijet, photon + jet, ${\mathrm{Z}} +$ jet, and multijet events are used to account for any residual differences in jet energy scale in data and simulation [94]. Jet energies in simulation are smeared to match the resolution in data. The jet energy resolution amounts typically to 16% at 30$\,\text {GeV}$, 8% at 100$\,\text {GeV}$, and 4% at 1$\,\text {TeV}$. Additional selection criteria are applied to remove jets potentially dominated by anomalous contributions from various subdetector components or reconstruction failures. To be considered in the analysis, jets must satisfy the conditions $p_{{\mathrm {T}}} ^{\text {jet}}>30\,\text {GeV}$ and $|\eta ^{\text {jet}} | < 4.7$, and be separated from all selected lepton candidates and any selected FSR photons by $\varDelta R(\ell /{{\upgamma }},\text {jet})>0.4$. Jets are also required to pass the tight identification criteria and the tight working point of pileup jet identification described in Ref. [90].

For event categorization, jets are tagged as ${\mathrm{b}}_{\mathrm{}}^{\mathrm{}}$ jets using the DeepCSV algorithm [95], which combines information about impact parameter significance, secondary vertex, and jet kinematics. Data to simulation scale factors for the ${\mathrm{b}}_{\mathrm{}}^{\mathrm{}}$ tagging efficiency are applied as a function of jet $p_{{\mathrm {T}}}$, $\eta$, and flavor.

The event selection is designed to extract signal candidates from events containing at least four well-identified and isolated leptons, each originating from the PV and possibly accompanied by an FSR photon candidate. In what follows, unless otherwise stated, FSR photons are included in invariant mass computations.

First, ${\mathrm{Z}}$ candidates are formed with pairs of leptons of the same flavor and opposite-charge (${{\mathrm{{\mathrm{e}}}}_{\mathrm{}}^{\mathrm{+}}} {{\mathrm{{\mathrm{e}}}}_{\mathrm{}}^{\mathrm{-}}}$, ${{{{{{\upmu }}_{\mathrm{}}^{\mathrm{}}}}}_{\mathrm{}}^{\mathrm{+}}} {{{{{{\upmu }}_{\mathrm{}}^{\mathrm{}}}}}_{\mathrm{}}^{\mathrm{-}}}$) that pass the requirement $12< m_{\ell ^{+}\ell ^{-}}< 120\,\text {GeV}$. They are then combined into ${\mathrm{Z}} {\mathrm{Z}}$ candidates, wherein we denote as ${\mathrm{Z}} _1$ the ${\mathrm{Z}}$ candidate with an invariant mass closest to the nominal ${\mathrm{Z}}$ boson mass [96], and as ${\mathrm{Z}} _2$ the other one. The flavors of the involved leptons define three mutually exclusive subchannels: $4{\mathrm{e}}$, $4{{{\upmu }}_{\mathrm{}}^{\mathrm{}}}$, and $2{\mathrm{e}} 2{{{\upmu }}_{\mathrm{}}^{\mathrm{}}}$.

To be considered for the analysis, ${\mathrm{Z}} {\mathrm{Z}}$ candidates have to pass a set of kinematic requirements that improve the sensitivity to H boson decays. The ${\mathrm{Z}} _1$ invariant mass must be larger than 40$\,\text {GeV}$. All leptons must be separated in angular space by at least $\varDelta R(\ell _i, \ell _j) > 0.02$. At least two leptons are required to have $p_{{\mathrm {T}}} > 10\,\text {GeV}$ and at least one is required to have $p_{{\mathrm {T}}} > 20\,\text {GeV}$. In the $4{{{\upmu }}_{\mathrm{}}^{\mathrm{}}}$ and $4{\mathrm{e}}$ subchannels, where an alternative ${\mathrm{Z}} _a {\mathrm{Z}} _b$ candidate can be built out of the same four leptons, we discard candidates with $m_{{\mathrm{Z}} _b}<12\,\text {GeV}$ if ${\mathrm{Z}} _a$ is closer to the nominal ${\mathrm{Z}}$ boson mass than ${\mathrm{Z}} _1$ is. This rejects events that contain an on-shell ${\mathrm{Z}}$ and a low-mass dilepton resonance. To further suppress events with leptons originating from hadron decays in jet fragmentation or from the decay of low-mass resonances, all four opposite-charge lepton pairs that can be built with the four leptons (irrespective of flavor) are required to satisfy the condition $m_{\ell ^{+}\ell '^{-}} > 4\,\text {GeV}$, where selected FSR photons are disregarded in the invariant mass computation. Finally, the four-lepton invariant mass $m_{4\ell }$ must be larger than 70$\,\text {GeV}$, which defines the mass range of interest for the subsequent steps of the analysis.

In events where more than one ${\mathrm{Z}} {\mathrm{Z}}$ candidate passes the above selection, the candidate with the highest value of ${{\mathcal {D}}}^{\text {kin}}_{\text {bkg}}$ (defined in Sect. 5) is retained, except if two candidates consist of the same four leptons, in which case the candidate with the ${\mathrm{Z}} _1$ mass closest to the nominal ${\mathrm{Z}}$ boson mass is retained.

## Kinematic discriminants

The full kinematic information from each event using either the H boson decay products and/or the associated particles in the H boson production is extracted by means of matrix element calculations and is used to form several kinematic discriminants. These computations rely on the MELA package [51–53, 55] and exploit the jhugen matrix elements for the signal and the mcfm matrix elements for the background. Both the H boson decay kinematics and the kinematics of the associated production of ${\mathrm{H}} +1~$jet, ${\mathrm{H}} +2$ jets, ${\mathrm {VBF}}$, ${\mathrm{Z}} {\mathrm{H}}$, and ${\mathrm{W}} {\mathrm{H}}$ are explored. The full event kinematics is described by decay observables $\vec \varOmega ^{{\mathrm{H}} \rightarrow 4\ell }$ or observables describing the associated production $\vec \varOmega ^{{\mathrm{H}} +\text {jj}}$, which may or may not include the ${\mathrm{H}} \rightarrow 4\ell$ decay kinematic information depending on the use case. The definition of these observables can be found in Refs. [51–53].

Two types of kinematic discriminants are exploited in the ${\mathrm{H}} \rightarrow 4\ell$ analysis. First we construct the three categorization discriminants in order to classify signal events into exclusive categories as defined in Sect. 6.2. Categorization discriminants are designed to increase the purity of the targeted production mechanism in a dedicated event category. In addition, we define another set of three kinematic discriminants that are taken as an observable in the two-dimensional likelihood fits carried out to extract the results, as described in Sect. 10. These kinematic discriminants are designed to separate the targeted H boson production mechanism from its dominant background.

Categorization discriminants are calculated following the prescription in Refs. [18, 21, 97]. The discriminants sensitive to the ${\mathrm {VBF}}$ signal topology with two associated jets, the ${\mathrm {VBF}}$ signal topology with one associated jet, and the ${{\mathrm{V}}_{\mathrm{}}^{\mathrm{}}} {\mathrm{H}}$ (either ${\mathrm{Z}} {\mathrm{H}}$ or ${\mathrm{W}} {\mathrm{H}}$) signal topology with two associated jets are:

2 $$\begin{aligned} \begin{aligned} {{\mathcal {D}}}_{\text {2jet}}^{{\mathrm {VBF}}}&= \left[ 1+ \frac{ {\mathcal {P}}_{{\mathrm{H}} \text {jj}} (\vec \varOmega ^{{\mathrm{H}} +\text {jj}} | m_{4\ell }) }{{\mathcal {P}}_{{\mathrm {VBF}}} (\vec \varOmega ^{{\mathrm{H}} +\text {jj}} | m_{4\ell }) } \right] ^{-1}\\ {{\mathcal {D}}}_{\text {1jet}}^{{\mathrm {VBF}}}&= \left[ 1+ \frac{ {\mathcal {P}}_{{\mathrm{H}} \text {j}} (\vec \varOmega ^{{\mathrm{H}} +\text {j}} | m_{4\ell }) }{\int d\eta ^{\text {j}}{\mathcal {P}}_{{\mathrm {VBF}}} (\vec \varOmega ^{{\mathrm{H}} +\text {jj}} | m_{4\ell }) } \right] ^{-1} \\ {{\mathcal {D}}}_{\text {2jet}}^{{\mathrm{W}} {\mathrm{H}}}&= \left[ 1+ \frac{ {\mathcal {P}}_{{\mathrm{H}} \text {jj}} (\vec \varOmega ^{{\mathrm{H}} +\text {jj}} | m_{4\ell }) }{{\mathcal {P}}_{{\mathrm{W}} {\mathrm{H}}} (\vec \varOmega ^{{\mathrm{H}} +\text {jj}} | m_{4\ell }) } \right] ^{-1} \\ {{\mathcal {D}}}_{\text {2jet}}^{{\mathrm{Z}} {\mathrm{H}}}&= \left[ 1+ \frac{ {\mathcal {P}}_{{\mathrm{H}} \text {jj}} (\vec \varOmega ^{{\mathrm{H}} +\text {jj}} | m_{4\ell }) }{{\mathcal {P}}_{{\mathrm{Z}} {\mathrm{H}}} (\vec \varOmega ^{{\mathrm{H}} +\text {jj}} | m_{4\ell }) } \right] ^{-1}, \end{aligned} \end{aligned}$$

where ${\mathcal {P}}_{{\mathrm {VBF}}}$, ${\mathcal {P}}_{{\mathrm{H}} \text {jj}}$, ${\mathcal {P}}_{{\mathrm{H}} \text {j}}$, and ${\mathcal {P}}_{{{\mathrm{V}}_{\mathrm{}}^{\mathrm{}}} {\mathrm{H}}}$ are the probabilities for the ${\mathrm {VBF}}$ process, the ${\mathrm{g}} {\mathrm{g}} {\mathrm{H}}$ process in association with two jets (combination of ${\mathrm{g}} {\mathrm{g}}/{{\mathrm{q}}_{\mathrm{}}^{\mathrm{}}} {\mathrm{g}}/{{\mathrm{q}}_{\mathrm{}}^{\mathrm{}}} {{\mathrm{q}}_{\mathrm{}}^{\mathrm{}}} ^\prime$ parton collisions producing ${\mathrm{H}} +2$ jets), the ${\mathrm{g}} {\mathrm{g}} {\mathrm{H}}$ process in association with one jet (${\mathrm{H}} +1$ jet), and the ${{\mathrm{V}}_{\mathrm{}}^{\mathrm{}}} {\mathrm{H}}$ process, respectively. The quantity $\int d\eta ^{\text {j}}{\mathcal {P}}_{{\mathrm {VBF}}}$ is the integral of the two-jet ${\mathrm {VBF}}$ matrix element probability over the $\eta ^j$ values of the unobserved jet, with the constraint that the total transverse momentum of the ${\mathrm{H}} +2$ jets system is zero. The discriminant ${{\mathcal {D}}}_{\text {2jet}}^{{{\mathrm{V}}_{\mathrm{}}^{\mathrm{}}} {\mathrm{H}}}$, used for event categorization, is defined as the maximum value of the two discriminants, ${{\mathcal {D}}}_{\text {2jet}}^{{{\mathrm{V}}_{\mathrm{}}^{\mathrm{}}} {\mathrm{H}}} = \max ({{\mathcal {D}}}_{\text {2jet}}^{{\mathrm{Z}} {\mathrm{H}}},{{\mathcal {D}}}_{\text {2jet}}^{{\mathrm{W}} {\mathrm{H}}})$.

A set of three discriminants used in the likelihood fits is calculated as in Refs. [17, 18]. The discriminant sensitive to the ${\mathrm{g}} {\mathrm{g}}/{{\mathrm{q}}_{\mathrm{}}^{\mathrm{}}} {{{\overline{{{\mathrm{q}}_{\mathrm{}}^{\mathrm{}}}}}}_{\mathrm{}}^{\mathrm{}}} \rightarrow 4\ell$ process exploits the kinematics of the four-lepton decay system. It is used in most of the event categories described in Sect. 6 to separate signal from background and is defined as:

3 $$\begin{aligned} {\mathcal {D}}^{\text {kin}}_{\text {bkg}} = \left[ 1+ \frac{ {\mathcal {P}}^{{{\mathrm{q}}_{\mathrm{}}^{\mathrm{}}} {{{\overline{{{\mathrm{q}}_{\mathrm{}}^{\mathrm{}}}}}}_{\mathrm{}}^{\mathrm{}}}}_\text {bkg} (\vec \varOmega ^{{\mathrm{H}} \rightarrow 4\ell } | m_{4\ell }) }{ {\mathcal {P}}^{{\mathrm{g}} {\mathrm{g}}}_\text {sig}(\vec \varOmega ^{{\mathrm{H}} \rightarrow 4\ell } | m_{4\ell }) } \right] ^{-1}, \end{aligned}$$

where ${\mathcal {P}}^{{\mathrm{g}} {\mathrm{g}}}_\text {sig}$ is the probability for the signal and ${\mathcal {P}}^{{{\mathrm{q}}_{\mathrm{}}^{\mathrm{}}} {{{\overline{{{\mathrm{q}}_{\mathrm{}}^{\mathrm{}}}}}}_{\mathrm{}}^{\mathrm{}}}}_\text {bkg}$ is the probability for the dominant ${{\mathrm{q}}_{\mathrm{}}^{\mathrm{}}} {{{\overline{{{\mathrm{q}}_{\mathrm{}}^{\mathrm{}}}}}}_{\mathrm{}}^{\mathrm{}}} \rightarrow 4\ell$ background process, calculated using the LO matrix elements. In the ${\mathrm {VBF}}$-2jet-tagged and ${{\mathrm{V}}_{\mathrm{}}^{\mathrm{}}} {\mathrm{H}}$-hadronic-tagged event categories (defined in Sect. 6.2), the background includes the QCD production of ${\mathrm{Z}} {\mathrm{Z}}/{\mathrm{Z}} {{\upgamma }} ^{*}/{{\upgamma }} ^{*}{{\upgamma }} ^{*}\rightarrow 4\ell$ in association with two jets, the EW background from the vector boson scattering (VBS), as well as the triboson (${{\mathrm{V}}_{\mathrm{}}^{\mathrm{}}} {{\mathrm{V}}_{\mathrm{}}^{\mathrm{}}} {{\mathrm{V}}_{\mathrm{}}^{\mathrm{}}}$) production process. We therefore use dedicated production-dependent discriminants based on the kinematics of the four-lepton decay and information from the associated jets (noted with ${\mathrm {VBF}}+$ dec or ${{\mathrm{V}}_{\mathrm{}}^{\mathrm{}}} {\mathrm{H}} +$ dec), defined as:

4 $$\begin{aligned}&{{\mathcal {D}}}^{{\mathrm {VBF}}+\text {dec}}_{\text {bkg}} \nonumber \\&= \Bigg [1+\frac{c^{{\mathrm {VBF}}}(m_{4\ell }) [{\mathcal {P}}^{\text {EW}}_\text {bkg}(\vec \varOmega ^{{\mathrm{H}} +\text {jj}}| m_{4\ell })+{\mathcal {P}}^{{\mathrm {QCD}}}_\text {bkg}(\vec \varOmega ^{{\mathrm{H}} +\text {jj}}| m_{4\ell })]}{{\mathcal {P}}^{\text {EW}}_{{\mathrm {sig}}}(\vec \varOmega ^{{\mathrm{H}} +\text {jj}}| m_{4\ell })} \Bigg ]^{-1} \end{aligned}$$

5 $$\begin{aligned}&{{\mathcal {D}}}^{{{\mathrm{V}}_{\mathrm{}}^{\mathrm{}}} {\mathrm{H}} +\text {dec}}_{\text {bkg}} \nonumber \\&\quad = \Bigg [1+\frac{c^{{{\mathrm{V}}_{\mathrm{}}^{\mathrm{}}} {\mathrm{H}}}(m_{4\ell }) [{\mathcal {P}}^{\text {EW}}_\text {bkg}(\vec \varOmega ^{{\mathrm{H}} +\text {jj}}| m_{4\ell })+{\mathcal {P}}^{{\mathrm {QCD}}}_\text {bkg}(\vec \varOmega ^{{\mathrm{H}} +\text {jj}}| m_{4\ell })]}{{\mathcal {P}}^{\text {EW}}_{{\mathrm {sig}}}(\vec \varOmega ^{{\mathrm{H}} +\text {jj}}| m_{4\ell })} \Bigg ]^{-1},\nonumber \\ \end{aligned}$$

where ${\mathcal {P}}^{\text {EW}}_{{\mathrm {sig}}}$ is the probability for the ${\mathrm {VBF}}$ and ${{\mathrm{V}}_{\mathrm{}}^{\mathrm{}}} {\mathrm{H}}$ signal, ${\mathcal {P}}^{\text {EW}}_\text {bkg}$ is the probability for the VBS and VVV background processes, and ${\mathcal {P}}^{{\mathrm {QCD}}}_\text {bkg}$ is the probability for ${\mathrm{Z}} {\mathrm{Z}}/{\mathrm{Z}} {{\upgamma }} ^{*}/{{\upgamma }} ^{*}{{\upgamma }} ^{*}\rightarrow 4\ell$ QCD production in association with two jets. The quantity $c^{p}(m_{4\ell })$ for category *p* is the $m_{4\ell }$-dependent parameter that allows to change the relative normalization of the EW probabilities, separately for the ${\mathrm {VBF}}$ and ${{\mathrm{V}}_{\mathrm{}}^{\mathrm{}}} {\mathrm{H}}$ topologies. For each slice of $m_{4\ell }$, the distributions of the signal and background discriminants are plotted, and the $c^{p}(m_{4\ell })$ value is determined in such a way that the two distributions cross at 0.5. This procedure allows rescaling of the distributions for the linear-scale binning of the templates used in the likelihood fits described in Sect. 10.

## Event categorization

In order to improve the sensitivity to the H boson production mechanisms, the selected events are classified into mutually exclusive categories based on the features of the reconstructed objects associated with the ${\mathrm{H}} \rightarrow 4\ell$ candidates. Event categorization is organized in two steps with increasing granularity of the categories. First step is primarily designed to separate the ${\mathrm{g}} {\mathrm{g}} {\mathrm{H}}$, ${\mathrm {VBF}}$, ${{\mathrm{V}}_{\mathrm{}}^{\mathrm{}}} {\mathrm{H}}$, and ${{{\mathrm{t}}_{\mathrm{}}^{\mathrm{}}} {}{{{\overline{{{\mathrm{t}}_{\mathrm{}}^{\mathrm{}}}}}}_{\mathrm{}}^{\mathrm{}}}} {\mathrm{H}}$ processes. There is little sensitivity to ${{\mathrm{b}}_{\mathrm{}}^{\mathrm{}}} {}{{{\overline{{{\mathrm{b}}_{\mathrm{}}^{\mathrm{}}}}}}_{\mathrm{}}^{\mathrm{}}} {\mathrm{H}}$ or ${{\mathrm{t}}_{\mathrm{}}^{\mathrm{}}} {\mathrm{H}}$, even though these production modes are considered explicitly in the analysis. The reconstructed event categories from the first step are further subdivided (as discussed in Sect. 6.2) in order to study each production mechanism in more detail. This subdivision is carried out by matching the recommended binning of the framework of STXS described in the following section.

### STXS production bins

The STXS framework has been adopted by the LHC experiments as a common framework for studies of the H boson. It has been developed to define fine-grained measurements of the H boson production modes in various kinematic regions, and to reduce the theoretical uncertainties that are folded into the measurements. It also allows for the use of advanced categorization techniques and provides a common scheme for combining measurements in different decay channels or by different experiments. The regions of phase space defined by this framework are referred to as production bins and are determined by using generator-level information for H bosons with rapidity $|y_{\mathrm{H}} |<2.5$. Generator-level jets are defined as anti-$k_{{\mathrm {T}}}$ jets with a distance parameter of 0.4 and a $p_{{\mathrm {T}}}$ threshold of 30$\,\text {GeV}$; no requirement is placed on the generator-level leptons.

The STXS framework has been designed to complement the Run I measurements of the production signal strength modifiers and fiducial differential cross sections of the H boson by combining their advantages. The sensitivity to theoretical uncertainties in the signal strength modifier results is suppressed by excluding dominant theoretical uncertainties causing production bin migration effects from the STXS measurements. They are included only when comparing the results with the theoretical predictions. In contrast to the fiducial differential cross section measurements, in the STXS framework measurements are optimized for sensitivity by means of event categories and matrix element discriminants. To account for the evolving experimental sensitivity, different stages of production bins with increasing granularity are developed.

The stage 0 production bins are called ${\mathtt {ggH}}$, ${\mathtt {qqH}}$, ${\mathtt {VH}}\text {-}{\mathtt {lep}}$, and ${\mathtt {ttH}}$ and are designed to closely match the main H boson production mechanisms. The ${\mathtt {qqH}}$ bin includes the EW production of the H boson in association with two quarks from either ${\mathrm {VBF}}$ or ${{\mathrm{V}}_{\mathrm{}}^{\mathrm{}}} {\mathrm{H}}$ events with hadronic decays of the vector boson ${\mathrm{V}}_{\mathrm{}}^{\mathrm{}}$. The ${\mathtt {VH}}\text {-}{\mathtt {lep}}$ production bin includes ${{\mathrm{V}}_{\mathrm{}}^{\mathrm{}}} {\mathrm{H}}$ events with leptonic decays of the vector boson ${\mathrm{V}}_{\mathrm{}}^{\mathrm{}}$. The low rate ${{\mathrm{b}}_{\mathrm{}}^{\mathrm{}}} {}{{{\overline{{{\mathrm{b}}_{\mathrm{}}^{\mathrm{}}}}}}_{\mathrm{}}^{\mathrm{}}} {\mathrm{H}}$ and ${{\mathrm{t}}_{\mathrm{}}^{\mathrm{}}} {\mathrm{H}}$ production processes are considered together with the ${\mathtt {ggH}}$ and ${\mathtt {ttH}}$ production bins, respectively. In this analysis, a modified version of the stage 0 production bins is also studied, where instead of ${\mathtt {VH}}\text {-}{\mathtt {lep}}$ and ${\mathtt {qqH}}$ bins we define the ${\mathtt {WH}}$, ${\mathtt {ZH}}$, and ${\mathtt {VBF}}$ bins that map the H boson production mechanisms without the splitting of the ${{\mathrm{V}}_{\mathrm{}}^{\mathrm{}}} {\mathrm{H}}$ events in leptonic and hadronic decays.

**Fig. 1:**
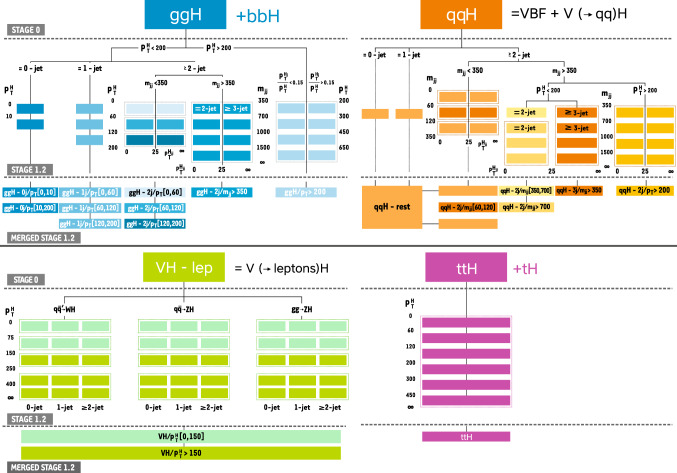
Binning of the gluon fusion production process, the electroweak production process (combines ${\mathrm {VBF}}$ and ${{\mathrm{V}}_{\mathrm{}}^{\mathrm{}}} {\mathrm{H}}$ with hadronic ${\mathrm{V}}_{\mathrm{}}^{\mathrm{}}$ decay), the ${{\mathrm{V}}_{\mathrm{}}^{\mathrm{}}} {\mathrm{H}}$ production process with leptonic ${\mathrm{V}}_{\mathrm{}}^{\mathrm{}}$ decay (combining ${\mathrm{W}} {\mathrm{H}}$, ${\mathrm{Z}} {\mathrm{H}}$, and gluon fusion ${\mathrm{Z}} {\mathrm{H}}$ production), and the ${{{\mathrm{t}}_{\mathrm{}}^{\mathrm{}}} {}{{{\overline{{{\mathrm{t}}_{\mathrm{}}^{\mathrm{}}}}}}_{\mathrm{}}^{\mathrm{}}}} {\mathrm{H}}$ production process in the merged stage 1.2 of the STXS framework used in the ${\mathrm{H}} \rightarrow {\mathrm{Z}} {\mathrm{Z}} \rightarrow 4\ell$ analysis

Stage 1 of the STXS framework was designed by further splitting the bins from the stage 0, one of the main motives being the enhanced sensitivity to possible signatures of BSM physics. This is achieved by dividing stage 0 bins with additional requirements on generator-level quantities that include the transverse momentum of the H boson ($p_{{\mathrm {T}}} ^{{\mathrm{H}}}$), the number of associated jets ($N^{\text {j}}$), the dijet invariant mass ($m_{\text {jj}}$), the transverse momentum of the H boson and the leading jet ($p_{{\mathrm {T}}} ^{{\mathrm{H}} \text {j}}$), and the transverse momentum of the H boson and the two leading jets ($p_{{\mathrm {T}}} ^{{\mathrm{H}} \text {jj}}$). These bins were designed in order to maximize sensitivity to new physics while also taking into account the current experimental sensitivity limited mostly by the amount of collected data. The most recent set of bins defined in the STXS framework is referred to as stage 1.2. This paper presents a first set of cross section measurements in the ${\mathrm{H}} \rightarrow 4\ell$ channel for the stage 1.2 of the STXS framework. However, several stage 1.2 production bins are merged as the full set of bins cannot be measured with the current data sample. The merging scheme results in 19 production bins; it is illustrated in Fig. 1 and discussed in more detail below.

The ${\mathrm{g}} {\mathrm{g}} {\mathrm{H}}$ production process is split into events with $p_{{\mathrm {T}}} ^{{\mathrm{H}}}\!<\!200\,\text {GeV}$ and $p_{{\mathrm {T}}} ^{{\mathrm{H}}}\!>\!200\,\text {GeV}$. The events with $p_{{\mathrm {T}}} ^{{\mathrm{H}}}\!>\!200\,\text {GeV}$ are placed into one single production bin called ${\mathtt {ggH/p}}_{\mathtt {T}}{\mathtt{>200}}$. The events with $p_{{\mathrm {T}}} ^{{\mathrm{H}}}<200\,\text {GeV}$ are split in events with zero, one, and two or more jets. The events with zero or one jets are split into the following production bins according to the H boson $p_{{\mathrm {T}}}$: ${\mathtt {ggH}}\text {-}{\mathtt {0j/p}}_{\mathtt {T}}{\mathtt{[0,10]}}$, ${\mathtt {ggH}}\text {-}{\mathtt {0j/p}}_{\mathtt {T}}{\mathtt{[10,200]}}$, ${\mathtt {ggH}}\text {-}{\mathtt {1j/p}}_{\mathtt {T}}{\mathtt{[0,60]}}$, ${\mathtt {ggH}}\text {-}\mathtt {1j/}\mathtt {p}_{\mathtt {T}}{\mathtt{[60,120]}}$, and ${\mathtt {ggH}}\text {-}{\mathtt {1j/p}}_{\mathtt {T}}{\mathtt{[120,200]}}$. The events with two or more jets are split according to the dijet invariant mass as follows. The events with $m_{\text {jj}}<350\,\text {GeV}$ are split into three production bins according to the H boson $p_{{\mathrm {T}}}$: ${\mathtt {ggH}}\text {-}{\mathtt {2j/p}}_{\mathtt {T}}{\mathtt{[0,60]}}$, ${\mathtt {ggH}}\text {-}{\mathtt {2j/p}}_{\mathtt {T}}{\mathtt{[60,120]}}$, and ${\mathtt {ggH}}\text {-}{\mathtt {2j/p}}_{\mathtt {T}}{\mathtt{[120,200]}}$. The events with $m_{\text {jj}}>350\,\text {GeV}$ are all placed into one production bin ${\mathtt {ggH}}\text {-}{\mathtt {2j/m}}_{\mathtt {{jj}}}{\mathtt{>350}}$, which merges four bins originally suggested in stage 1.2 of the STXS framework.

The merging scheme of the electroweak ${\mathtt {qqH}}$ production bins is as follows. The events with zero jets, one jet, or with two or more jets with $m_{jj}<60\,\text {GeV}$ or $120<m_{\text {jj}}<350\,\text {GeV}$ correspond to production bins with insufficient statistics; they are all merged into one bin called ${\mathtt {qqH}}\text {-}{\mathtt {rest}}$. The events with two or more jets and $60<m_{\text {jj}}<120\,\text {GeV}$ are placed in the ${\mathtt {qqH}}\text {-}{\mathtt {2j/m}}_{\mathtt {{jj}}}{\mathtt{[60,120]}}$ bin. The events with two or more jets and $m_{\text {jj}}>350\,\text {GeV}$ are split into events with $p_{{\mathrm {T}}} ^{{\mathrm{H}}}<200\,\text {GeV}$ and $p_{{\mathrm {T}}} ^{{\mathrm{H}}}>200\,\text {GeV}$. The events with $p_{{\mathrm {T}}} ^{{\mathrm{H}}}>200\,\text {GeV}$ are placed into one single production bin called ${\mathtt {qqH}}\text {-}{\mathtt {2j/p}}_{\mathtt {T}}{\mathtt{>200}}$. The events with $p_{{\mathrm {T}}} ^{{\mathrm{H}}}<200\,\text {GeV}$ and $p_{{\mathrm {T}}} ^{{\mathrm{H}} \text {jj}}<25\,\text {GeV}$ are split into two production bins, ${\mathtt {qqH}}\text {-}{\mathtt {2j/m}}_{\mathtt {{jj}}}{\mathtt{[350,700]}}$ and ${\mathtt {qqH}}\text {-}{\mathtt {2j/m}}_{\mathtt {{jj}}}{\mathtt{>700}}$, and otherwise if $p_{{\mathrm {T}}} ^{{\mathrm{H}} \text {jj}}>25\,\text {GeV}$ are merged in a single bin called ${\mathtt {qqH}}\text {-}{\mathtt {3j/m}}_{\mathtt {{jj}}}{\mathtt{>350}}$.

The three production processes ${{\mathrm{q}}_{\mathrm{}}^{\mathrm{}}} {{{\overline{{{\mathrm{q}}_{\mathrm{}}^{\mathrm{}}}}}}_{\mathrm{}}^{\mathrm{}}} ^{'}\rightarrow {\mathrm{W}} {\mathrm{H}}$, ${\mathrm{g}} {\mathrm{g}} \rightarrow {\mathrm{Z}} {\mathrm{H}}$, and ${{\mathrm{q}}_{\mathrm{}}^{\mathrm{}}} {{{\overline{{{\mathrm{q}}_{\mathrm{}}^{\mathrm{}}}}}}_{\mathrm{}}^{\mathrm{}}} \rightarrow {\mathrm{Z}} {\mathrm{H}}$ are combined to build ${\mathtt {VH}}\text {-}{\mathtt {lep}}$ reduced stage 1.2 production bins. Several proposed production bins are merged into just two bins according to the $p_{{\mathrm {T}}}$ of the H boson: ${\mathtt {VH}}\text {-}{\mathtt {lep/p}}_{\mathtt {T^{\mathrm{H}}}}{\mathtt{[0,150]}}$ and ${\mathtt {VH}}\text {-}{\mathtt {lep/p}}_{\mathtt {T^{\mathrm{H}}}}{\mathtt{>150}}$.

In stage 1.2 of the STXS framework the ${\mathtt {ttH}}$ stage 0 production bin is split in five different bins according to the $p_{{\mathrm {T}}}$ of the H boson. Because of the very low expected yields all these bins are merged into a single bin that includes the ${{\mathrm{t}}_{\mathrm{}}^{\mathrm{}}} {\mathrm{H}}$ production process as well.

Finally, in stage 1.2 the ${{\mathrm{b}}_{\mathrm{}}^{\mathrm{}}} {}{{{\overline{{{\mathrm{b}}_{\mathrm{}}^{\mathrm{}}}}}}_{\mathrm{}}^{\mathrm{}}} {\mathrm{H}}$ production process, which has small cross section, is classified in the ${\mathtt {ggH}}\text {-}{\mathtt {0j/p}}_{\mathtt {T}}{\mathtt{[10,200]}}$ production bin.

The first measurement of STXS stage 1.2 cross sections was recently performed by the CMS Collaboration [98].

### Reconstructed event categories

In order to be sensitive to different production bins, the ${\mathrm{Z}} {\mathrm{Z}}$ candidates that pass the event selection described in Sect. 4 are classified into several dedicated reconstructed event categories. The category definitions are based on the multiplicity of jets, ${\mathrm{b}}_{\mathrm{}}^{\mathrm{}}$-tagged jets, and additional leptons in the event. Additional leptons are not involved in the ${\mathrm{Z}} {\mathrm{Z}}$ candidate selection but, if present, should satisfy the identification, vertex compatibility, and isolation requirements. Requirements on the categorization discriminants described in Sect. 5, the invariant mass of the two leading jets, and the transverse momentum of the ${\mathrm{Z}} {\mathrm{Z}}$ candidate are also exploited.

The event categorization is carried out in two steps. In the first step, the ${\mathrm{Z}} {\mathrm{Z}}$ candidates are split into seven initial categories to target the main H boson production mechanisms corresponding to the stage 0 production bins. The first step of the categorization closely follows the analysis strategy from the previous publication [18]. To ensure exclusive categories, an event is considered for the subsequent category only if it does not satisfy the requirements of the previous one.

In the first categorization step, the following criteria are applied:

- The ${\mathrm {VBF}}$-2jet-tagged category requires exactly 4 leptons. In addition there must be either 2 or 3 jets of which at most 1 is ${\mathrm{b}}_{\mathrm{}}^{\mathrm{}}$-tagged, or at least 4 jets and no ${\mathrm{b}}_{\mathrm{}}^{\mathrm{}}$-tagged jets. Finally, ${{\mathcal {D}}}_{\text {2jet}}^{{\mathrm {VBF}}} >0.5$ is required.
- The ${{\mathrm{V}}_{\mathrm{}}^{\mathrm{}}} {\mathrm{H}}$-hadronic-tagged category requires exactly 4 leptons. In addition there must be 2 or 3 jets with no ${\mathrm{b}}_{\mathrm{}}^{\mathrm{}}$-tagging requirements, or at least 4 jets and no ${\mathrm{b}}_{\mathrm{}}^{\mathrm{}}$-tagged jets. Finally, ${{\mathcal {D}}}_{\text {2jet}}^{{{\mathrm{V}}_{\mathrm{}}^{\mathrm{}}} {\mathrm{H}}} > 0.5$ is required.
- The ${{\mathrm{V}}_{\mathrm{}}^{\mathrm{}}} {\mathrm{H}}$-leptonic-tagged category requires no more than 3 jets and no ${\mathrm{b}}_{\mathrm{}}^{\mathrm{}}$-tagged jets in the event, and exactly 1 additional lepton or 1 additional pair of opposite sign, same flavor leptons. This category also includes events with no jets and at least 1 additional lepton.
- The ${{{\mathrm{t}}_{\mathrm{}}^{\mathrm{}}} {}{{{\overline{{{\mathrm{t}}_{\mathrm{}}^{\mathrm{}}}}}}_{\mathrm{}}^{\mathrm{}}}} {\mathrm{H}}$-hadronic-tagged category requires at least 4 jets, of which at least 1 is ${\mathrm{b}}_{\mathrm{}}^{\mathrm{}}$-tagged, and no additional leptons.
- The ${{{\mathrm{t}}_{\mathrm{}}^{\mathrm{}}} {}{{{\overline{{{\mathrm{t}}_{\mathrm{}}^{\mathrm{}}}}}}_{\mathrm{}}^{\mathrm{}}}} {\mathrm{H}}$-leptonic-tagged category requires at least 1 additional lepton in the event.
- The ${\mathrm {VBF}}$-1jet-tagged category requires exactly 4 leptons, exactly 1 jet and ${{\mathcal {D}}}_{\text {1jet}}^{{\mathrm {VBF}}} >0.7$.
- The untagged category consists of the remaining events.

Reconstructed events are further subdivided in the second step of the categorization that is designed to closely match the merged stage 1.2 production bins described in the previous section. In the second categorization step, the untagged, ${\mathrm {VBF}}$-2jet-tagged, ${{\mathrm{V}}_{\mathrm{}}^{\mathrm{}}} {\mathrm{H}}$-hadronic-tagged, and ${{\mathrm{V}}_{\mathrm{}}^{\mathrm{}}} {\mathrm{H}}$-leptonic-tagged categories are further split exploiting additional variables like the invariant mass of the two leading jets and the transverse momentum of the ${\mathrm{Z}} {\mathrm{Z}}$ candidate. A total number of twenty-two reconstructed event categories is defined and details of the categorization are presented in Table 2.

**Table 2:** Event categorization criteria of the ${\mathrm{H}} \rightarrow 4\ell$ analysis targeting stage 1.2 STXS production bins. Events from the first step of the categorization are further classified based on the kinematical properties listed in the table. A dash indicates no requirement

Reconstructed event category	1st categorization step	Number of jets	Kinematical requirements ($\,\text {GeV}$)	Targeted production bin
Untagged-0j-$p_{{\mathrm {T}}} ^{4\ell }[0,10]$	Untagged	0	$0<p_{{\mathrm {T}}} ^{4\ell }<10$	${\mathtt {ggH}}\text {-}{\mathtt {0j/p}}_{\mathtt {T}}{\mathtt{[0,10]}}$
Untagged-0j-$p_{{\mathrm {T}}} ^{4\ell }[10,200]$	Untagged	0	$10<p_{{\mathrm {T}}} ^{4\ell }<200$	${\mathtt {ggH}}\text {-}{\mathtt {0j/p}}_{\mathtt {T}}{\mathtt{[10,200]}}$
Untagged-1j-$p_{{\mathrm {T}}} ^{4\ell }[0,60]$	Untagged	1	$0<p_{{\mathrm {T}}} ^{4\ell }<60$	${\mathtt {ggH}}\text {-}{\mathtt {1j/p}}_{\mathtt {T}}{\mathtt{[0,60]}}$
Untagged-1j-$p_{{\mathrm {T}}} ^{4\ell }[60,120]$	Untagged	1	$60<p_{{\mathrm {T}}} ^{4\ell }<120$	${\mathtt {ggH}}\text {-}{\mathtt {1j/p}}_{\mathtt {T}}{\mathtt{[60,120]}}$
Untagged-1j-$p_{{\mathrm {T}}} ^{4\ell }[120,200]$	Untagged	1	$120<p_{{\mathrm {T}}} ^{4\ell }<200$	${\mathtt {ggH}}\text {-}{\mathtt {1j/p}}_{\mathtt {T}}{\mathtt{[120,200]}}$
Untagged-2j-$p_{{\mathrm {T}}} ^{4\ell }[0,60]$	Untagged	2	$0<p_{{\mathrm {T}}} ^{4\ell }<60$, $m_{\text {jj}}<350$	${\mathtt {ggH}}\text {-}{\mathtt {2j/p}}_{\mathtt {T}}{\mathtt{[0,60]}}$
Untagged-2j-$p_{{\mathrm {T}}} ^{4\ell }[60,120]$	Untagged	2	$60<p_{{\mathrm {T}}} ^{4\ell }<120$, $m_{\text {jj}}<350$	${\mathtt {ggH}}\text {-}{\mathtt {2j/p}}_{\mathtt {T}}{\mathtt{[60,120]}}$
Untagged-2j-$p_{{\mathrm {T}}} ^{4\ell }[120,200]$	Untagged	2	$120<p_{{\mathrm {T}}} ^{4\ell }<200$, $m_{\text {jj}}<350$	${\mathtt {ggH}}\text {-}{\mathtt {2j/p}}_{\mathtt {T}}{\mathtt{[120,200]}}$
Untagged-$p_{{\mathrm {T}}} ^{4\ell }>200$	Untagged	–	$p_{{\mathrm {T}}} ^{4\ell }>200$	${\mathtt {ggH/p}}_{\mathtt {T}}{\mathtt{>200}}$
Untagged-2j-$m_{{\mathrm {jj}}}>350$	Untagged	2	$m_{\text {jj}}>350$	${\mathtt {ggH}}\text {-}{\mathtt {2j/m}}_{\mathtt {{jj}}}{\mathtt{>350}}$
${\mathrm {VBF}}$-1jet-tagged	${\mathrm {VBF}}$-1jet-tagged	–	–	${\mathtt {qqH}}\text {-}{\mathtt {rest}}$
${\mathrm {VBF}}$-2jet-tagged-$m_{{\mathrm {jj}}}[350,700]$	${\mathrm {VBF}}$-2jet-tagged	–	$p_{{\mathrm {T}}} ^{4\ell }<200$, $p_{{\mathrm {T}}} ^{4\ell \text {jj}}<25$, $350<m_{\text {jj}}<700$	${\mathtt {qqH}}\text {-}{\mathtt {2j/m}}_{\mathtt {{jj}}}{\mathtt{[350,700]}}$
${\mathrm {VBF}}$-2jet-tagged-$m_{{\mathrm {jj}}}>700$	${\mathrm {VBF}}$-2jet-tagged	–	$p_{{\mathrm {T}}} ^{4\ell }<200$, $p_{{\mathrm {T}}} ^{4\ell \text {jj}}<25$, $m_{\text {jj}}>700$	${\mathtt {qqH}}\text {-}{\mathtt {2j/m}}_{\mathtt {{jj}}}{\mathtt{>700}}$
${\mathrm {VBF}}$-3jet-tagged-$m_{{\mathrm {jj}}}>350$	${\mathrm {VBF}}$-2jet-tagged	–	$p_{{\mathrm {T}}} ^{4\ell }<200$, $p_{{\mathrm {T}}} ^{4\ell \text {jj}}>25$, $m_{\text {jj}}>350$	${\mathtt {qqH}}\text {-}{\mathtt {3j/m}}_{\mathtt {{jj}}}{\mathtt{>350}}$
${\mathrm {VBF}}$-2jet-tagged-$p_{{\mathrm {T}}} ^{4\ell }>200$	${\mathrm {VBF}}$-2jet-tagged	–	$p_{{\mathrm {T}}} ^{4\ell }>200$, $m_{\text {jj}}>350$	${\mathtt {qqH}}\text {-}{\mathtt {2j/p}}_{\mathtt {T}}{\mathtt{>200}}$
${\mathrm {VBF}}$-rest	${\mathrm {VBF}}$-2jet-tagged	–	$m_{\text {jj}}<350$	${\mathtt {qqH}}\text {-}{\mathtt {rest}}$
${{\mathrm{V}}_{\mathrm{}}^{\mathrm{}}} {\mathrm{H}}$-hadronic-tagged-$m_{{\mathrm {jj}}}[60,120]$	${{\mathrm{V}}_{\mathrm{}}^{\mathrm{}}} {\mathrm{H}}$-hadronic-tagged	–	$60<m_{\text {jj}}<120$	${\mathtt {qqH}}\text {-}{\mathtt {2j/m}}_{\mathtt {{jj}}}{\mathtt{[60,120]}}$
${{\mathrm{V}}_{\mathrm{}}^{\mathrm{}}} {\mathrm{H}}$-rest	${{\mathrm{V}}_{\mathrm{}}^{\mathrm{}}} {\mathrm{H}}$-hadronic-tagged	–	$m_{\text {jj}}<60$ or $m_{\text {jj}}>120$	${\mathtt {qqH}}\text {-}{\mathtt {rest}}$
${{\mathrm{V}}_{\mathrm{}}^{\mathrm{}}} {\mathrm{H}}$-leptonic-tagged-$p_{{\mathrm {T}}} ^{4\ell }[0,150]$	${{\mathrm{V}}_{\mathrm{}}^{\mathrm{}}} {\mathrm{H}}$-leptonic-tagged	–	$p_{{\mathrm {T}}} ^{4\ell }<150$	${\mathtt {VH}}\text {-}{\mathtt {lep/p}}_{\mathtt {T^{\mathrm{H}}}}{\mathtt{[0,150]}}$
${{\mathrm{V}}_{\mathrm{}}^{\mathrm{}}} {\mathrm{H}}$-leptonic-tagged-$p_{{\mathrm {T}}} ^{4\ell }>150$	${{\mathrm{V}}_{\mathrm{}}^{\mathrm{}}} {\mathrm{H}}$-leptonic-tagged	–	$p_{{\mathrm {T}}} ^{4\ell }>150$	${\mathtt {VH}}\text {-}{\mathtt {lep/p}}_{\mathtt {T^{\mathrm{H}}}}{\mathtt{>150}}$
${{{\mathrm{t}}_{\mathrm{}}^{\mathrm{}}} {}{{{\overline{{{\mathrm{t}}_{\mathrm{}}^{\mathrm{}}}}}}_{\mathrm{}}^{\mathrm{}}}} {\mathrm{H}}$-leptonic-tagged	${{{\mathrm{t}}_{\mathrm{}}^{\mathrm{}}} {}{{{\overline{{{\mathrm{t}}_{\mathrm{}}^{\mathrm{}}}}}}_{\mathrm{}}^{\mathrm{}}}} {\mathrm{H}}$-leptonic-tagged	–	–	${\mathtt {ttH}}$
${{{\mathrm{t}}_{\mathrm{}}^{\mathrm{}}} {}{{{\overline{{{\mathrm{t}}_{\mathrm{}}^{\mathrm{}}}}}}_{\mathrm{}}^{\mathrm{}}}} {\mathrm{H}}$-hadronic-tagged	${{{\mathrm{t}}_{\mathrm{}}^{\mathrm{}}} {}{{{\overline{{{\mathrm{t}}_{\mathrm{}}^{\mathrm{}}}}}}_{\mathrm{}}^{\mathrm{}}}} {\mathrm{H}}$-hadronic-tagged	–	–	${\mathtt {ttH}}$

## Background estimation

### Irreducible backgrounds

The irreducible background to the H boson signal in the $4\ell$ channel, which comes from the production of ${\mathrm{Z}} {\mathrm{Z}}$ via ${{\mathrm{q}}_{\mathrm{}}^{\mathrm{}}} {{{\overline{{{\mathrm{q}}_{\mathrm{}}^{\mathrm{}}}}}}_{\mathrm{}}^{\mathrm{}}}$ annihilation or gluon fusion, is estimated using simulation. The fully differential cross section for the ${{\mathrm{q}}_{\mathrm{}}^{\mathrm{}}} {{{\overline{{{\mathrm{q}}_{\mathrm{}}^{\mathrm{}}}}}}_{\mathrm{}}^{\mathrm{}}} \rightarrow {\mathrm{Z}} {\mathrm{Z}}$ process is computed at NNLO [99], and the NNLO/NLO K factor as a function of $m_{{\mathrm{Z}} {\mathrm{Z}}}$ is applied to the powheg sample. This K factor varies from 1.0 to 1.2 and is 1.1 at $m_{{\mathrm{Z}} {\mathrm{Z}}}=125\,\text {GeV}$. Additional NLO electroweak corrections that depend on the initial state quark flavor and kinematics are also applied in the region $m_{{\mathrm{Z}} {\mathrm{Z}}}>2m_{{\mathrm{Z}}}$ following the prescription in Ref. [100].

The production of ${\mathrm{Z}} {\mathrm{Z}}$ via gluon fusion contributes at NNLO in pQCD. It has been shown that the soft collinear approximation is able to describe the cross section for this process and the interference term at NNLO [101]. Further calculations also show that the K factors are very similar at NLO for signal and background [102] and at NNLO for signal and interference terms [103]. Therefore, the same K factor is used for signal and background [104]. The NNLO K factor for the signal is obtained as a function of $m_{{\mathrm{Z}} {\mathrm{Z}}}$ using the hnnlo v2 program [105–107] by calculating the NNLO and LO ${\mathrm{g}} {\mathrm{g}} \rightarrow {\mathrm{H}} \rightarrow 2\ell 2\ell ^\prime$ cross sections for the ${\mathrm{H}}$ boson decay width of 4.07$\,\text {Me}\text {V}$ and taking their ratios. The NNLO/LO K factor for ${\mathrm{g}} {\mathrm{g}} \rightarrow {\mathrm{Z}} {\mathrm{Z}}$ varies from $\approx$2.0 to 2.6 and is 2.27 at $m_{{\mathrm{Z}} {\mathrm{Z}}}=125\,\text {GeV}$; a systematic uncertainty of 10% is assigned to it when applied to the background process.

The triboson background processes ${\mathrm{Z}} {\mathrm{Z}} {\mathrm{Z}}$, ${\mathrm{W}} {\mathrm{Z}} {\mathrm{Z}}$, and ${\mathrm{W}} {\mathrm{W}} {\mathrm{Z}}$, as well as ${{{\mathrm{t}}_{\mathrm{}}^{\mathrm{}}} {}{{{\overline{{{\mathrm{t}}_{\mathrm{}}^{\mathrm{}}}}}}_{\mathrm{}}^{\mathrm{}}}} {\mathrm{Z}}$, ${{{\mathrm{t}}_{\mathrm{}}^{\mathrm{}}} {}{{{\overline{{{\mathrm{t}}_{\mathrm{}}^{\mathrm{}}}}}}_{\mathrm{}}^{\mathrm{}}}} {\mathrm{W}} {\mathrm{W}}$, and ${{{\mathrm{t}}_{\mathrm{}}^{\mathrm{}}} {}{{{\overline{{{\mathrm{t}}_{\mathrm{}}^{\mathrm{}}}}}}_{\mathrm{}}^{\mathrm{}}}} {\mathrm{Z}} {\mathrm{Z}}$ are also considered. These rare backgrounds are all estimated from simulation and are jointly referred to as the EW backgrounds.

Simulated samples are used to obtain shapes of the four-lepton invariant mass that are later used to build the likelihood function. For each irreducible background contribution, events are divided in three final states ($4{{{\upmu }}_{\mathrm{}}^{\mathrm{}}}$, $4{\mathrm{e}}$, and $2{\mathrm{e}} 2{{{\upmu }}_{\mathrm{}}^{\mathrm{}}}$) and 22 event sub-categories defined in Sect. 6.1. To extract the shape of the $m_{4\ell }$ distribution, expected yields are fitted to empirical functional forms built from a third order Bernstein polynomial. In sub-categories with not enough statistics to perform a fit, the shape is extracted from the inclusive distribution in the corresponding final state.

### Reducible backgrounds

Additional backgrounds to the H boson signal in the $4\ell$ channel arise from processes in which decays of heavy-flavor hadrons, in-flight decays of light mesons within jets, or (for electrons) charged hadrons overlapping with $\pi ^0$ decays are misidentified as leptons. The main processes leading to these backgrounds are ${\mathrm{Z}} \text {+jets}$, ${{{\mathrm{t}}_{\mathrm{}}^{\mathrm{}}} {}{{{\overline{{{\mathrm{t}}_{\mathrm{}}^{\mathrm{}}}}}}_{\mathrm{}}^{\mathrm{}}}} \text {+jets}$, ${\mathrm{Z}} \gamma \text {+jets}$, ${\mathrm{W}} {\mathrm{W}} \text {+jets}$, and ${\mathrm{W}} {\mathrm{Z}} \text {+jets}$ production. We denote these reducible backgrounds as ”${\mathrm{Z}} \text {+X}$” since they are dominated by the ${\mathrm{Z}} \text {+jets}$ process. The contribution from the reducible background is estimated with two independent methods, each with dedicated control regions in data. The control regions are defined by the presence of both a lepton pair satisfying all the requirements of a ${\mathrm{Z}} _1$ candidate and two additional opposite sign (OS) or same sign (SS) leptons; the two additional leptons satisfy identification requirements looser than those used in the analysis. These four leptons are then required to pass the analysis ${\mathrm{Z}} {\mathrm{Z}}$ candidate selection. The event yield in the signal region is obtained by weighting the control region events by the lepton misidentification probability $f_{{\mathrm{e}}}$ ($f_{{{{\upmu }}_{\mathrm{}}^{\mathrm{}}}}$), defined as the fraction of non-signal electrons (muons) that are identified by the analysis selection criteria. A detailed description of both methods can be found in Ref. [18].

The lepton misidentification rates $f_{{\mathrm{e}}}$ and $f_{{{{\upmu }}_{\mathrm{}}^{\mathrm{}}}}$ are measured as a function of $p_{{\mathrm {T}}} ^\ell$ and $\eta ^\ell$ by means of a sample that includes a ${\mathrm{Z}} _1$ candidate consisting of a pair of leptons, both passing the selection requirements used in the analysis, and exactly one additional lepton passing the relaxed selection. Furthermore, the $p_{{\mathrm {T}}} ^\text {miss}$ is required to be less than 25$\,\text {GeV}$ in order to suppress contamination from ${\mathrm{W}} {\mathrm{Z}}$ and ${{{\mathrm{t}}_{\mathrm{}}^{\mathrm{}}} {}{{{\overline{{{\mathrm{t}}_{\mathrm{}}^{\mathrm{}}}}}}_{\mathrm{}}^{\mathrm{}}}}$ processes.

For the OS method, the mass of the ${\mathrm{Z}} _1$ candidate is required to satisfy the condition $|{\mathrm{Z}} _1 - m_{{\mathrm{Z}}} | < 7\,\text {GeV}$ in order to reduce the contribution of (asymmetric) photon conversions, which is estimated separately. In the SS method, the contribution of photon conversions to the misidentification rate is estimated with dedicated samples.

The predicted yields of the reducible background from the two methods are in agreement within their uncertainties for each final state ($4{{{\upmu }}_{\mathrm{}}^{\mathrm{}}}$, $4{\mathrm{e}}$, and $2{\mathrm{e}} 2{{{\upmu }}_{\mathrm{}}^{\mathrm{}}}$). The final yield used in the analysis is a weighted average of the two independent estimates. To extract the shape of the $m_{4\ell }$ distribution for the reducible background a maximum-likelihood fit is performed in each of the 22 event sub-categories defined in Sect. 6.1. For each sub-category, the expected ”${\mathrm{Z}} \text {+X}$” yields from the OS and SS methods are binned as a function of $m_{4\ell }$ and fitted to empirical functional forms built from Landau distributions [108]. In sub-categories with not enough statistics to perform a fit, the shape is extracted from the inclusive distribution in the corresponding final state.

The dominant systematic uncertainty on the reducible background estimation arises from the difference in the composition of the sample from which the misidentification rate is computed and that of the control regions. It is determined from the MC simulation and is found to be around 30%, depending on the final state. Additional sources of systematic uncertainty arise from the limited number of events in the control regions as well as in the region where the misidentification rates are computed.

## Signal modeling

In order to generate an accurate signal model, the $p_{{\mathrm {T}}}$ spectrum of the H boson, $p_{{\mathrm {T}}} ^{{\mathrm{H}}}$, was tuned in the powheg simulation of the dominant gluon fusion production mode to better match the predictions from full phase space calculations implemented in the HRes generator [107, 109, 110].

In order to take advantage of the accuracy of the nnlops [111] simulation available for the ${\mathrm{g}} {\mathrm{g}} {\mathrm{H}}$ process, an event reweighting procedure is used. Events originating from the ${\mathrm{g}} {\mathrm{g}} {\mathrm{H}}$ process are subdivided into classes with 0, 1, 2, and $\ge$3 jets; the jets with $p_{{\mathrm {T}}} > 30\,\text {GeV}$ are clustered from all stable particles using the anti-$k_{{\mathrm {T}}}$ algorithm with a distance parameter of 0.4, excluding the decay products of the H boson or associated vector bosons. The weights are obtained as the ratios of the $p_{{\mathrm {T}}} ^{{\mathrm{H}}}$ distributions from the nnlops and the powheg generators for each event class; the sum of the weights in each sample is normalized to the inclusive cross section.

**Fig. 2:**
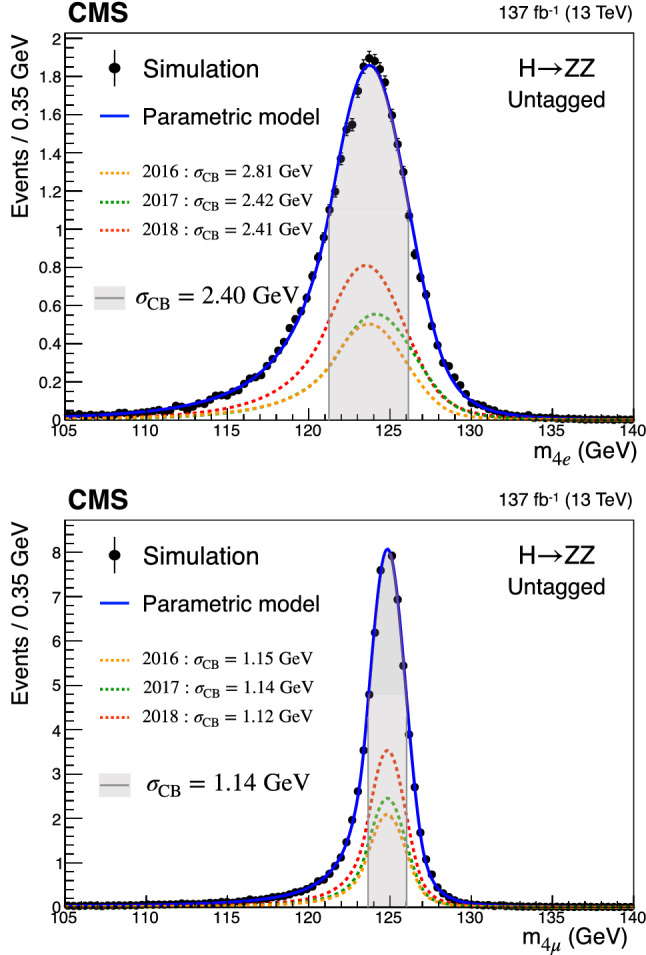
The shape of the parametric signal model for each year of simulated data, and for the sum of all years together. The black points represent weighted simulation events of the ${\mathrm{g}} {\mathrm{g}} {\mathrm{H}}$ production mechanism for $m_{{\mathrm{H}}}=125\,\text {GeV}$ and the blue line the corresponding model. Also shown is the $\sigma _{\text {CB}}$ value (half the width of the narrowest interval containing 68% of the invariant mass distribution) in the gray shaded area. The contribution of the signal model from each year of data-taking is illustrated with the dotted lines. The models are shown for the $4{\mathrm{e}}$ (upper) and $4{{{\upmu }}_{\mathrm{}}^{\mathrm{}}}$ (lower) final states in the untagged event category

The signal shape is parametrized by means of a double-sided Crystal Ball function [112] around $m_{{\mathrm{H}}}\approx 125\,\text {GeV}$. A Landau function is added in the total probability density function for the non-resonant part of the signal for the case of ${\mathrm{W}} {\mathrm{H}}$, ${\mathrm{Z}} {\mathrm{H}}$ and ${{{\mathrm{t}}_{\mathrm{}}^{\mathrm{}}} {}{{{\overline{{{\mathrm{t}}_{\mathrm{}}^{\mathrm{}}}}}}_{\mathrm{}}^{\mathrm{}}}} {\mathrm{H}}$ production modes. The signal shape is parametrized as a function of $m_{{\mathrm{H}}}$ by performing a simultaneous fit of several mass points for all production modes in the 105 to 140$\,\text {GeV}$ mass range. Each parameter of the double-sided Crystal Ball function has a linear dependence on $m_{{\mathrm{H}}}$, for a total of 12 free parameters. An examples of the fit is shown in Fig. 2.

## Systematic uncertainties

The systematic uncertainties are divided into experimental and theoretical. The main experimental uncertainties originate from the imperfect knowledge of the detector; the dominant sources are the uncertainties in the luminosity measurement, the lepton reconstruction and selection efficiency, the lepton and jet energy scale and resolution, the ${\mathrm{b}}_{\mathrm{}}^{\mathrm{}}$ tagging efficiency, and the reducible background estimate. The theoretical uncertainties account for the uncertainties in the modeling of the signal and background processes.

Both types of uncertainties can affect the signal selection, cause migrations between the event categories, and affect the signal or background shapes used in the fit. All the uncertainties affecting this analysis are modeled as nuisance parameters (NPs) that are profiled in the maximum likelihood fit described in Sect. 10.

**Fig. 3:**
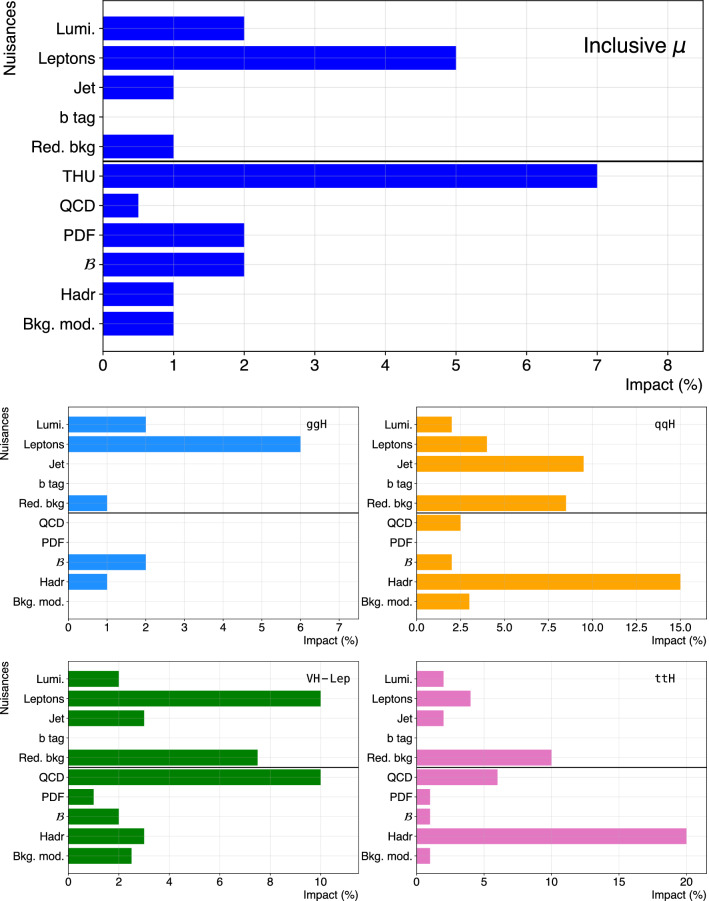
The impact of the dominant systematic uncertainties (in percent) on the inclusive signal strength $\mu$ and stage 0 production mode cross section described in Sect. 10. Impacts from different NPs are combined assuming no correlation. Only dominant experimental sources are presented: integrated luminosity uncertainty (Lumi.), lepton reconstruction and selection efficiency, scale and resolution (Leptons), jet energy scale and resolution (Jet), ${\mathrm{b}}_{\mathrm{}}^{\mathrm{}}$-tagging efficiency (B-tag), and reducible background estimation uncertainty (Red. bkg). Only dominant theoretical sources are presented: ${\mathrm{g}} {\mathrm{g}} {\mathrm{H}}$, ${\mathrm {VBF}}$, and ${{\mathrm{V}}_{\mathrm{}}^{\mathrm{}}} {\mathrm{H}}$ cross section theoretical uncertainty scheme (THU), renormalization and factorization scale (QCD), choice of the PDF set (PDF), the branching fraction of ${\mathrm{H}} \rightarrow 4\ell$ (${\mathcal {B}}$), modeling of hadronization and the underlying event (Hadr), and background modeling (Bkg. mod.). The THU uncertainty is not considered in the stage 0 cross section measurements. The uncertainties are rounded to the nearest 0.5%

**Fig. 4:**
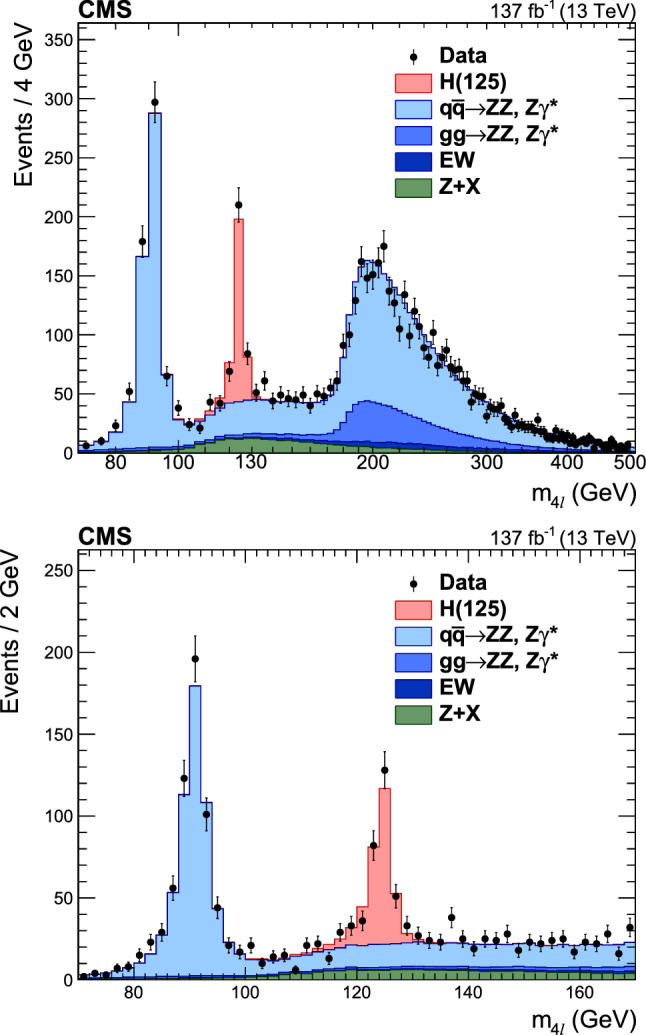
Four-lepton mass distribution, $m_{4\ell }$, up to $500\,\text {GeV}$ with $4\,\text {GeV}$ bin size (upper) and in the low-mass range with $2\,\text {GeV}$ bin size (lower). Points with error bars represent the data and stacked histograms represent the expected distributions for the signal and background processes. The SM Higgs boson signal with $m_{{\mathrm{H}}}=125\,\text {GeV}$, denoted as ${\mathrm{H}} (125)$, the ${\mathrm{Z}} {\mathrm{Z}}$ and rare electroweak backgrounds are normalized to the SM expectation, the ${\mathrm{Z}}$+X background to the estimation from data

**Fig. 5:**
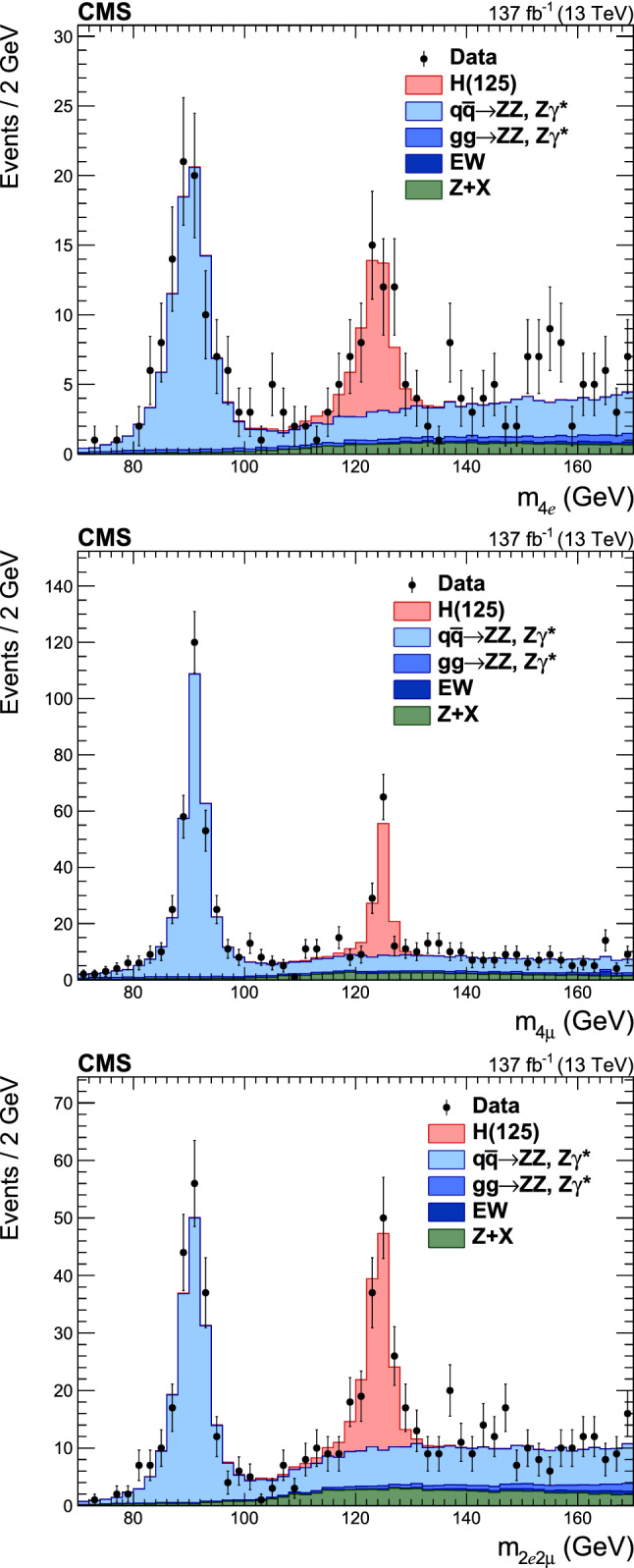
Four-lepton mass distribution in three final states: $4{\mathrm{e}}$ upper), $4{{{\upmu }}_{\mathrm{}}^{\mathrm{}}}$ (center), and $2{\mathrm{e}} 2{{{\upmu }}_{\mathrm{}}^{\mathrm{}}}$ (lower). Points with error bars represent the data and stacked histograms represent the expected distributions for the signal and background processes. The SM Higgs boson signal with $m_{{\mathrm{H}}}=125\,\text {GeV}$, denoted as ${\mathrm{H}} (125)$, the ${\mathrm{Z}} {\mathrm{Z}}$ and rare electroweak backgrounds are normalized to the SM expectation, the ${\mathrm{Z}}$+X background to the estimation from data

In the combination of the three data-taking periods, all theoretical uncertainties are treated as correlated across these periods. The experimental uncertainties related to reconstruction and selection efficiency, the lepton energy scale and resolution, and the ${\mathrm{b}}_{\mathrm{}}^{\mathrm{}}$-tagging efficiency are also considered correlated across data-taking periods. Luminosity uncertainty is treated as partially correlated. All other experimental uncertainties are treated as uncorrelated. Correlated sources of uncertainty are assigned the same NP and uncorrelated sources have a dedicated NP in the likelihood fit described in Sect. 10.

The dominant sources of uncertainties and their effect on the analysis are discussed in detail in the following subsections. The impact of a NP on a parameter of interest (POI) is defined as the shift induced on POI when NP is varied by a $\pm 1$ standard deviation from its post-fit value, with all other parameters profiled as usual. The relative impact of the dominant systematic uncertainties on some of the measurements discussed in Sect. 10 is illustrated in Fig. 3.

### Experimental uncertainties

**Table 3:** Number of expected background and signal events and number of observed candidates after full analysis selection, for each event category, in the mass range $105<m_{4\ell }<140\,\text {GeV}$ and for an integrated luminosity of $137{\,\text {fb}^{-1}}$. The yields are given for the different production modes. The uncertainties listed are statistical only. Signal is estimated from MC simulation at $m_{{\mathrm{H}}}=125\,\text {GeV}$, ${\mathrm{Z}} {\mathrm{Z}}$ and rare electroweak backgrounds are also estimated from MC simulation, and ${\mathrm{Z}}$+X is estimated from data

Reconstructed event category	Signal							Background				Expected		Observed
	${\mathrm{g}} {\mathrm{g}} {\mathrm{H}}$	${\mathrm {VBF}}$	${\mathrm{W}} {\mathrm{H}}$	${\mathrm{Z}} {\mathrm{H}}$	${{{\mathrm{t}}_{\mathrm{}}^{\mathrm{}}} {}{{{\overline{{{\mathrm{t}}_{\mathrm{}}^{\mathrm{}}}}}}_{\mathrm{}}^{\mathrm{}}}} {\mathrm{H}}$	${{\mathrm{b}}_{\mathrm{}}^{\mathrm{}}} {}{{{\overline{{{\mathrm{b}}_{\mathrm{}}^{\mathrm{}}}}}}_{\mathrm{}}^{\mathrm{}}} {\mathrm{H}}$	${{\mathrm{t}}_{\mathrm{}}^{\mathrm{}}} {\mathrm{H}}$	${{\mathrm{q}}_{\mathrm{}}^{\mathrm{}}} {{{\overline{{{\mathrm{q}}_{\mathrm{}}^{\mathrm{}}}}}}_{\mathrm{}}^{\mathrm{}}} \rightarrow {\mathrm{Z}} {\mathrm{Z}}$	${\mathrm{g}} {\mathrm{g}} \rightarrow {\mathrm{Z}} {\mathrm{Z}}$	EW	${\mathrm{Z}}$+X	Signal	Total	
Untagged-0j-$p_{{\mathrm {T}}} ^{4\ell }[0,10]$	27.7	0.09	0.03	0.03	0.00	0.15	0.00	71.5	3.06	0.01	3.21	27.9 ± 0.1	106 ± 0	114
Untagged-0j-$p_{{\mathrm {T}}} ^{4\ell }[10,200]$	96.2	1.69	0.60	0.77	0.01	1.01	0.00	98.1	11.6	0.35	37.8	100 ± 0	248 ± 1	278
Untagged-1j-$p_{{\mathrm {T}}} ^{4\ell }[0,60]$	26.8	1.51	0.56	0.48	0.01	0.45	0.01	25.3	3.02	0.64	14.2	29.8 ± 0.1	72.9 ± 0.4	74
Untagged-1j-$p_{{\mathrm {T}}} ^{4\ell }[60,120]$	13.5	1.31	0.51	0.41	0.02	0.11	0.01	7.81	0.82	0.62	7.95	15.9 ± 0.1	33.1 ± 0.3	20
Untagged-1j-$p_{{\mathrm {T}}} ^{4\ell }[120,200]$	3.51	0.60	0.17	0.17	0.01	0.02	0.00	1.15	0.19	0.25	1.63	4.48 ± 0.05	7.69 ± 0.16	11
Untagged-2j-$p_{{\mathrm {T}}} ^{4\ell }[0,60]$	3.45	0.29	0.15	0.14	0.08	0.09	0.02	2.14	0.32	0.63	4.75	4.20 ± 0.06	12.1 ± 0.2	14
Untagged-2j-$p_{{\mathrm {T}}} ^{4\ell }[60,120]$	5.26	0.56	0.24	0.19	0.12	0.04	0.03	2.19	0.30	0.72	4.14	6.43 ± 0.06	13.8 ± 0.2	15
Untagged-2j-$p_{{\mathrm {T}}} ^{4\ell }[120,200]$	3.07	0.40	0.16	0.13	0.07	0.01	0.02	0.75	0.14	0.34	1.19	3.86 ± 0.05	6.28 ± 0.14	7
Untagged-$p_{{\mathrm {T}}} ^{4\ell }>200$	2.79	0.62	0.21	0.17	0.07	0.01	0.02	0.43	0.21	0.21	0.73	3.89 ± 0.04	5.47 ± 0.11	3
Untagged-2j-$m_{\text {jj}}>350$	0.77	0.16	0.06	0.04	0.05	0.01	0.01	0.34	0.06	0.31	1.71	1.12 ± 0.02	3.54 ± 0.14	3
${\mathrm {VBF}}$-1jet-tagged	15.5	3.29	0.22	0.16	0.00	0.13	0.01	6.85	1.53	0.20	2.44	19.3 ± 0.1	30.3 ± 0.2	27
${\mathrm {VBF}}$-2jet-tagged-$m_{\text {jj}}[350,700]$	0.83	1.19	0.01	0.01	0.00	0.01	0.00	0.19	0.07	0.11	0.14	2.05 ± 0.03	2.55 ± 0.05	2
${\mathrm {VBF}}$-2jet-tagged-$m_{\text {jj}}>700$	0.43	1.96	0.00	0.00	0.00	0.00	0.00	0.07	0.05	0.12	0.03	2.40 ± 0.02	2.67 ± 0.03	1
${\mathrm {VBF}}$-3jet-tagged-$m_{\text {jj}}>350$	2.52	2.35	0.06	0.06	0.03	0.03	0.05	0.62	0.21	0.64	2.43	5.11 ± 0.05	9.01 ± 0.17	12
${\mathrm {VBF}}$-2jet-tagged-$p_{{\mathrm {T}}} ^{4\ell }>200$	0.44	0.79	0.01	0.01	0.01	0.00	0.01	0.03	0.03	0.04	0.06	1.26 ± 0.02	1.42 ± 0.03	0
VBF-rest	2.48	0.94	0.13	0.09	0.04	0.04	0.01	0.98	0.20	0.39	2.18	3.74 ± 0.05	7.49 ± 0.17	5
${{\mathrm{V}}_{\mathrm{}}^{\mathrm{}}} {\mathrm{H}}$-hadronic-tagged-$m_{\text {jj}}[60,120]$	4.11	0.25	1.09	0.96	0.13	0.06	0.02	1.69	0.22	0.52	2.93	6.62 ± 0.06	12.0 ± 0.2	12
VH-rest	0.57	0.03	0.09	0.06	0.03	0.01	0.00	0.16	0.02	0.06	0.33	0.79 ± 0.02	1.36 ± 0.06	0
${{\mathrm{V}}_{\mathrm{}}^{\mathrm{}}} {\mathrm{H}}$-leptonic-tagged-$p_{{\mathrm {T}}} ^{4\ell }[0,150]$	0.33	0.04	0.85	0.26	0.10	0.03	0.03	2.16	0.36	0.19	1.11	1.64 ± 0.02	5.47 ± 0.13	10
${{\mathrm{V}}_{\mathrm{}}^{\mathrm{}}} {\mathrm{H}}$-leptonic-tagged-$p_{{\mathrm {T}}} ^{4\ell }>150$	0.02	0.01	0.21	0.06	0.04	0.00	0.01	0.05	0.01	0.03	0.08	0.35 ± 0.01	0.52 ± 0.03	0
${{{\mathrm{t}}_{\mathrm{}}^{\mathrm{}}} {}{{{\overline{{{\mathrm{t}}_{\mathrm{}}^{\mathrm{}}}}}}_{\mathrm{}}^{\mathrm{}}}} {\mathrm{H}}$-leptonic-tagged	0.02	0.01	0.02	0.02	0.68	0.00	0.03	0.08	0.01	0.23	0.21	0.79 ± 0.01	1.32 ± 0.07	0
${{{\mathrm{t}}_{\mathrm{}}^{\mathrm{}}} {}{{{\overline{{{\mathrm{t}}_{\mathrm{}}^{\mathrm{}}}}}}_{\mathrm{}}^{\mathrm{}}}} {\mathrm{H}}$-hadronic-tagged	0.18	0.05	0.03	0.05	0.86	0.01	0.03	0.03	0.01	0.82	1.06	1.22 ± 0.01	3.15 ± 0.14	2

**Fig. 6:**
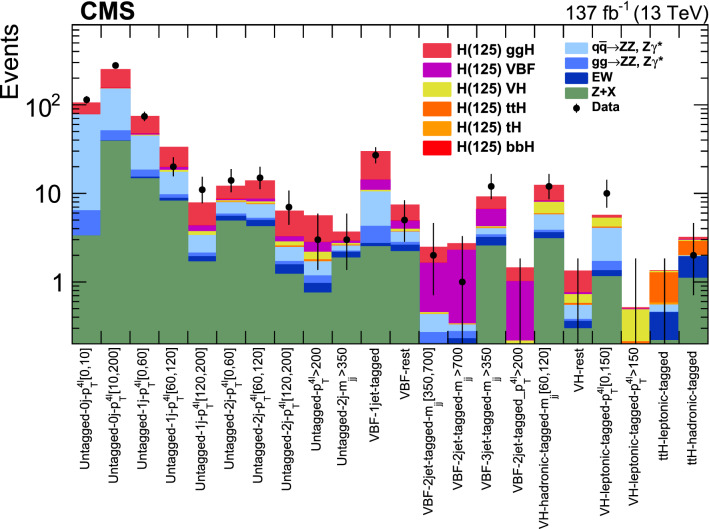
Distributions of the expected and observed number of events for the reconstructed event categories in the mass region $105<m_{4\ell }<140\,\text {GeV}$. Points with error bars represent the data and stacked histograms represent the expected numbers of the signal and background events. The yields of the different H boson production mechanisms with $m_{{\mathrm{H}}}=125\,\text {GeV}$, denoted as ${\mathrm{H}} (125)$, and those of the ${\mathrm{Z}} {\mathrm{Z}}$ and rare electroweak backgrounds are normalized to the SM expectations, while the ${\mathrm{Z}}$+X background yield is normalized to the estimate from the data

**Fig. 7:**
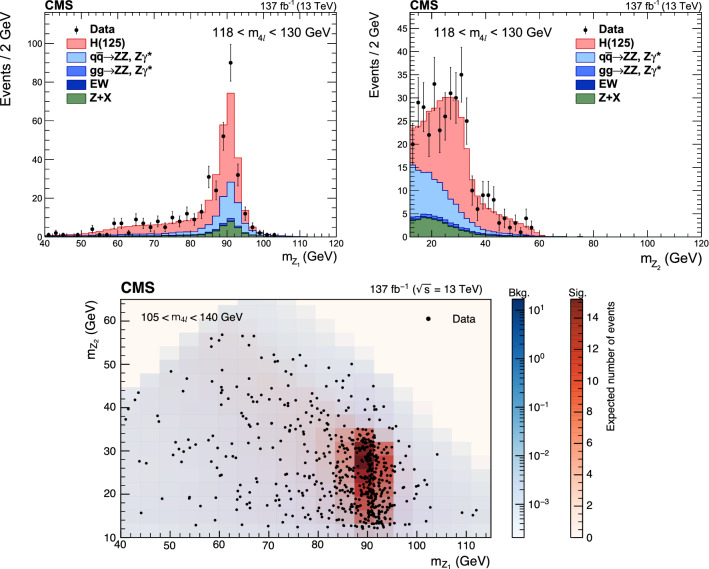
Distribution of the ${\mathrm{Z}} _1$ (upper left) and ${\mathrm{Z}} _2$ (upper right) reconstructed masses in the $118<m_{4\ell }<130\,\text {GeV}$ mass region and their 2D distribution (lower) in the $105<m_{4\ell }<140\,\text {GeV}$ mass region. The stacked histograms and the red and blue scales represent expected distributions of the signal and background processes and the points represent the data. The yields of the different H boson production mechanisms with $m_{{\mathrm{H}}}=125\,\text {GeV}$, denoted as ${\mathrm{H}} (125)$, and those of the ${\mathrm{Z}} {\mathrm{Z}}$ and rare electroweak backgrounds are normalized to the SM expectations, while the ${\mathrm{Z}}$+X background yield is normalized to the estimate from the data

The integrated luminosities of the 2016, 2017, and 2018 data-taking periods are individually known with uncertainties in the 2.3–2.5% range [41–43], while the total Run 2 (2016–2018) integrated luminosity has an uncertainty of 1.8%, the improvement in precision reflecting the (uncorrelated) time evolution of some systematic effects. The experimental uncertainty on the integrated luminosity measurement affects all final states, both signal and background. Another experimental uncertainty common to all final states is the uncertainty in the lepton reconstruction and selection efficiency. Here selection efficiency includes all the steps from trigger to impact parameter significance and finally identification and isolation requirements. The uncertainty ranges from 1 to 2.3% in the 4${{\upmu }}_{\mathrm{}}^{\mathrm{}}$ channel and from 11 to 15.5% in the 4e channel. While for muon efficiency measurements in the low $p_{{\mathrm {T}}} ^{{{{\upmu }}_{\mathrm{}}^{\mathrm{}}}}$ regions we rely on low mass di-muon resonances, the electron efficiency measurement relies solely on the Z boson resonance, resulting in a higher uncertainty in the low $p_{{\mathrm {T}}} ^{{\mathrm{e}}}$ region.

Lepton momentum scale and resolution uncertainties are estimated from dedicated studies on the ${\mathrm{Z}} \rightarrow \ell ^{+}\ell ^{-}$ mass distribution in data and simulation. Events are classified according to the $p_{{\mathrm {T}}}$ and $\eta$ of one of the two leptons, determined randomly, and integrated over the other. The dilepton mass distributions are then fit by a Breit–Wigner parameterization convolved with the double-sided Crystal Ball function described in Sect. 8. The scale uncertainty is found to be 0.04% in the 4${{\upmu }}_{\mathrm{}}^{\mathrm{}}$ channel and 0.3% in the 4e channel, while the resolution uncertainty is 20% for both channels. In both cases full correlation between the leptons in the event is assumed. Both scale and resolution uncertainties alter the signal shape by allowing the corresponding parameters of the double-sided Crystal Ball function to vary. The impact is found to be non-negligible only in the case of fiducial cross section measurements.

The effects of the jet energy corrections are studied in a similar manner. While jet energy scale and smearing do not alter signal selection efficiency, they cause event migrations between the categories. They can also alter the shape of the discriminants, but the effect on the shape is negligible. The uncertainty in the jet energy scale ranges from 1% in the high jet $p_{{\mathrm {T}}}$ range and increases up to 5% in the low jet $p_{{\mathrm {T}}}$ range. The uncertainty in jet energy resolution ranges from 1 to 2%. A detailed description of the determination of the jet energy scale and smearing uncertainties can be found in [113]. The effect on the analysis is studied in detail by propagating the uncertainties and estimating the effect on event migration in each of the 22 sub-categories. Their impact on the inclusive measurements is found to be negligible. However, the impact is significant in measurements of the ${\mathrm {VBF}}$ and ${{\mathrm{V}}_{\mathrm{}}^{\mathrm{}}} {\mathrm{H}}$ production modes and differential cross section measurements as a function of jet kinematics, where it is one of the leading sources of uncertainty.

**Fig. 8:**
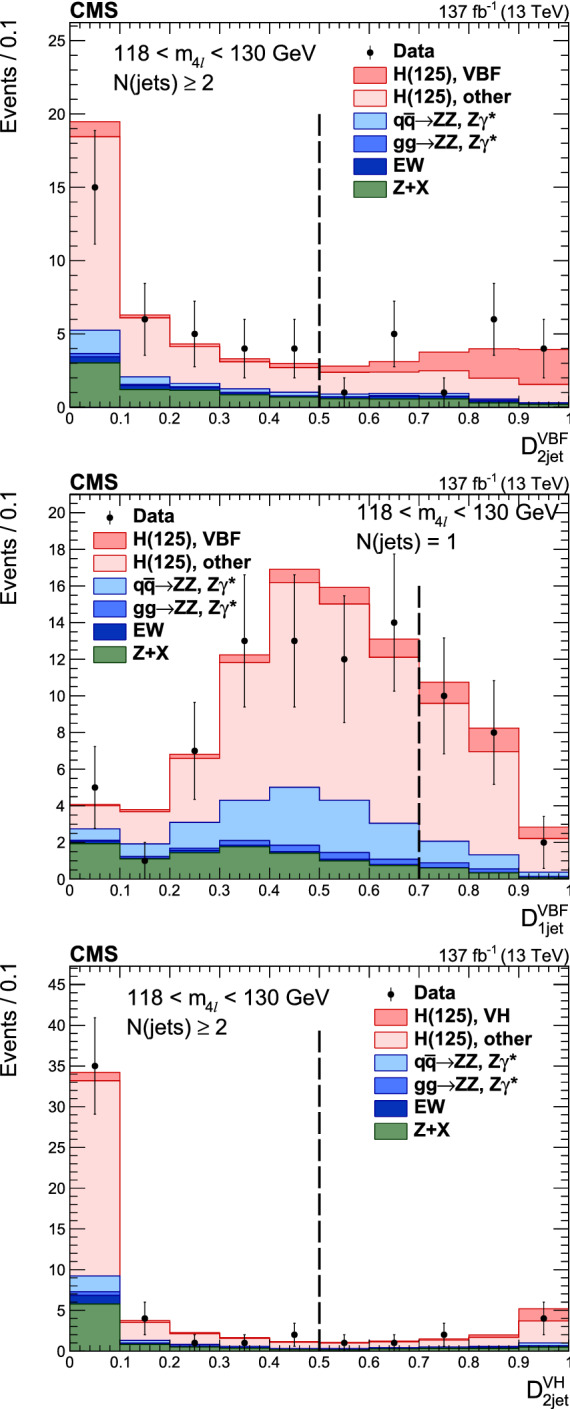
Distribution of categorization discriminants in the mass region $118<m_{4\ell }<130\,\text {GeV}$: ${{\mathcal {D}}}_{\text {2jet}}^{{\mathrm {VBF}}}$ (upper), ${{\mathcal {D}}}_{\text {1jet}}^{{\mathrm {VBF}}}$ (center), ${{\mathcal {D}}}_{\text {2jet}}^{{{\mathrm{V}}_{\mathrm{}}^{\mathrm{}}} {\mathrm{H}}}$ (lower) = max(${{\mathcal {D}}}_{\text {2jet}}^{{\mathrm{W}} {\mathrm{H}}}$,${{\mathcal {D}}}_{\text {2jet}}^{{\mathrm{Z}} {\mathrm{H}}}$). Points with error bars represent the data and stacked histograms represent expected distributions of the signal and background processes. The SM Higgs boson signal with $m_{{\mathrm{H}}}=125\,\text {GeV}$, denoted as ${\mathrm{H}} (125)$, and the ${\mathrm{Z}} {\mathrm{Z}}$ backgrounds and rare electroweak backgrounds are normalized to the SM expectation, the ${\mathrm{Z}}$+X background to the estimation from data. The vertical dashed lines denote the working points used in the event categorization. The SM H boson signal is separated into two components: the production mode which is targeted by the specific discriminant, and other production modes, where the gluon fusion process dominates.

The uncertainty in the ${\mathrm{b}}_{\mathrm{}}^{\mathrm{}}$-tagging efficiency is found to be 1% in the high jet $p_{{\mathrm {T}}}$ range and increases up to 3% in the low jet $p_{{\mathrm {T}}}$ range. The impact from the category migration is found to be negligible in all categories.

Finally, experimental uncertainties in the reducible background estimation, described in Sect. 7.2, originating from the background composition and misidentification rate uncertainties vary between 30 and 45% depending on the final state and category. However, the impact of this uncertainty on the measurements is found to be negligible.

Other sources of experimental uncertainties are also studied but their impact is negligible compared to the sources described above.

### Theoretical uncertainties

Theoretical uncertainties that affect both the signal and background estimation include those related to the renormalization and factorization scales, and the choice of the PDF set. The uncertainty from the renormalization and factorization scales is determined by varying these scales between 0.5 and 2 times their nominal value, while keeping their ratio between 0.5 and 2. The uncertainty due to the PDF set is determined following the PDF4LHC recommendations by taking the root mean square of the variation of the results when using different replicas of the default NNPDF set [114, 115]. The uncertainties just described have an effect both on the signal and background yields, as well as on the migration of events between the categories. An additional 10% uncertainty in the K factor used for the ${\mathrm{g}} {\mathrm{g}} \rightarrow {\mathrm{Z}} {\mathrm{Z}}$ prediction is applied as described in Sect. 7.1. A systematic uncertainty of 2% [32] in the branching fraction of ${\mathrm{H}} \rightarrow 4\ell$ only affects the signal yield.

Theoretical uncertainties that affect the predictions of the STXS production bins are described in Ref. [32]. From here on we will refer to these uncertainties as the theoretical uncertainty scheme (THU).

The THU for the ${\mathrm{g}} {\mathrm{g}} {\mathrm{H}}$ process includes 10 NPs, which account for uncertainties in the cross section prediction for exclusive jet bins (including the migration between the 0 and 1-jet, as well as between the 1 and $\ge$2-jet bins), the 2 jet and $\ge$3 jet ${\mathrm {VBF}}$ phase space, migrations around the $p_{{\mathrm {T}}} ^{{\mathrm{H}}}$ bin boundaries at 10, 60, and 120$\,\text {GeV}$, and the uncertainty in the $p_{{\mathrm {T}}} ^{{\mathrm{H}}}$ distribution due to missing higher order finite top quark mass corrections.

In the THU uncertainties for ${\mathrm {VBF}}$ and ${{\mathrm{V}}_{\mathrm{}}^{\mathrm{}}} {\mathrm{H}}$ production, additional sources are introduced to account for the uncertainty in the modeling of the $p_{{\mathrm {T}}} ^{{\mathrm{H}}}$, $m_{\text {jj}}$ and $p_{{\mathrm {T}}} ^{{{\mathrm{H}} \text {jj}}}$ distributions, as well as that of the number of jets in the event. A total of 6 NPs account for the migrations of events across the $m_{\text {jj}}$ boundaries at 60, 120, 350, 700, 1000, and 1500$\,\text {GeV}$. Two additional NPs account for migrations across the $p_{{\mathrm {T}}} ^{{\mathrm{H}}} = 200\,\text {GeV}$ and $p_{{\mathrm {T}}} ^{{\mathrm{H}} \text {jj}} = 25\,\text {GeV}$ bin boundaries. Finally, a single source is introduced to account for migrations between the zero and one jet, as well as the the two or more jet bins. In each case, the uncertainty is computed by varying the renormalization and factorization scales and recalculating the fractional breakdown of the ${\mathtt {qqH}}$ STXS stage 1.2 cross sections.

**Fig. 9:**
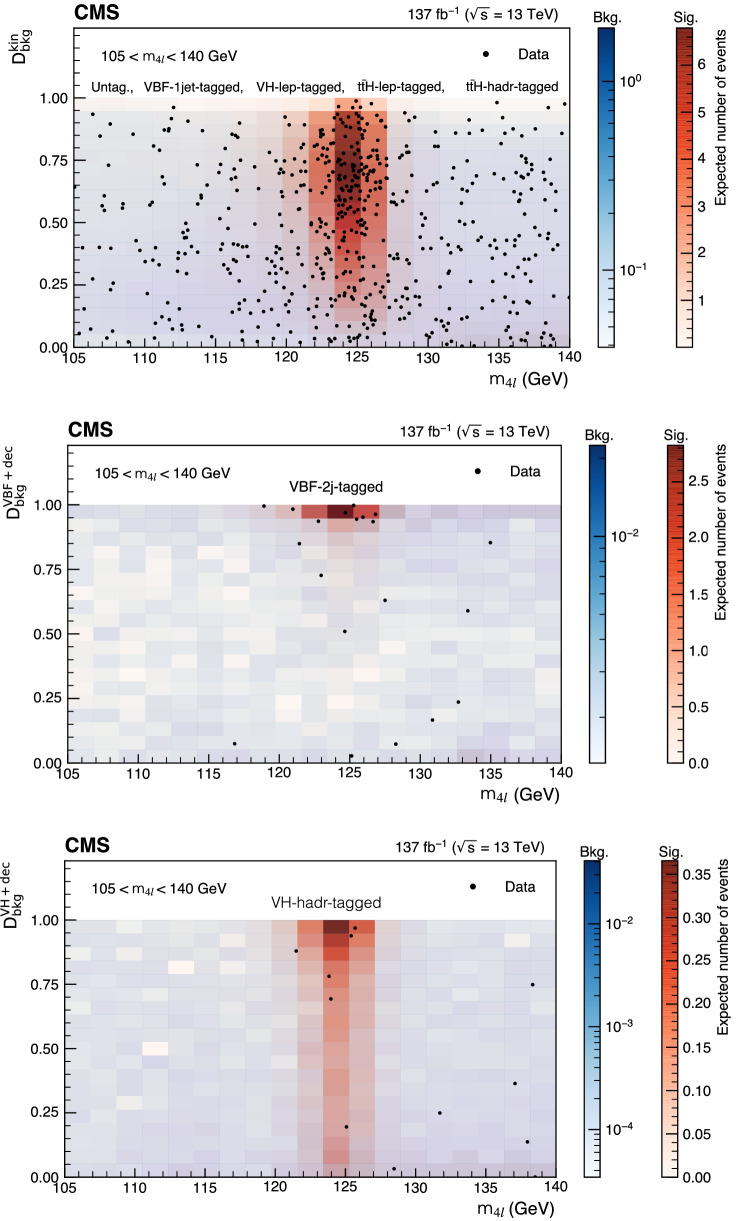
Distribution of three different kinematic discriminants versus $m_{4\ell }$: ${{\mathcal {D}}}^{\text {kin}}_{\text {bkg}}$ (upper), ${{\mathcal {D}}}^{{\mathrm {VBF}}+\text {dec}}_{\text {bkg}}$ (middle) and ${{\mathcal {D}}}^{{{\mathrm{V}}_{\mathrm{}}^{\mathrm{}}} {\mathrm{H}} +\text {dec}}_{\text {bkg}}$ (lower) shown in the mass region $105<m_{4\ell }<140\,\text {GeV}$. The blue scale represents the expected total number of ${\mathrm{Z}} {\mathrm{Z}}$, rare electroweak, and ${\mathrm{Z}}$+X background events. The red scale represents the number of expected SM H boson signal events for $m_{{\mathrm{H}}}=125\,\text {GeV}$. The points show the data from the categories listed in the legend

**Fig. 10:**
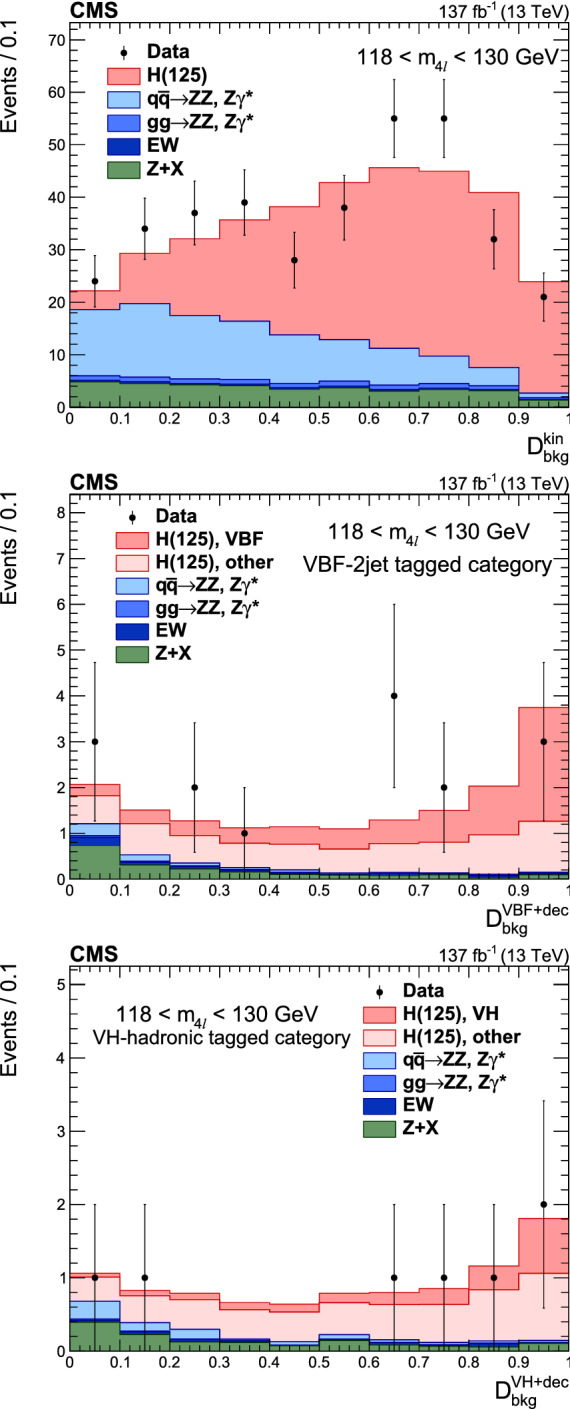
Distribution of kinematic discriminants in the mass region $118<m_{4\ell }<130\,\text {GeV}$: (uppper) ${{\mathcal {D}}}^{\text {kin}}_{\text {bkg}}$ , (center) ${{\mathcal {D}}}^{{\mathrm {VBF}}+\text {dec}}_{\text {bkg}}$, (lower) ${{\mathcal {D}}}^{{{\mathrm{V}}_{\mathrm{}}^{\mathrm{}}} {\mathrm{H}} +\text {dec}}_{\text {bkg}}$. Points with error bars represent the data and stacked histograms represent expected distributions of the signal and background processes. The yields of the different H boson production mechanisms with $m_{{\mathrm{H}}}=125\,\text {GeV}$, denoted as ${\mathrm{H}} (125)$, and those of the ${\mathrm{Z}} {\mathrm{Z}}$ and rare electroweak backgrounds are normalized to the SM expectations, while the ${\mathrm{Z}}$+X background yield is normalized to the estimate from the data. In the center and lower figures the SM H boson signal is separated into two components: the production mode which is targeted by the specific discriminant, and other production modes, where the gluon fusion process dominates

**Fig. 11:**
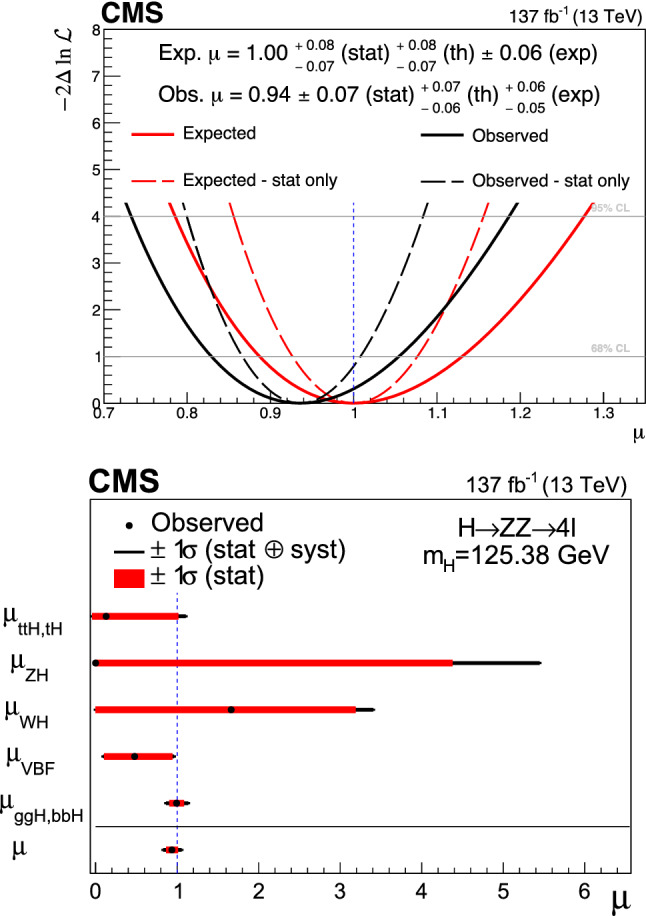
(Upper) The observed and expected profile likelihood scans of the inclusive signal strength modifier. The scans are shown both with (solid line) and without (dashed line) systematic uncertainties. (Lower) Results of likelihood scans for the signal strength modifiers corresponding to the five main SM H boson production mechanisms, compared to the SM prediction shown as a vertical dashed line. The thick black lines indicate the one standard deviation confidence intervals including both statistical and systematic sources. The thick red lines indicate the statistical uncertainties corresponding to the one standard deviation confidence intervals

A set of THU uncertainties is considered as NPs in the likelihood fit when signal strength modifiers, rather than STXS, are measured. In the STXS framework, THU uncertainties only enter at the interpretation step and are thus applied only to the SM cross section predictions.

Additional theoretical effects that only cause migration of signal and background events between categories originate from the modeling of the hadronization and the underlying event. The underlying event modeling uncertainty is determined by varying initial- and final-state radiation scales between 0.25 and 4 times their nominal value. The effects of the modeling of hadronization are determined by simulating additional events with the variation of the nominal pythia tune described in Sect. 3.

## Results

The reconstructed four-lepton invariant mass distribution is shown in Fig. 4 for the $4{\mathrm{e}}$, $4{{{\upmu }}_{\mathrm{}}^{\mathrm{}}}$ and $2{\mathrm{e}} 2{{{\upmu }}_{\mathrm{}}^{\mathrm{}}}$ events together, and is compared with the expectations for signal and background processes. The error bars on the data points correspond to the intervals at 68% confidence level ($\text {CL}$) [116]. The observed distribution agrees with the expectation within the statistical uncertainties over the whole spectrum.

The reconstructed four-lepton invariant mass distribution is shown in Fig. 5 for the three $4\ell$ final states and is compared with the expectations from signal and background processes.

The number of candidates observed in the data and the expected yields for $137{\,\text {fb}^{-1}}$, for the backgrounds and H boson signal after the full event selection, are given in Table 3 for each of the 22 reconstructed event categories (described in Sect. 6.2) for the $105<m_{4\ell }<140\,\text {GeV}$ mass window around the Higgs boson peak. Fig. 6 shows the number of expected and observed events for each of the categories.

**Table 4:** Best fit values and $\pm 1$ standard deviation uncertainties for the expected and observed signal strength modifiers at $m_{{\mathrm{H}}}=125.38\,\text {GeV}$. The statistical and systematic uncertainties are given separately

	Expected	Observed
$\mu _{{{{\mathrm{t}}_{\mathrm{}}^{\mathrm{}}} {}{{{\overline{{{\mathrm{t}}_{\mathrm{}}^{\mathrm{}}}}}}_{\mathrm{}}^{\mathrm{}}}} {\mathrm{H}},{{\mathrm{t}}_{\mathrm{}}^{\mathrm{}}} {\mathrm{H}}}$	$1.00^{+1.23}_{-0.77}\,\text {(stat)} ^ {+0.51}_{-0.06}\,\text {(syst)}$	$0.17 ^{+0.88}_{-0.17}\,\text {(stat)} ^{+0.42}_{-0.00}\,\text {(syst)}$
$\mu _{{\mathrm{W}} {\mathrm{H}}}$	$1.00^{+1.83}_{-1.00}\,\text {(stat)} ^{+0.75}_{-0.00}\,\text {(syst)}$	$1.66 ^{+1.52}_{-1.66}\,\text {(stat)} ^ {+0.85}_{-0.00}\,\text {(syst)}$
$\mu _{{\mathrm{Z}} {\mathrm{H}}}$	$1.00^{+4.79}_{-1.00}\,\text {(stat)} ^ {+6.76}_{-0.00}\,\text {(syst)}$	$0.00 ^{+4.38}_{-0.00}\,\text {(stat)} ^ {+3.24}_{-0.00}\,\text {(syst)}$
$\mu _{{\mathrm {VBF}}}$	$1.00^{+0.53}_{-0.44}\,\text {(stat)} ^ {+0.18}_{-0.12}\,\text {(syst)}$	$0.48 ^{+0.46}_{-0.37}\,\text {(stat)} ^ {+0.14}_{-0.10}\,\text {(syst)}$
$\mu _{{\mathrm{g}} {\mathrm{g}} {\mathrm{H}},{{\mathrm{b}}_{\mathrm{}}^{\mathrm{}}} {}{{{\overline{{{\mathrm{b}}_{\mathrm{}}^{\mathrm{}}}}}}_{\mathrm{}}^{\mathrm{}}} {\mathrm{H}}}$	$1.00 \pm 0.10\,\text {(stat)} ^ {+0.12}_{-0.10}\,\text {(syst)}$	$0.99 \pm 0.09\,\text {(stat)} ^ {+0.11}_{-0.09}\,\text {(syst)}$
$\mu$	$1.00^{+0.08}_{-0.07}\,\text {(stat)} ^{+0.10}_{-0.08}\,\text {(syst)}$	$0.94 \pm 0.07\,\text {(stat)} ^{+0.09}_{-0.08}\,\text {(syst)}$

**Fig. 12:**
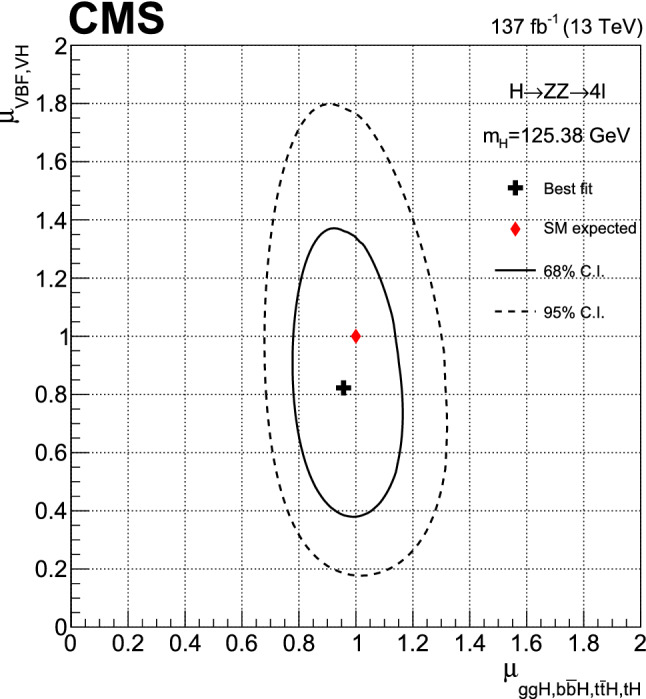
Result of the 2D likelihood scan for the $\mu _{{\mathrm {f}}}\equiv \mu _{{\mathrm{g}} {\mathrm{g}} {\mathrm{H}},\,{{{\mathrm{t}}_{\mathrm{}}^{\mathrm{}}} {}{{{\overline{{{\mathrm{t}}_{\mathrm{}}^{\mathrm{}}}}}}_{\mathrm{}}^{\mathrm{}}}} {\mathrm{H}},{{\mathrm{b}}_{\mathrm{}}^{\mathrm{}}} {}{{{\overline{{{\mathrm{b}}_{\mathrm{}}^{\mathrm{}}}}}}_{\mathrm{}}^{\mathrm{}}} {\mathrm{H}},{{\mathrm{t}}_{\mathrm{}}^{\mathrm{}}} {\mathrm{H}}}$ and $\mu _{{{\mathrm{V}}_{\mathrm{}}^{\mathrm{}}}}\equiv \mu _{{\mathrm {VBF}},{{\mathrm{V}}_{\mathrm{}}^{\mathrm{}}} {\mathrm{H}}}$ signal strength modifiers. The solid and dashed contours show the 68 and 95% $\text {CL}$ regions, respectively. The cross indicates the best fit value, and the diamond represents the expected value for the SM Higgs boson

The reconstructed invariant masses of the ${\mathrm{Z}} _1$ and ${\mathrm{Z}} _2$ dilepton systems are shown in Fig. 7 for $118<m_{4\ell }<130\,\text {GeV}$, together with their 2D distribution in the $105<m_{4\ell }<140\,\text {GeV}$ mass region. The distribution of the discriminants used for event categorization along with the corresponding working point values are shown in Fig. 8.

The results presented in Sects. 10.1 and 10.2 are extracted with a two-dimensional likelihood fit that relies on two variables, the four-lepton invariant mass $m_{4\ell }$ and the matrix element kinematic discriminant ${\mathcal {D}}$. The fiducial cross section measurements are extracted with a one-dimensional likelihood fit that relies only on the four-lepton invariant mass. The fit procedure and results are presented in Sect. 10.3. The fit is performed in the $105<m_{4\ell }<140\,\text {GeV}$ mass region. The parameters of interest (POIs) are estimated with their corresponding confidence intervals using a profile likelihood ratio test statistic [117, 118], in which the experimental and theoretical uncertainties are incorporated via NPs. The choice of the POIs depends on the specific measurement under consideration, while the remaining parameters are treated as NPs. All the POIs considered in the analysis are forced to be greater than or equal to zero; this reflects the fact that the signal yield is substantially larger than the background yield in the mass range studied. Negative POIs would imply negative signal strength modifiers and a negative probability density function (pdf). We define a two-dimensional pdf as the product of two one-dimensional pdfs:

6 $$\begin{aligned} f(m_{4\ell },{\mathcal {D}}) = {\mathcal {P}}(m_{4\ell }) {\mathcal {P}}({\mathcal {D}}|m_{4\ell }). \end{aligned}$$

The first term, ${\mathcal {P}}(m_{4\ell })$, is the unbinned analytical shape described in Sect. 8 for signals and Sect. 7 for backgrounds. The second term, ${\mathcal {P}}({\mathcal {D}}|m_{4\ell })$, is a binned template of ${\mathcal {D}}$ that is conditional to $m_{4\ell }$. This is achieved by creating a two-dimensional template of $m_{4\ell }$ vs. ${\mathcal {D}}$ and normalizing it to 1 for each bin of $m_{4\ell }$.

**Fig. 13:**
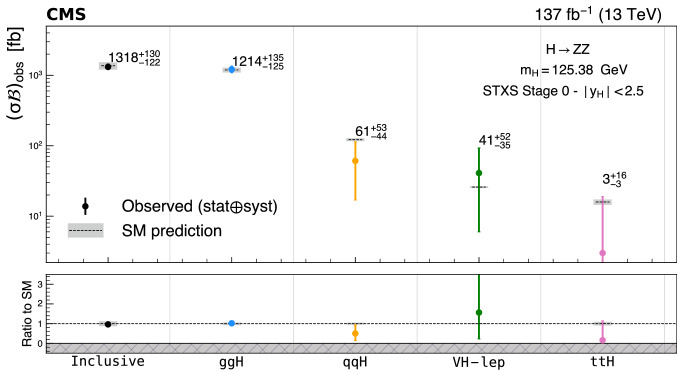
The measured product of cross section times branching fraction for ${\mathrm{H}} \rightarrow {\mathrm{Z}} {\mathrm{Z}}$ decay $(\sigma {\mathcal {B}})_{{\text {obs}}}$ and the SM predictions $(\sigma {\mathcal {B}})_{{\mathrm {SM}}}$ for the stage 0 STXS production bins and the inclusive measurement at $m_{{\mathrm{H}}}=125.38\,\text {GeV}$. Points with error bars represent measured values and black dashed lines with gray uncertainty bands represent the SM predictions. In the bottom panel ratios of the measured cross sections and the SM predictions are shown along with the uncertainties for each of the bins and the inclusive measurement

**Fig. 14:**
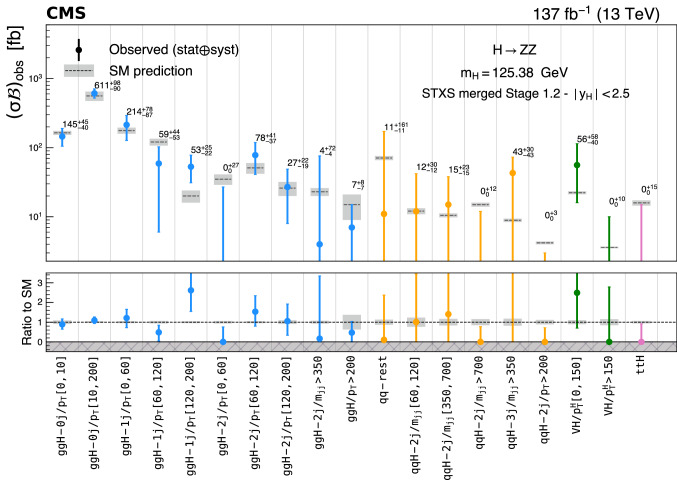
The measured cross sections $(\sigma {\mathcal {B}})_{{\text {obs}}}$ and the SM predictions $(\sigma {\mathcal {B}})_{{\mathrm {SM}}}$ for ${\mathrm{H}} \rightarrow {\mathrm{Z}} {\mathrm{Z}}$ decay and the merged stage 1.2 STXS production bins at $m_{{\mathrm{H}}}=125.38\,\text {GeV}$. Points with error bars represent measured values and black dashed lines with gray uncertainty bands represent the SM predictions. In the bottom panel ratios of the measured cross sections and the SM predictions are shown with corresponding uncertainties for each of the bins

In almost all sub-categories we use a decay-only kinematic discriminant (${\mathcal {D}}={{\mathcal {D}}}^{\text {kin}}_{\text {bkg}}$) to separate the H boson signal from the background as defined in Eq. (3). Conversely, in the sub-categories of the VBF-2jet-tagged, the ${\mathcal {D}}={{\mathcal {D}}}^{{\mathrm {VBF}}+\text {dec}}_{\text {bkg}}$ discriminant (defined in Eq. (4)) is used, which is sensitive to the VBF production mechanism. Similarly, in two sub-categories of the VH-hadronic-tagged category, the ${\mathcal {D}}={{\mathcal {D}}}^{{{\mathrm{V}}_{\mathrm{}}^{\mathrm{}}} {\mathrm{H}} +\text {dec}}_{\text {bkg}}$ discriminant (defined in Eq. (5)) is used.

The ${\mathrm{g}} {\mathrm{g}} {\mathrm{H}}$, ${\mathrm {VBF}}$, ${\mathrm{W}} {\mathrm{H}}$, ${\mathrm{Z}} {\mathrm{H}}$ and ${{{\mathrm{t}}_{\mathrm{}}^{\mathrm{}}} {}{{{\overline{{{\mathrm{t}}_{\mathrm{}}^{\mathrm{}}}}}}_{\mathrm{}}^{\mathrm{}}}} {\mathrm{H}}$ samples are used to build different signal templates for each of the nineteen STXS production bins described in Sect. 6.1. Irreducible background templates are built starting from ${{\mathrm{q}}_{\mathrm{}}^{\mathrm{}}} {{{\overline{{{\mathrm{q}}_{\mathrm{}}^{\mathrm{}}}}}}_{\mathrm{}}^{\mathrm{}}} \rightarrow {\mathrm{Z}} {\mathrm{Z}}$ and ${\mathrm{g}} {\mathrm{g}} \rightarrow {\mathrm{Z}} {\mathrm{Z}}$ samples. Finally, reducible background templates are built using data driven methods described in Sect. 7.2. Following the described procedure, ${\mathcal {P}}({\mathcal {D}}|m_{4\ell })$ templates are obtained for the twenty-two event categories and the three final states ($4\mu$, $4{\mathrm{e}}$, $2{\mathrm{e}} 2\mu$).

The unbinned likelihood function, ${\mathcal {L}}(\vec {\mu })$, is defined as the product over *N* observed events:

7 $$\begin{aligned} {\mathcal {L}}(\vec {\mu }) =&\frac{1}{N} \prod _{\text {events}} \Bigg ( \sum _{i=1}^{19} \mu _i S_i^{jk} f_{S}^{ijk}(m_{4\ell },{\mathcal {D}}) \nonumber \\&+B^{jk} f_{B}^{jk}(m_{4\ell },{\mathcal {D}})\Bigg )\hbox {e}^{-\sum _{i} \mu _i S_i^{jk} + B^{jk}}, \end{aligned}$$

where $\mu _i$ is the signal strength modifier for the production bin *i*, $S_i^{jk}$ are the predicted SM rates of events in the production bin *i* that are observed in the reconstructed event category *j* and final state *k*, $B^{jk}$ are the predicted background rates in the reconstructed event category *j* and final state *k*, $f_{S}^{ijk} (m_{4\ell },{\mathcal {D}})$ are the pdfs for the signal, and $f_{B}^{jk}(m_{4\ell },{\mathcal {D}})$ the pdfs for the background.

The correlation of the kinematic discriminants ${{\mathcal {D}}}^{\text {kin}}_{\text {bkg}}$, ${{\mathcal {D}}}^{{\mathrm {VBF}}+\text {dec}}_{\text {bkg}}$, and ${{\mathcal {D}}}^{{{\mathrm{V}}_{\mathrm{}}^{\mathrm{}}} {\mathrm{H}} +\text {dec}}_{\text {bkg}}$ with the four-lepton invariant mass is shown in Fig. 9 for the mass interval $105<m_{4\ell }<140\,\text {GeV}$. Their distributions for the mass interval $118<m_{4\ell }<130\,\text {GeV}$ are shown in Fig. 10.

### Signal strength modifier

A simultaneous fit to all categories is performed to extract the signal strength modifier, defined as the ratio of the observed H boson yield in the ${\mathrm{H}} \rightarrow 4\ell$ decay channel to the standard model expectation.

The combined measurement of the inclusive signal strength modifier is measured to be $\mu = 0.94^{+0.12}_{-0.11}$ or $\mu = 0.94 \pm 0.07\,\text {(stat)} ^{+0.07}_{-0.06}\,\text {(theo)} ^{+0.06}_{-0.05}\,(\text {exp})$ at a fixed mass value $m_{{\mathrm{H}}}= 125.38\,\text {GeV}$, which is the current most precise measurement of the H boson mass published by the CMS Collaboration [119]. In all subsequent fits, $m_{{\mathrm{H}}}$ is fixed to this value. The dominant experimental sources of systematic uncertainty are the uncertainties in the lepton identification efficiencies and luminosity measurement, while the dominant theoretical source is the uncertainty in the total gluon fusion cross section. The contributions to the total uncertainty from experimental and theoretical sources are found to be similar in magnitude. The signal strength modifiers are further studied in terms of the five main SM Higgs boson production mechanisms, namely ${\mathrm{g}} {\mathrm{g}} {\mathrm{H}}$, ${\mathrm {VBF}}$, ${\mathrm{Z}} {\mathrm{H}}$, ${\mathrm{W}} {\mathrm{H}}$, and ${{{\mathrm{t}}_{\mathrm{}}^{\mathrm{}}} {}{{{\overline{{{\mathrm{t}}_{\mathrm{}}^{\mathrm{}}}}}}_{\mathrm{}}^{\mathrm{}}}} {\mathrm{H}}$. The contributions of the ${{\mathrm{b}}_{\mathrm{}}^{\mathrm{}}} {}{{{\overline{{{\mathrm{b}}_{\mathrm{}}^{\mathrm{}}}}}}_{\mathrm{}}^{\mathrm{}}} {\mathrm{H}}$ and ${{\mathrm{t}}_{\mathrm{}}^{\mathrm{}}} {\mathrm{H}}$ production modes are also taken into account. The relative normalizations of the ${{\mathrm{b}}_{\mathrm{}}^{\mathrm{}}} {}{{{\overline{{{\mathrm{b}}_{\mathrm{}}^{\mathrm{}}}}}}_{\mathrm{}}^{\mathrm{}}} {\mathrm{H}}$ and the gluon fusion contributions are kept fixed in the fit, and so are the ${{\mathrm{t}}_{\mathrm{}}^{\mathrm{}}} {\mathrm{H}}$ and ${{{\mathrm{t}}_{\mathrm{}}^{\mathrm{}}} {}{{{\overline{{{\mathrm{t}}_{\mathrm{}}^{\mathrm{}}}}}}_{\mathrm{}}^{\mathrm{}}}} {\mathrm{H}}$ ones. The results are shown in Fig. 11 for the observed and expected profile likelihood scans of the inclusive signal strength modifier and those for the signal strength modifiers of the five main SM Higgs boson production mechanisms. The corresponding numerical values, including the decomposition of the uncertainties into statistical and systematic components, as well as the expected uncertainties, are given in Table 4.

**Table 5:** Best fit values and $\pm 1$ standard deviation uncertainties for the measured cross sections $(\sigma {\mathcal {B}})_{{\text {obs}}}$, the SM predictions $(\sigma {\mathcal {B}})_{{\mathrm {SM}}}$, and their ratio for the stage 0 STXS production bins at $m_{{\mathrm{H}}}=125.38 \,\text {GeV}$ for ${\mathrm{H}} \rightarrow {\mathrm{Z}} {\mathrm{Z}}$ decay

	$(\sigma {\mathcal {B}})_{{\text {obs}}}$ (fb)	$(\sigma {\mathcal {B}})_{{\mathrm {SM}}}$ (fb)	$(\sigma {\mathcal {B}})_{{\text {obs}}}/ (\sigma {\mathcal {B}})_{{\mathrm {SM}}}$
${\mathtt {ttH}}$	$3^{+16}_{-3}$	$15.9 \pm 1.4$	$0.16^{+0.98}_{-0.16}$
${\mathtt {VH}}\text {-}{\mathtt {lep}}$	$41^{+52}_{-35}$	$25.9 \pm 0.8$	$1.56^{+1.99}_{-1.34}$
${\mathtt {qqH}}$	$61^{+53}_{-44}$	$122 \pm 6$	$0.50^{+0.44}_{-0.36}$
${\mathtt {ggH}}$	$1214^{+135}_{-125}$	$1192 \pm 95$	$1.02^{+0.11}_{-0.10}$
${\mathrm {Inclusive}}$	$1318^{+130}_{-122}$	$1369\pm 164$	$0.96^{+0.10}_{-0.09}$

**Table 6:** Best fit values and $\pm 1$ standard deviation uncertainties for the measured cross sections $(\sigma {\mathcal {B}})_{{\text {obs}}}$, the SM predictions $(\sigma {\mathcal {B}})_{{\mathrm {SM}}}$, and their ratio for the merged stage 1.2 STXS production bins at $m_{{\mathrm{H}}}=125.38\,\text {GeV}$ for ${\mathrm{H}} \rightarrow {\mathrm{Z}} {\mathrm{Z}}$ decay

	$(\sigma {\mathcal {B}})_{{\text {obs}}}$ (fb)	$(\sigma {\mathcal {B}})_{{\mathrm {SM}}}$ (fb)	$(\sigma {\mathcal {B}})_{{\text {obs}}}/ (\sigma {\mathcal {B}})_{{\mathrm {SM}}}$
${\mathtt {ggH}}\text {-}{\mathtt {0j/p}}_{\mathtt {T}}{\mathtt{[0,10]}}$	$145^{+45}_{-40}$	$164 \pm 11$	$0.89^{+0.28}_{-0.24}$
${\mathtt {ggH}}\text {-}{\mathtt {0j/p}}_{\mathtt {T}}{\mathtt{[10,200]}}$	$611^{+98}_{-90}$	$561 \pm 87$	$1.09^{+0.17}_{-0.16}$
${\mathtt {ggH}}\text {-}{\mathtt {1j/p}}_{\mathtt {T}}{\mathtt{[0,60]}}$	$214^{+78}_{-87}$	$177 \pm 18$	$1.21^{+0.44}_{-0.49}$
${\mathtt {ggH}}\text {-}{\mathtt {1j/p}}_{\mathtt {T}}{\mathtt{[60,120]}}$	$59^{+44}_{-53}$	$121 \pm 14$	$0.48^{+0.37}_{-0.44}$
${\mathtt {ggH}}\text {-}{\mathtt {1j/p}}_{\mathtt {T}}{\mathtt{[120,200]}}$	$53^{+25}_{-22}$	$20 \pm 4$	$2.62^{+1.24}_{-1.08}$
${\mathtt {ggH}}\text {-}{\mathtt {2j/p}}_{\mathtt {T}}{\mathtt{[0,60]}}$	$0^{+27}_{-0}$	$35 \pm 6$	$0.00^{+0.76}_{-0.00}$
${\mathtt {ggH}}\text {-}{\mathtt {2j/p}}_{\mathtt {T}}{\mathtt{[60,120]}}$	$78^{+41}_{-37}$	$51 \pm 9$	$1.53^{+0.81}_{-0.73}$
${\mathtt {ggH}}\text {-}{\mathtt {2j/p}}_{\mathtt {T}}{\mathtt{[120,200]}}$	$27^{+22}_{-19}$	$26 \pm 6$	$1.06^{+0.87}_{-0.72}$
${\mathtt {ggH}}\text {-}{\mathtt {2j/m}}_{\mathtt {{jj}}}{\mathtt{>350}}$	$4^{+72}_{-4}$	$23 \pm 3$	$0.17^{+3.2}_{-0.17}$
${\mathtt {ggH/p}}_{\mathtt {T}}{\mathtt{>200}}$	$7^{+8}_{-7}$	$15 \pm 6$	$0.47^{+0.56}_{-0.47}$
${\mathtt {qqH}}\text {-}{\mathtt {rest}}$	$11^{+161}_{-11}$	$71 \pm 5$	$0.15^{+2.27}_{-0.15}$
${\mathtt {qqH}}\text {-}{\mathtt {2j/m}}_{\mathtt {{jj}}}{\mathtt{[60,120]}}$	$12^{+30}_{-12}$	$12.1 \pm 1.2$	$1.01^{+2.45}_{-1.01}$
${\mathtt {qqH}}\text {-}{\mathtt {2j/m}}_{\mathtt {{jj}}}{\mathtt{[350,700]}}$	$15^{+23}_{-15}$	$10.5 \pm 0.7$	$1.41^{+2.21}_{-1.41}$
${\mathtt {qqH}}\text {-}{\mathtt {2j/m}}_{\mathtt {{jj}}}{\mathtt{>700}}$	$0^{+12}_{-0}$	$15 \pm 1$	$0.00^{+0.77}_{-0.00}$
${\mathtt {qqH}}\text {-}{\mathtt {3j/m}}_{\mathtt {{jj}}}{\mathtt{>350}}$	$43^{+30}_{-43}$	$8.9 \pm 0.5$	$4.84^{+3.38}_{-4.84}$
${\mathtt {qqH}}\text {-}{\mathtt {2j/p}}_{\mathtt {T}}{\mathtt{>200}}$	$0^{+3}_{-0}$	$4.2 \pm 0.2$	$0.00^{+0.72}_{-0.00}$
${\mathtt {VH}}\text {-}{\mathtt {lep/p}}_{\mathtt {T^{\mathrm{H}}}}{\mathtt{[0,150]}}$	$56^{+58}_{-40}$	$22.3 \pm 1.1$	$2.49^{+2.60}_{-1.79}$
${\mathtt {VH}}\text {-}{\mathtt {lep/p}}_{\mathtt {T^{\mathrm{H}}}}{\mathtt{>150}}$	$0^{+10}_{-0}$	$3.6 \pm 0.1$	$0.00^{+2.79}_{-0.00}$
${\mathtt {ttH}}$	$0^{+15}_{-0}$	$15.9 \pm 1.4$	$0.00^{+0.91}_{-0.00}$

The dependence of the measured signal strengths on the profiling of $m_{{\mathrm{H}}}$ is checked and found to have a small impact both on the inclusive results and those in terms of the five main H boson production mechanisms, well within the measurement uncertainties. The best fit signal value changes at most by 4% and the profiled value of the mass is found to be $m_{{\mathrm{H}}}=125.09^{+0.15}_{-0.14}\,\text {(stat)} \,\text {GeV}$. It is important to note here that the precise determination of $m_{{\mathrm{H}}}$ and the systematic uncertainties that enter its measurement are beyond the scope of this analysis.

Two signal strength modifiers, $\mu _{{\mathrm {f}}}\equiv \mu _{{\mathrm{g}} {\mathrm{g}} {\mathrm{H}},\,{{{\mathrm{t}}_{\mathrm{}}^{\mathrm{}}} {}{{{\overline{{{\mathrm{t}}_{\mathrm{}}^{\mathrm{}}}}}}_{\mathrm{}}^{\mathrm{}}}} {\mathrm{H}},{{\mathrm{b}}_{\mathrm{}}^{\mathrm{}}} {}{{{\overline{{{\mathrm{b}}_{\mathrm{}}^{\mathrm{}}}}}}_{\mathrm{}}^{\mathrm{}}} {\mathrm{H}},{{\mathrm{t}}_{\mathrm{}}^{\mathrm{}}} {\mathrm{H}}}$ and $\mu _{{{\mathrm{V}}_{\mathrm{}}^{\mathrm{}}}}\equiv \mu _{{\mathrm {VBF}},{{\mathrm{V}}_{\mathrm{}}^{\mathrm{}}} {\mathrm{H}}}$, are introduced for the fermion and vector-boson induced contributions to the expected SM cross section. A two-parameter fit is performed simultaneously to the events reconstructed in all categories, leading to $\mu _{{\mathrm {f}}}=0.96^{+0.14}_{-0.12}$ and $\mu _{{{\mathrm{V}}_{\mathrm{}}^{\mathrm{}}}}=0.82^{+0.36}_{-0.31}$. The expected values for $m_{{\mathrm{H}}}=125.38\,\text {GeV}$ are $\mu _{{\mathrm {f}}}=1.00^{+0.15}_{-0.13}$ and $\mu _{{{\mathrm{V}}_{\mathrm{}}^{\mathrm{}}}}=1.00^{+0.39}_{-0.33}$. The 68 and 95% $\text {CL}$ contours in the ($\mu _{{\mathrm {f}}},\mu _{{{\mathrm{V}}_{\mathrm{}}^{\mathrm{}}}}$) plane are shown in Fig. 12 and the SM predictions lie within the 68% $\text {CL}$ regions of this measurement.

### Simplified template cross section

The results for the H boson product of cross section times branching fraction for ${\mathrm{H}} \rightarrow {\mathrm{Z}} {\mathrm{Z}}$ decay, $(\sigma {\mathcal {B}})_{{\text {obs}}}$, and comparisons with the SM expectation, $(\sigma {\mathcal {B}})_{{\mathrm {SM}}}$, for the stages of production bins defined in Sect. 6.1, are shown in Fig. 13 for the stage 0 and in Fig. 14 for the merged stage 1.2. The corresponding numerical values are given in Tables 5 and 6 .

**Fig. 15:**
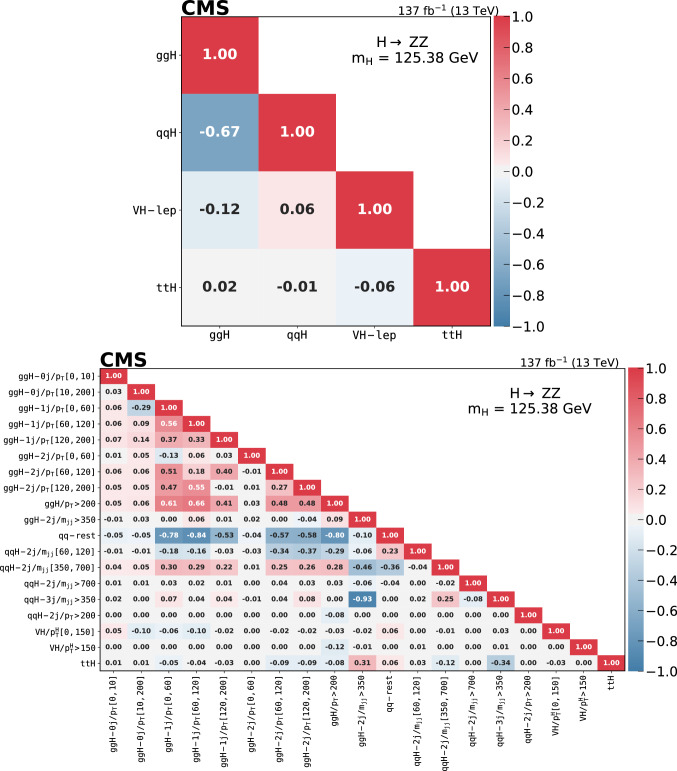
Correlation matrices between the measured cross sections for the stage 0 (upper) and the merged stage 1.2 (lower) for ${\mathrm{H}} \rightarrow {\mathrm{Z}} {\mathrm{Z}}$ decay

As discussed, the set of THU uncertainties described in Sect. 9.2 is not considered for the STXS measurements: THU uncertainties are model dependent and should be only considered in the interpretation of the results. Therefore, the THU uncertainties are included in the SM predictions of the cross section. The correlation matrices are shown in Fig. 15. The dominant experimental sources of systematic uncertainty are the same as for the signal strength modifiers measurement, while the dominant theoretical source is the uncertainty in the category migration for the ggH process.

**Table 7:** Summary of requirements used in the definition of the fiducial phase space for the ${\mathrm{H}} \rightarrow 4\ell$ cross section measurements

Requirements for the ${\mathrm{H}} \rightarrow 4\ell$ fiducial phase space	
*Lepton kinematics and isolation*	
Leading lepton $p_{{\mathrm {T}}}$	$p_{{\mathrm {T}}} > 20\,\text {GeV}$
Next-to-leading lepton $p_{{\mathrm {T}}}$	$p_{{\mathrm {T}}} > 10\,\text {GeV}$
Additional electrons (muons) $p_{{\mathrm {T}}}$	$p_{{\mathrm {T}}} > 7 (5)\,\text {GeV}$
Pseudorapidity of electrons (muons)	$|\eta |<$ 2.5 (2.4)
Sum of scalar $p_{{\mathrm {T}}}$ of all stable particles within $\varDelta R < 0.3$ from lepton	$<0.35 p_{{\mathrm {T}}}$
*Event topology*	
Existence of at least two same-flavor OS lepton pairs, where leptons satisfy criteria above	
Inv. mass of the ${\mathrm{Z}} _1$ candidate	$40< m_{{\mathrm{Z}} _{1}} < 120 \,\text {GeV}$
Inv. mass of the ${\mathrm{Z}} _2$ candidate	$12< m_{{\mathrm{Z}} _{2}} < 120 \,\text {GeV}$
Distance between selected four leptons	$\varDelta R(\ell _{i},\ell _{j})>0.02$ for any $i\ne j$
Inv. mass of any opposite sign lepton pair	$m_{\ell ^{+}\ell '^{-}}>4 \,\text {GeV}$
Inv. mass of the selected four leptons	$105< m_{4\ell }< 140 \,\text {GeV}$

**Table 8:** Summary of the fraction of signal events for different SM signal production modes within the fiducial phase space (acceptance ${\mathcal {A}}_{{\mathrm {fid}}}$), reconstruction efficiency ($\epsilon$) for signal events in the fiducial phase space, and ratio of the number of reconstructed events outside the fiducial phase space to that of the reconstructed events in the fiducial phase space ($f_{{\mathrm {nonfid}}}$). For all production modes the values given are for $m_{{\mathrm{H}}}= 125\,\text {GeV}$. Also shown in the last column is the factor $(1+f_{{\mathrm {nonfid}}})\epsilon$ which regulates the signal yield for a given fiducial cross section, as shown in Eq. (8). The uncertainties listed are statistical only. The theoretical uncertainty in ${\mathcal {A}}_{{\mathrm {fid}}}$ for the SM is less than 1%

Signal process	${\mathcal {A}}_{{\mathrm {fid}}}$	$\epsilon$	$f_{{\mathrm {nonfid}}}$	$(1+f_{{\mathrm {nonfid}}})\epsilon$
${\mathrm{g}} {\mathrm{g}} {\mathrm{H}}$ (powheg)	0.402 ± 0.001	0.598 ± 0.002	0.054 ± 0.001	0.631 ± 0.002
${\mathrm {VBF}}$	0.445 ± 0.002	0.615 ± 0.002	0.043 ± 0.001	0.641 ± 0.003
${\mathrm{W}} {\mathrm{H}}$	0.329 ± 0.002	0.604 ± 0.003	0.078 ± 0.002	0.651 ± 0.004
${\mathrm{Z}} {\mathrm{H}}$	0.340 ± 0.003	0.613 ± 0.005	0.082 ± 0.004	0.663 ± 0.006
${{{\mathrm{t}}_{\mathrm{}}^{\mathrm{}}} {}{{{\overline{{{\mathrm{t}}_{\mathrm{}}^{\mathrm{}}}}}}_{\mathrm{}}^{\mathrm{}}}} {\mathrm{H}}$	0.315 ± 0.004	0.588 ± 0.007	0.181 ± 0.009	0.694 ± 0.010

### Fiducial cross section

In this section the cross section measurement for the process ${\mathrm{p}} {\mathrm{p}} \rightarrow {\mathrm{H}} \rightarrow 4\ell$ within a fiducial volume that closely matches the reconstruction level selection is presented. In particular, the integrated fiducial cross section is measured as well as differential cross sections as a function of the transverse momentum of the H boson ($p_{{\mathrm {T}}} ^{{\mathrm{H}}}$), its rapidity ($|y^{{\mathrm{H}}} |$), the number of associated jets ($N^{\text {j}}$), and the transverse momentum of the leading jet ($p_{{\mathrm {T}}} ^{\text {j}}$). These measurements are largely independent of the assumptions on the relative fractions and kinematic distributions of the individual production modes. The definition of the fiducial volume is based on generator-level quantities and is identical to that in Ref. [18]. In order to reduce the experimental uncertainties, only jets with $p_{{\mathrm {T}}} ^{\text {j}}>30\,\text {GeV}$ and $|\eta ^{\text {j}} |<2.5$ are considered for the differential cross sections as a function of jet observables. An increase in model dependence compared to Ref. [25] is observed when using the ${\mathrm{Z}} {\mathrm{Z}}$ candidate selection at reconstruction level where the candidate with the best ${{\mathcal {D}}}^{\text {kin}}_{\text {bkg}}$ discriminant value is chosen. Therefore, the fiducial cross section measurement is performed using the event selection algorithm in Ref. [25]. Specifically, the ${\mathrm{Z}} _{1}$ candidate is chosen to be the one with $m({\mathrm{Z}} _1)$ closest to the nominal ${\mathrm{Z}}$ boson mass, and in cases where multiple ${\mathrm{Z}} _{2}$ candidates satisfy all criteria, the pair of leptons with the largest sum of the transverse momenta magnitudes is chosen. The full fiducial volume definition is detailed in Table 7 and the acceptance for various SM production modes is given in Table 8.

**Fig. 16:**
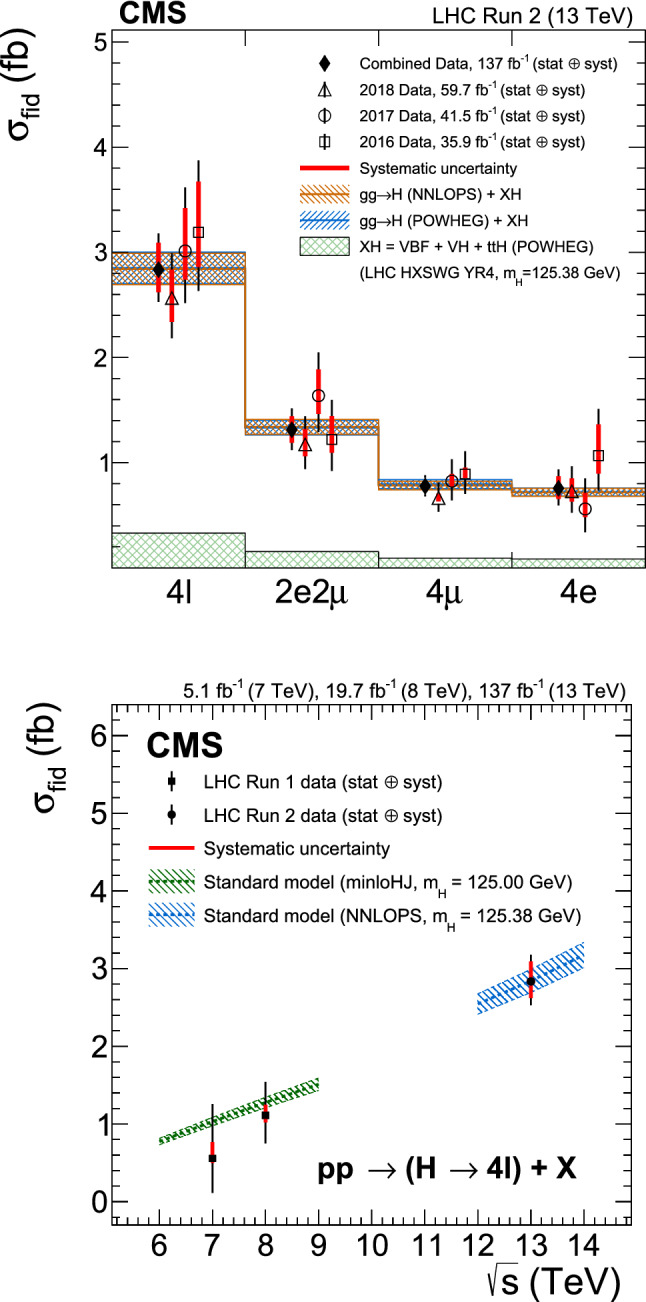
The measured inclusive fiducial cross section in different final states (upper) and integrated as a function of $\sqrt{s}$ (lower). The acceptance is calculated using powheg at $\sqrt{s}=13\,\text {TeV}$ and HRes [107, 109] at $\sqrt{s}=$ 7 and 8$\,\text {TeV}$, and the total gluon fusion cross section and uncertainty are taken from Ref. [58]. The fiducial volume for $\sqrt{s}=6$–$9\,\text {TeV}$ uses the lepton isolation definition from Ref. [25] and the SM predictions and measurements are calculated at $m_{{\mathrm{H}}}=125.0\,\text {GeV}$, while for $\sqrt{s}=12$–$14\,\text {TeV}$ the definition described in the text is used and SM predictions and measurements are calculated at $m_{{\mathrm{H}}}= 125.38\,\text {GeV}$

A maximum likelihood fit of the signal and background parameterizations to the observed $4\ell$ mass distribution, $N_{{\mathrm {obs}}}(m_{4\ell })$, is performed to extract the integrated fiducial cross section for the process ${\mathrm{p}} {\mathrm{p}} \rightarrow {\mathrm{H}} \rightarrow 4\ell$ ($\sigma _{{\mathrm {fid}}}$). The fit is carried out inclusively (i.e., without any event categorization) and does not use the ${{\mathcal {D}}}^{\text {kin}}_{\text {bkg}}$ observable in order to minimize the model dependence. The fit is performed simultaneously in all final states and assumes a H boson mass $m_{{\mathrm{H}}}=125.38\,\text {GeV}$, while the branching fractions of the H boson to different final states ($4{\mathrm{e}},4{{{\upmu }}_{\mathrm{}}^{\mathrm{}}},2{\mathrm{e}} 2{{{\upmu }}_{\mathrm{}}^{\mathrm{}}}$) are free parameters in the fit. The systematic uncertainties described in Sect. 9 are included in the form of NPs and the results are obtained using an asymptotic approach [118] with a test statistic based on the profile likelihood ratio [117]. This procedure accounts for the unfolding of detector effects from the observed distributions and is the same as in Refs. [25, 120].

The number of expected events in each final state *f* and in each bin *i* of a given observable is expressed as a function of $m_{4\ell }$ as:

8 $$\begin{aligned} \begin{aligned} N_{{\mathrm {exp}}}^{f,i}(m_{4\ell })&= N_{{\mathrm {fid}}}^{f,i}(m_{4\ell })+N_{{\mathrm {nonfid}}}^{f,i}(m_{4\ell })+N_{{\mathrm {nonres}}}^{f,i}(m_{4\ell })\\&\quad +N_{\text {bkg}}^{f,i}(m_{4\ell }) \\&=\sum _j\epsilon _{i,j}^{f} \left( 1+f_{{\mathrm {nonfid}}}^{f,i} \right) \sigma _{{\mathrm {fid}}}^{f,j} {\mathcal {L}}{\mathcal {P}}_{{\mathrm {res}}}(m_{4\ell }) \\&\quad + N_{{\mathrm {nonres}}}^{f,i}{\mathcal {P}}_{{\mathrm {nonres}}}(m_{4\ell })+N_{\text {bkg}}^{f,i}{\mathcal {P}}_{\text {bkg}}(m_{4\ell }). \end{aligned} \end{aligned}$$

The shape of the resonant signal contribution, ${\mathcal {P}}_{{\mathrm {res}}}(m_{4\ell })$, is described by a double-sided Crystal Ball function as discussed in Sect. 8, and the normalization is used to extract the fiducial cross section. The non-resonant signal function, ${\mathcal {P}}_{{\mathrm {nonres}}}(m_{4\ell })$, is determined by the ${\mathrm{W}} {\mathrm{H}}$, ${\mathrm{Z}} {\mathrm{H}}$, and ${{{\mathrm{t}}_{\mathrm{}}^{\mathrm{}}} {}{{{\overline{{{\mathrm{t}}_{\mathrm{}}^{\mathrm{}}}}}}_{\mathrm{}}^{\mathrm{}}}} {\mathrm{H}}$ contributions where one of the leptons from the H boson decay is lost or not selected. It is modeled by a Landau distribution with shape parameters constrained in the fit to be within a range determined from simulation. This contribution is referred to as the “combinatorial signal” and is treated as a background in this measurement.

The quantity $\epsilon _{i,j}^{f}$ represents the detector response matrix that maps the number of expected events in bin *j* of a given observable at the fiducial level to the number of expected events in bin *i* at the reconstruction level. This response matrix is determined using simulated signal samples and includes corrections for residual differences between data and simulation. In the case of the integrated fiducial cross section measurement, the response matrices become single numbers, which are listed in Table 8 for different SM production mechanism.

An additional resonant contribution arises from events which are accepted but do not originate from the fiducial phase space. These events are due to detector effects that cause differences between the quantities used for the fiducial phase space definition and the corresponding quantities at the reconstruction level. This contribution is treated as background and is referred to as the “non-fiducial signal” contribution. A simulated sample is used to verify that the shape of the distribution for these events is identical to that of the fiducial signal, and its normalization is fixed to the corresponding fraction of the fiducial signal. The value of this fraction, which we denote as $f_{{\mathrm {nonfid}}}$, is determined from simulation for each of the signal models studied. The value of $f_{{\mathrm {nonfid}}}$ for different signal models is shown in Table 8.

The integrated fiducial cross section is measured to be

$$\begin{aligned} \sigma _{{{\mathrm {fid}}}}=2.84^{+0.34}_{-0.31}=2.84^{+0.23}_{-0.22}\,\text {(stat)} ^{+0.26}_{-0.21}\,\text {(syst)} \,\text {fb} \end{aligned}$$

at $m_{{\mathrm{H}}}= 125.38\,\text {GeV}$. This can be compared to the SM expectation $\sigma _{{{\mathrm {fid}}}}^{{\mathrm {SM}}}=2.84\pm 0.15 \,\text {fb}$. The measured inclusive fiducial cross sections in different final states and integrated as a function of center-of-mass energy are shown in Fig. 16. The corresponding numerical values, including the decomposition of the uncertainties into statistical and systematic components, and the corresponding expected uncertainties, are given in Table 9.

**Table 9:** The measured inclusive fiducial cross section and $\pm 1$ standard deviation uncertainties for different final states and data-taking periods at $m_{{\mathrm{H}}}=125.38 \,\text {GeV}$. The statistical and systematic uncertainties are given separately for the inclusive measurements

	$2{\mathrm{e}} 2{{{\upmu }}_{\mathrm{}}^{\mathrm{}}}$ (fb)	$4{{{\upmu }}_{\mathrm{}}^{\mathrm{}}}$ (fb)	$4{\mathrm{e}}$ (fb)	Inclusive (fb)
2016	$1.22^{+0.38}_{-0.30}$	$0.89^{+0.22}_{-0.19}$	$1.07^{+0.44}_{-0.33}$	$3.19^{+0.68}_{-0.56}=3.19^{+0.48}_{-0.45}\,\text {(stat)} ^{+0.48}_{-0.33}\,\text {(syst)}$
2017	$1.64^{+0.41}_{-0.35}$	$0.82^{+0.21}_{-0.18}$	$0.56^{+0.29}_{-0.22}$	$3.01^{+0.60}_{-0.50}=3.01^{+0.44}_{-0.41}\,\text {(stat)} ^{+0.41}_{-0.27}\,\text {(syst)}$
2018	$1.17^{+0.27}_{-0.24}$	$0.66^{+0.15}_{-0.13}$	$0.73^{+0.24}_{-0.20}$	$2.57^{+0.42}_{-0.38}=2.57^{+0.33}_{-0.31}\,\text {(stat)} ^{+0.27}_{-0.23}\,\text {(syst)}$
2016–2018	$1.31^{+0.20}_{-0.19}$	$0.78^{+0.10}_{-0.10}$	$0.76^{+0.18}_{-0.16}$	$2.84^{+0.34}_{-0.31}=2.84^{+0.23}_{-0.22}\,\text {(stat)} ^{+0.26}_{-0.21}\,\text {(syst)}$

**Fig. 17:**
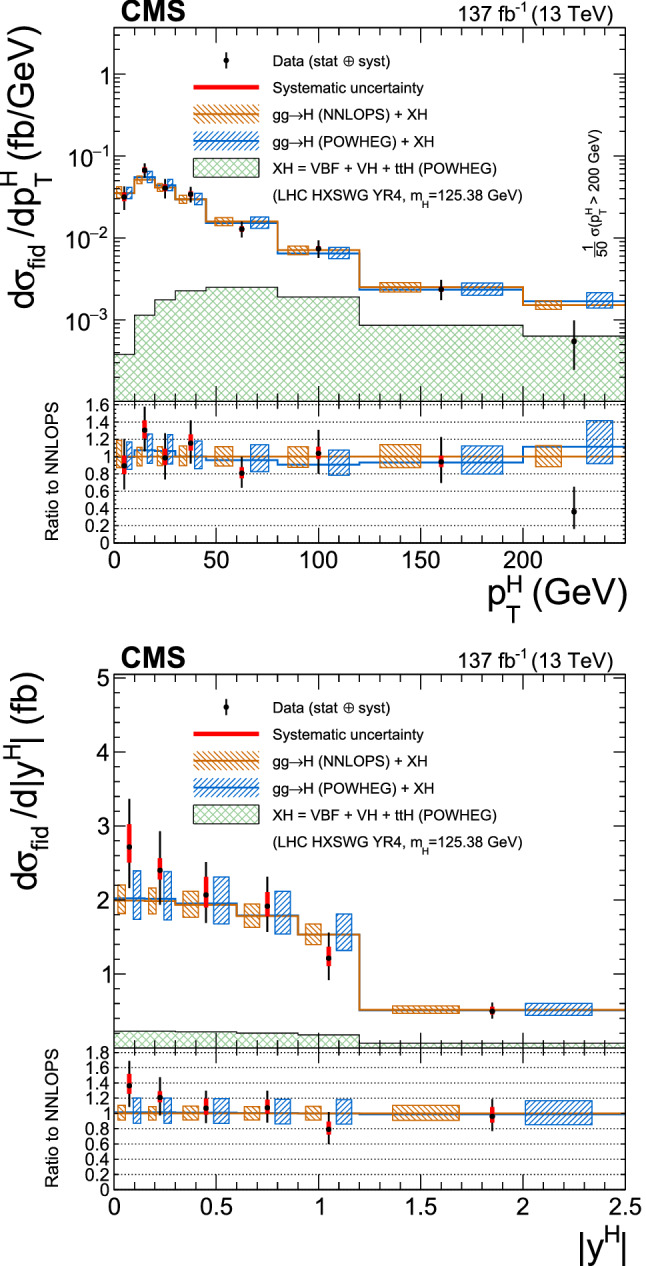
Differential cross sections as a function of $p_{{\mathrm {T}}} ^{{\mathrm{H}}}$ (upper) and $|y^{{\mathrm{H}}} |$ (lower). The acceptance and theoretical uncertainties in the differential bins are calculated using powheg. The sub-dominant component of the signal (${\mathrm {VBF}}+ {{\mathrm{V}}_{\mathrm{}}^{\mathrm{}}} {\mathrm{H}} + {{{\mathrm{t}}_{\mathrm{}}^{\mathrm{}}} {}{{{\overline{{{\mathrm{t}}_{\mathrm{}}^{\mathrm{}}}}}}_{\mathrm{}}^{\mathrm{}}}} {\mathrm{H}}$) is denoted as XH

**Fig. 18:**
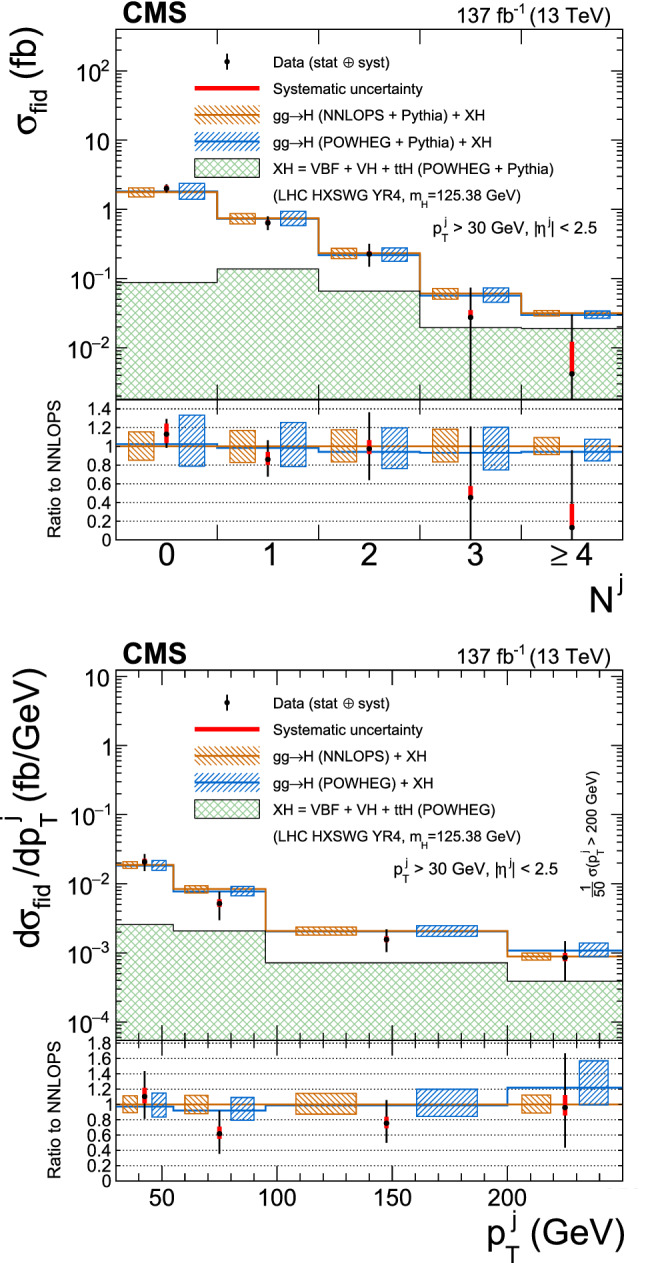
Differential cross sections as a function of the number of associated jets (upper), and $p_{{\mathrm {T}}}$ of the leading jet (lower). The acceptance and theoretical uncertainties in the differential bins are calculated using powheg. The sub-dominant component of the signal (${\mathrm {VBF}}+ {{\mathrm{V}}_{\mathrm{}}^{\mathrm{}}} {\mathrm{H}} + {{{\mathrm{t}}_{\mathrm{}}^{\mathrm{}}} {}{{{\overline{{{\mathrm{t}}_{\mathrm{}}^{\mathrm{}}}}}}_{\mathrm{}}^{\mathrm{}}}} {\mathrm{H}}$) is denoted as XH

The measured differential cross sections as a function of the H boson transverse momentum and rapidity are shown in Fig. 17. The corresponding numerical values are given in Tables 10 and 11 . Finally, the measured differential cross sections as a function of the number of associated jets and the transverse momentum of the leading jet are shown in Fig. 18. The corresponding numerical values are given in Tables 12 and 13.

For all the fiducial measurements the dominant systematic uncertainties are those on the lepton identification efficiencies and luminosity measurement, while the theoretical uncertainties are smaller. In order to assess the model dependence of the measurement, the unfolding procedure is repeated using different response matrices created by varying the relative fraction of each SM production mode within its experimental constraints. The uncertainty is negligible with respect to the experimental systematic uncertainties.

## Summary

Several measurements of the Higgs boson production in the four-lepton final state at $\sqrt{s} = 13 \,\text {TeV}$ have been presented, using data samples corresponding to an integrated luminosity of $137{\,\text {fb}^{-1}}$. Thanks to a large signal-to-background ratio and the complete reconstruction of the final state decay products, this channel enables a detailed study of the Higgs boson production properties. The measured signal strength modifier is $\mu =0.94 \pm 0.07\,\text {(stat)} ^{+0.07}_{-0.06}\,\text {(theo)} ^{+0.06}_{-0.05}\,(\text {exp})$ and the integrated fiducial cross section is measured to be $\sigma _{\text {fid}}=2.84^{+0.23}_{-0.22}\,\text {(stat)} ^{+0.26}_{-0.21}\,\text {(syst)} \,\text {fb}$ with a standard model prediction of $2.84 \pm 0.15 \,\text {fb}$ for the same fiducial region.. The signal strength modifiers for the main Higgs boson production modes are also reported. A new set of measurements, designed to quantify the different Higgs boson production processes in specific kinematical regions of phase space, have also been presented. The differential cross sections as a function of the transverse momentum and rapidity of the Higgs boson, the number of associated jets, and the transverse momentum of the leading associated jet are determined. All results are consistent, within their uncertainties, with the expectations for the standard model Higgs boson.

**Table 10:** The measured differential fiducial cross section and $\pm 1$ standard deviation uncertainties for the $p_{{\mathrm {T}}} ^{{\mathrm{H}}}$ observable at $m_{{\mathrm{H}}}=125.38 \,\text {GeV}$. The breakdown of the total uncertainty (unc.) into statistical and systematic components is given

Bin range ($\text {GeV}$)	$\hbox {d}\sigma _{{\mathrm {fid}}}$ (fb)	unc.	$\,\text {(stat)}$	$\,\text {(syst)}$
0–10	0.32	$^{+0.11}_{-0.10}$	$^{+0.10}_{-0.09}$	$^{+0.04}_{-0.03}$
10–20	0.67	$^{+0.14}_{-0.13}$	$^{+0.13}_{-0.12}$	$^{+0.06}_{-0.05}$
20–30	0.41	$^{+0.12}_{-0.10}$	$^{+0.11}_{-0.10}$	$^{+0.04}_{-0.04}$
30–45	0.51	$^{+0.12}_{-0.10}$	$^{+0.11}_{-0.10}$	$^{+0.04}_{-0.04}$
45–80	0.45	$^{+0.10}_{-0.09}$	$^{+0.10}_{-0.09}$	$^{+0.04}_{-0.03}$
80–120	0.30	$^{+0.08}_{-0.07}$	$^{+0.07}_{-0.07}$	$^{+0.02}_{-0.02}$
120–200	0.19	$^{+0.06}_{-0.05}$	$^{+0.06}_{-0.05}$	$^{+0.01}_{-0.01}$
200–13000	0.03	$^{+0.02}_{-0.02}$	$^{+0.02}_{-0.01}$	$^{+0.00}_{-0.00}$

**Table 11:** The measured differential fiducial cross section and $\pm 1$ standard deviation uncertainties for the $|y^{{\mathrm{H}}} |$ observable at $m_{{\mathrm{H}}}=125.38 \,\text {GeV}$. The breakdown of the total uncertainty (unc.) into statistical and systematic components is given

Bin range	$\hbox {d}\sigma _{{\mathrm {fid}}}$ (fb)	unc.	$\,\text {(stat)}$	$\,\text {(syst)}$
0.0–0.15	0.41	$^{+0.10}_{-0.08}$	$^{+0.09}_{-0.08}$	$^{+0.05}_{-0.03}$
0.15–0.3	0.36	$^{+0.08}_{-0.07}$	$^{+0.07}_{-0.07}$	$^{+0.03}_{-0.02}$
0.3–0.6	0.62	$^{+0.13}_{-0.11}$	$^{+0.11}_{-0.10}$	$^{+0.07}_{-0.05}$
0.6–0.9	0.57	$^{+0.12}_{-0.10}$	$^{+0.10}_{-0.10}$	$^{+0.06}_{-0.04}$
0.9–1.2	0.36	$^{+0.10}_{-0.09}$	$^{+0.09}_{-0.08}$	$^{+0.05}_{-0.03}$
1.2–2.5	0.64	$^{+0.15}_{-0.13}$	$^{+0.13}_{-0.12}$	$^{+0.08}_{-0.05}$

**Table 12:** The measured differential fiducial cross section and $\pm 1$ standard deviation uncertainties for the $N^{\text {j}}$ observable at $m_{{\mathrm{H}}}=125.38 \,\text {GeV}$. The breakdown of the total uncertainty (unc.) into statistical and systematic components is given

Bin range	$\hbox {d}\sigma _{{\mathrm {fid}}}$ (fb)	unc.	$\,\text {(stat)}$	$\,\text {(syst)}$
0	2.00	$^{+0.29}_{-0.26}$	$^{+0.21}_{-0.20}$	$^{+0.20}_{-0.17}$
1	0.64	$^{+0.15}_{-0.14}$	$^{+0.14}_{-0.13}$	$^{+0.06}_{-0.04}$
2	0.23	$^{+0.09}_{-0.08}$	$^{+0.09}_{-0.08}$	$^{+0.02}_{-0.01}$
3	0.03	$^{+0.05}_{-0.03}$	$^{+0.05}_{-0.03}$	$^{+0.01}_{-0.00}$
$\ge$4	0.00	$^{+0.03}_{-0.00}$	$^{+0.03}_{-0.00}$	$^{+0.01}_{-0.00}$

**Table 13:** The measured differential fiducial cross section and $\pm 1$ standard deviation uncertainties for the $p_{{\mathrm {T}}} ^\text {j}$ observable at $m_{{\mathrm{H}}}=125.38 \,\text {GeV}$. The breakdown of the total uncertainty (unc.) into statistical and systematic components is given

Bin range ($\text {GeV}$)	$\hbox {d}\sigma _{{\mathrm {fid}}}$ (fb)	unc.	$\,\text {(stat)}$	$\,\text {(syst)}$
30–55	0.52	$^{+0.16}_{-0.14}$	$^{+0.15}_{-0.13}$	$^{+0.05}_{-0.04}$
55–95	0.21	$^{+0.10}_{-0.09}$	$^{+0.10}_{-0.09}$	$^{+0.03}_{-0.02}$
95–200	0.16	$^{+0.07}_{-0.06}$	$^{+0.06}_{-0.05}$	$^{+0.02}_{-0.01}$
200–13000	0.04	$^{+0.03}_{-0.02}$	$^{+0.03}_{-0.02}$	$^{+0.01}_{-0.01}$

## Data Availability

This manuscript has no associated data or the data will not be deposited. [Authors’ comment: This is our standard statement: Release and preservation of data used by the CMS Collaboration as the basis for publications is guided by the CMS policy as written in its document “CMS data preservation, re-use and open access policy” (https://cmsdocdb.cern.ch/cgi-bin/PublicDocDB/RetrieveFile?docid=6032&filename=CMSDataPolicyV1.2.pdf&version=2)].
